# Multi-omics characterization of anoikis-related hub genes and macrophage-centered immune persistence with validation of HAVCR2 in atherosclerosis

**DOI:** 10.3389/fimmu.2026.1918938

**Published:** 2026-09-11

**Authors:** Qiang Shen, Zhengfeng Fan, Jincheng Hou, Fei Li, Yixuan Wang, Zongtao Liu, Chen Jiang, Nianguo Dong

**Affiliations:** Department of Cardiovascular Surgery, Wuhan Union Hospital, Tongji Medical College, Huazhong University of Science and Technology, Wuhan, China

**Keywords:** anoikis, atherosclerosis, HAVCR2, machine learning, macrophages, plaque progression, single-cell RNA sequencing

## Abstract

**Background:**

Atherosclerosis (AS) is a chronic inflammatory vascular disease characterized by immune dysregulation, metabolic disturbance, and progressive vascular remodeling. Although anoikis resistance has been extensively investigated in cancer biology, its involvement in immune-cell persistence and plaque progression in AS remains poorly understood.

**Methods:**

We integrated two publicly available bulk transcriptomic datasets (GSE100927 and GSE28829) to identify differentially expressed genes (DEGs) and performed weighted gene co-expression network analysis (WGCNA) to determine disease-associated gene modules. Anoikis-related candidate genes were obtained by intersecting DEGs, WGCNA-derived module genes, and a curated anoikis-related gene set, and were further prioritized using least absolute shrinkage and selection operator (LASSO) regression and random forest algorithms. Immune infiltration and cell type–specific expression patterns were evaluated using CIBERSORT and single-cell RNA sequencing (scRNA-seq) analyses. The expression of the leading candidate was validated using both *in vitro* and *in vivo* experiments.

**Results:**

We identified eight core anoikis-related hub genes—HAVCR2, CD36, PLAU, TNF, PYCARD, SERPINA1, BLNK, and CHI3L1—that were closely associated with AS. These genes were enriched in inflammatory and metabolic pathways and showed strong diagnostic performance across validation analyses, with AUC values greater than 0.83. Immune deconvolution and single-cell transcriptomic analyses revealed that the anoikis-related signature was predominantly localized to macrophage and T-cell compartments within atherosclerotic lesions. Among the identified candidates, HAVCR2 showed the most consistent disease-associated pattern. HAVCR2 expression was markedly increased in oxLDL-stimulated THP-1-derived macrophages and was enriched in macrophage-rich atherosclerotic lesions in high-fat diet–fed ApoE^−/−^ mice, supporting its association with lipid-induced macrophage activation and vascular inflammation.

**Conclusion:**

This study defines a macrophage-centered anoikis-related immune signature in atherosclerosis and identifies HAVCR2, together with CD36, PLAU, TNF, PYCARD, SERPINA1, BLNK, and CHI3L1, as potential biomarkers and candidate targets for future diagnostic and therapeutic investigation.

## Introduction

Atherosclerosis (AS) underlies most ischemic cardiovascular events and remains a leading driver of global mortality and disability. Recent global estimates highlight the persistent and increasing burden of cardiovascular disease, for which AS is the predominant pathological substrate (1, 2). Mechanistically, AS is initiated and propagated by endothelial dysfunction, subendothelial retention of apolipoprotein B–containing lipoproteins with subsequent modification/oxidation, and progressive vascular wall remodeling (3, 4). As lesions evolve, vascular smooth muscle cells (VSMCs) undergo phenotypic modulation and contribute to fibrous-cap structure and extracellular matrix (ECM) dynamics that influence plaque stability (5, 6).

Inflammation is not merely a bystander but a causal axis in AS. Both innate and adaptive immune responses shape lesion initiation, progression, and clinical destabilization, with macrophages and T cells constituting dominant immune compartments within plaques (7). The clinical relevance of inflammatory signaling is underscored by anti-inflammatory intervention trials demonstrating that dampening cytokine-driven pathways can reduce recurrent cardiovascular events independent of lipid lowering (8). In parallel, cellular loss and remodeling of plaque-stabilizing VSMCs can promote features of plaque vulnerability, emphasizing the importance of regulated cell survival programs in advanced disease (9).

Anoikis is a specialized form of programmed cell death triggered by the loss of appropriate cell–ECM attachment and is tightly regulated by integrin-mediated adhesion signalling (10). Although anoikis has been studied extensively in cancer biology, previous vascular studies have also linked adhesion-dependent survival to endothelial integrity and VSMC maintenance (5). Notably, Escate et al. showed that LDL exposure affects monocyte-to-macrophage differentiation, cellular adhesion, and anoikis, providing direct evidence that anoikis-related processes are relevant to macrophage biology in atherosclerosis (11). Nevertheless, the system-level transcriptional organization of anoikis-related genes across atherosclerotic lesions, their distribution among plaque cell states, and their relationships with the lesional immune microenvironment remain incompletely characterized.

High-throughput transcriptomics and systems-level modeling provide an opportunity to identify disease-relevant molecular programs beyond single-gene effects, enabling inference of coordinated networks that connect molecular states to disease phenotypes (12). Moreover, single-cell RNA sequencing has revealed extensive cellular heterogeneity and state plasticity within atherosclerotic lesions, including diverse macrophage activation states and VSMC-derived modulated phenotypes (13–15). Complementary computational approaches can further quantify immune composition from bulk data and support mechanistic integration with candidate gene signatures (16).

In this study, we integrated bulk transcriptomic and single-cell RNA-sequencing datasets to characterize anoikis-related transcriptional signatures and their cellular distribution in atherosclerosis. Although anoikis has been linked to AS-relevant macrophage biology (11), its transcriptional programs and cell-type-specific localization within plaques remain incompletely characterized. A previous network-based analysis using the same bulk datasets had already identified HAVCR2 as a hub gene associated with advanced atherosclerotic plaques (17). Building on this precedent, we identified eight anoikis-related candidate genes, examined their relationships with the immune microenvironment, and mapped their expression across plaque cell types and macrophage states. We further characterized anoikis-related and inflammatory transcriptional activity, inferred transcription-factor networks and macrophage-state trajectories, and assessed HAVCR2 expression following oxLDL stimulation and in ApoE−/− mouse lesions. Thus, our study extends previous findings by placing HAVCR2 and the other candidate genes within an integrated anoikis-related, immune, and single-cell transcriptional framework.

## Materials and methods

### Data acquisition and differential expression analysis

Public transcriptomic datasets GSE100927, GSE28829, and GSE43292 were obtained from GEO. In GSE100927, atherosclerotic arterial samples were compared with non-atherosclerotic control arterial samples. GSE43292 comprised 32 atherosclerotic carotid plaque samples and 32 patient-matched macroscopically intact adjacent carotid tissue samples, with the adjacent tissues serving as the reference group. GSE28829 contained no healthy controls; its 13 early and 16 advanced carotid plaque samples were analysed as an early-to-advanced plaque progression contrast, with early plaques serving as the reference group. The GSE100927 dataset includes atherosclerotic and control arterial samples from three distinct vascular territories: carotid, femoral, and infra-popliteal arteries. Differential expression was performed separately within each dataset using limma, with P < 0.05 and |FC| > 2 as the prespecified thresholds.

### Weighted gene co-expression network analysis

To identify core gene modules associated with AS pathogenesis, we performed WGCNA on the GSE100927 and GSE28829 cohorts. To ensure a scale-free network topology, cohort-specific optimal soft-thresholding powers (β = 5 and β = 26, respectively) were applied to construct the weighted adjacency matrices. Module eigengenes (MEs) were subsequently computed to correlate distinct co-expression modules with clinical traits (AS vs. CTR), allowing the isolation of the most highly disease-associated modules for downstream analysis.

### Functional enrichment analysis

Functional enrichment analyses were performed using the clusterProfiler R package (version 4.18.4). Gene Ontology (GO) and Kyoto Encyclopedia of Genes and Genomes (KEGG) enrichment analyses were conducted using the org.Hs.eg.db annotation package and the KEGG human pathway database (organism = “hsa”), respectively. Gene set enrichment analysis (GSEA) was performed for both GSE100927 and GSE28829 using the Hallmark gene set collection from the Molecular Signatures Database (MSigDB; h.all.v2024.1.Hs.symbols.gmt), with genes ranked in descending order according to log2 fold change. P values were adjusted for multiple comparisons using the Benjamini–Hochberg method.

### Machine learning-based feature selection

A reference set of 919 anoikis-related genes (ARGs) was obtained from GeneCards and intersected with the DEGs and WGCNA phenotype-associated modules to identify candidate anoikis-related DEGs. Candidate features were prioritized using LASSO logistic regression and random forest. LASSO was implemented using glmnet (v5.0) with a binomial family, α = 1, and 100 λ values. The regularization parameter was selected using 5-fold cross-validation for GSE100927 and 10-fold cross-validation for GSE28829, and genes with non-zero coefficients at *lambda.min* were retained. Random forest analysis was performed using randomForest (v4.7.1.2), with 15 trees evaluated for GSE100927 and 10 trees for GSE28829; feature importance was assessed using mean decrease in Gini impurity. Feature-selection stability was further evaluated using 1,000 bootstrap resamples with replacement, and selection frequencies were calculated separately for LASSO and random forest. The final candidate genes were identified from the intersection of features selected by the two algorithms.

### Diagnostic model construction and external validation

The diagnostic efficacy of the core hub genes was quantified via Receiver Operating Characteristic (ROC) curve analysis using the *pROC* R package, with the Area Under the Curve (AUC) serving as the primary evaluation metric. To facilitate individualized clinical risk assessment, a predictive multi-gene nomogram model was constructed utilizing the *rms* package. Finally, the expression patterns and diagnostic robustness of these hub genes were independently corroborated in the external GSE43292 validation cohort.

### Immune microenvironment deconvolution

To characterize the immune-cell composition of atherosclerotic plaques, the relative fractions of 22 immune-cell subsets were estimated using the CIBERSORT deconvolution algorithm with the LM22 signature matrix. CIBERSORT was run with 100 permutations and quantile normalization enabled. In accordance with the standard CIBERSORT filtering criterion, only samples with a deconvolution P value < 0.05 were retained for subsequent immune-cell composition and correlation analyses. Associations between candidate-gene expression and estimated immune-cell fractions were evaluated using the cor.test function in R, with the default Pearson correlation method. Statistical significance was assessed using the resulting nominal two-sided P values, with P < 0.05 considered statistically significant. No multiple-comparison adjustment was applied.

### Single-Cell RNA sequencing data integration and processing

Five human vascular single-cell RNA sequencing (scRNA-seq) datasets (GSE131778, GSE155512, GSE159677, GSE216860, and GSE245373) were obtained from the GEO database and integrated for analysis. During Seurat object construction, genes detected in fewer than three cells and cells with fewer than 200 detected genes were excluded. Mitochondrial, ribosomal, and hemoglobin transcript proportions were subsequently assessed, and cells with mitochondrial transcript proportions ≥25% or hemoglobin transcript proportions ≥1% were removed. Because the datasets differed in sequencing depth and experimental protocol, no uniform UMI or ribosomal-transcript threshold was imposed. Following log normalization, variable-feature selection, data scaling, and principal component analysis, dataset-associated effects were corrected using Harmony with dataset identity as the batch variable. The first 15 Harmony dimensions were used for UMAP visualization, nearest-neighbor graph construction, and unsupervised Louvain clustering at a resolution of 0.2. Dataset-split UMAP panels based on the same Harmony-corrected embedding were generated to examine the contribution and mixing of cells from each dataset across the annotated cell populations. Samples were assigned to the atherosclerotic or normal/reference group according to their original tissue annotations and pathological contexts. The atherosclerotic group included atherosclerotic coronary artery, carotid plaque, and atherosclerotic core (AC) tissues. The normal/reference group comprised non-atherosclerotic ascending aortic tissues from organ donors in GSE216860 and patient-matched proximal adjacent (PA) carotid tissues from GSE159677. In GSE159677, PA tissues were collected proximal to the corresponding AC lesions and served as within-patient reference tissues. Thus, “normal/reference” denotes an analytical group comprising both non-atherosclerotic donor tissues and lesion-adjacent reference tissues, rather than exclusively arteries from healthy individuals. GSE245373 was generated from pre-sorted viable CD45+ cells and therefore contributed immune, but not stromal, populations to the integrated atlas. Given the differences in anatomical origin, cell-selection strategy, and tissue-processing protocol, pooled cell-type proportions were interpreted as descriptive compositional patterns rather than estimates of absolute biological abundance or disease-specific effects. To quantify the anoikis-related transcriptional signature at single-cell resolution, Anoikis-score1 was calculated from normalized RNA-expression data using Seurat’s `AddModuleScore` function with default parameters. The score was based on the eight candidate genes identified through the bulk transcriptomic and machine-learning analyses: CHI3L1, HAVCR2, PYCARD, SERPINA1, BLNK, CD36, PLAU, and TNF. Anoikis-score1 represents the average normalized expression of this eight-gene signature relative to expression-matched control-gene sets. A higher score therefore indicates greater relative expression of the signature within an individual cell, rather than direct measurement of functional anoikis or cell death. Downstream analyses, including cell-type-specific enrichment and differential-expression analyses, were performed using the SCP pipeline.

### Macrophage subclustering, regulon inference, and pseudotime trajectory analysis

The macrophage lineage was computationally extracted and subclustered at a resolution of 0.2 to characterize macrophage heterogeneity. Transcription-factor activities and regulons were inferred using pySCENIC (v0.12.1). The cisTarget enrichment analysis was performed using the human hg19 TSS-centered 10-kb ranking database (hg19-tss-centered-10kb-10species.mc9nr.genes_vs_motifs.rankings.feather) and the human motif-annotation database (motifs-v10nr_clust-nr.hgnc-m0.001-o0.0.tbl). Macrophage-state trajectories were subsequently inferred using the Slingshot R package, with Prolif_Mac specified as the root state based on previous evidence regarding macrophage-state transitions in atherosclerosis. Candidate-gene expression was then examined along the inferred pseudotime trajectories.

### *In vitro* macrophage polarization and foam cell model

Human THP-1 monocytes were cultured in RPMI-1640 medium (Gibco) supplemented with 10% fetal bovine serum (HYcezmbio) at 37 °C in a humidified atmosphere containing 5% CO_2_. To induce macrophage differentiation, THP-1 cells were stimulated with 100 ng/mL phorbol 12-myristate 13-acetate (PMA) for 48 h, generating an M0-like macrophage phenotype. An *in vitro* lipid-induced atherosclerosis model was then established by exposing the differentiated macrophages to 50 μg/mL oxidized low-density lipoprotein (oxLDL) for 24 h. HAVCR2 protein expression was subsequently quantified by Western blotting. Six independently cultured dishes were analyzed per condition, comprising six untreated control dishes and six oxLDL-treated dishes. Each dish was independently cultured, treated, harvested, and analyzed as an independent experimental replicate at the culture level.

### Western blotting

Total cellular proteins were extracted, quantified, and resolved via SDS-PAGE. Following electrophoretic separation, proteins were transferred onto PVDF membranes. The membranes were blocked with 5% non-fat milk in TBST for 1 h at room temperature and incubated overnight at 4 °C with primary antibodies against HAVCR2 (1:1000, Proteintech, Cat. No. 60355-1-Ig, AB_2881464), GAPDH (1:5000, Servicebio, Cat. No. GB15004-100), and F4/80 (Proteintech, Cat No. 28463-1-AP, AB_2881149). After stringent TBST washing, the membranes were incubated with HRP-conjugated secondary antibodies (1:5000) for 1 h at room temperature. Immunoreactive bands were visualized using an enhanced chemiluminescence (ECL) system, and the relative protein expression of HAVCR2 was quantified using ImageJ software, normalized to the GAPDH endogenous control.

### *In vivo* atherosclerosis model and dietary intervention

All mice used in this study were purchased from Wuhan Shulaibao Biotechnology Co., Ltd. Twelve male ApoE knockout (ApoE−/−) mice aged 6–8 weeks were randomly assigned to two dietary groups, with six mice per group. The normal-chow-diet (NCD) group was fed standard laboratory chow, whereas the high-fat-diet (HFD) group was fed a high-fat, high-cholesterol diet containing 40% kcal from fat and 1.25% cholesterol (Research Diets, Inc., Cat. No. D12108C) for 12 weeks to induce atherosclerotic lesions. Each mouse represented an independent biological replicate. All animals were housed in a specific pathogen-free (SPF) facility under standard controlled conditions (12-h light/dark cycle, 22 ± 2 °C, 50 ± 5% humidity) with ad libitum access to food and water. At the end of the 12-week dietary intervention, mice were deeply anesthetized with sodium pentobarbital (50 mg/kg, intraperitoneally). Adequate anesthesia was confirmed by the absence of pedal withdrawal and corneal reflexes. While under deep anesthesia, mice were euthanized by cervical dislocation, followed by tissue collection for subsequent analyses. All animal procedures and experimental protocols were approved by the Institutional Animal Care and Use Committee (IACUC) of Tongji Medical College, Huazhong University of Science and Technology (IACUC approval no. [2024] 3064) and were conducted in strict accordance with the Guide for the Care and Use of Laboratory Animals (18).

### Tissue preparation and hematoxylin-eosin staining

Following euthanasia, mouse aortas were carefully excised, fixed in 4% paraformaldehyde (PFA) for 24 h at 4 °C, dehydrated through a graded ethanol series, and embedded in paraffin. Serial sections (4–5 μm) were deparaffinized, rehydrated, and subjected to standard H&E staining. Morphological changes and atherosclerotic lesion areas within the aortic wall were subsequently evaluated and photographed under a light microscope.

### Oil red O staining

To assess macroscopic lipid deposition, the isolated aortas were gently rinsed with phosphate-buffered saline (PBS) and fixed in 4% PFA at 4 °C for 1 h. Following pre-treatment with 60% isopropanol, the whole aortas were incubated in freshly filtered Oil Red O working solution (3:2 ratio of stock to distilled water) for 15–20 min in the dark at room temperature. After differentiation with 60% isopropanol to remove non-specific background staining, the aortas were longitudinally opened, mounted with glycerol gelatin, and imaged. The percentage of the lipid-laden area (red) relative to the total aortic surface area was quantified using image analysis software.

### Masson’s trichrome staining

To evaluate extracellular matrix remodeling and collagen distribution, adjacent paraffin-embedded aortic sections (4–5 μm) were deparaffinized and stained utilizing a Masson’s trichrome protocol. Briefly, sections were sequentially treated with Weigert’s iron hematoxylin, ponceau-fuchsin, and aniline blue. This differential staining distinctly highlighted collagen fibers (blue) against the cytoplasm and smooth muscle fibers (red).

### Picrosirius red staining

To further characterize collagen fiber subtypes and structural organization, aortic sections were stained with Sirius Red solution for 1 h following a brief hematoxylin nuclear counterstain. The sections were analyzed under a polarized light microscope, where distinct birefringence patterns indicated specific collagen types (e.g., type I and type III collagen). The organized collagen areas were subsequently quantified utilizing image analysis software.

### Immunofluorescence staining

To localize protein expression and cellular colocalization within atherosclerotic plaques, adjacent paraffin-embedded aortic sections (4–5 μm) were deparaffinized, rehydrated, and subjected to heat-induced antigen retrieval in citrate buffer (pH 6.0). Non-specific binding was blocked with 5% bovine serum albumin (BSA) for 30 min at room temperature. The sections were subsequently incubated overnight at 4 °C with primary antibodies against F4/80 (1:100; Proteintech, Cat. No. 28463-1-AP; RRID: AB_2881149) and HAVCR2 (1:100; Proteintech, Cat. No. 60355-1-Ig; RRID: AB_2881464). After washing with PBS, the sections were incubated with an Alexa Fluor 488-conjugated secondary antibody (1:200; Servicebio, Cat. No. GB25303) for detection of the rabbit-derived F4/80 primary antibody and a Cy3-conjugated donkey anti-mouse IgG secondary antibody (1:200; Servicebio, Cat. No. GB21401) for detection of the mouse-derived HAVCR2 primary antibody for 1 h at room temperature in the dark. Nuclei were counterstained with DAPI for 5 min, and the sections were mounted using an antifade mounting medium. Fluorescence images were acquired using a fluorescence microscope, and the mean fluorescence intensity and colocalization area were quantified using image-analysis software. Quantification of the histological and immunofluorescence images was performed with the investigators blinded to the dietary group assignments.

### Statistical analysis

All *in vitro* and *in vivo* experimental data were analyzed using GraphPad Prism 9 software (GraphPad Software, Inc., San Diego, CA, USA) and are presented as the mean ± standard deviation (SD). Data distribution normality was assessed utilizing the Shapiro-Wilk test. For normally distributed continuous variables, statistical comparisons were performed using an unpaired two-tailed Student’s t-test (for two groups) or a one-way analysis of variance (ANOVA) (for multiple groups). For data that violated the normality assumption, the non-parametric Mann-Whitney U test was applied. A two-sided P-value of < 0.05 was considered to indicate statistical significance.

## Results

### Immune activation and loss of smooth muscle contractility converge on a core immunometabolic axis in atherosclerosis

Global transcriptomic profiling of two independent cohorts (GSE100927 and GSE28829) revealed a significant biphasic transcriptional reprogramming in AS (**Supplementary Figure S1**). Diseased tissues exhibited marked upregulation of innate immunity, ECM turnover (MMP9, ACP5), and immunometabolic activation, associated with pathways such as TNF-α/NF-κB, IL6/JAK/STAT3, and glycolysis (**Supplementary Figure S2**). Conversely, genes governing VSMC contractility (MYBL1, ACTN2) and oxidative phosphorylation were universally suppressed. This robust functional signature confirms a pathological ecosystem shift from structural vascular homeostasis to a leukocyte-dominated inflammatory state.

To systematically distill these broad transcriptomic alterations into highly coordinated functional networks, we performed WGCNA. To guarantee topological robustness and eliminate confounding technical artifacts, preliminary hierarchical clustering was applied. This quality control step led to the stringent exclusion of two aberrant transcriptomes (GSM2669641 and GSM2696632) from the GSE100927 dataset, while the GSE28829 cohort exhibited compact intra-cohort clustering necessitating no outlier removal (**Supplementary Figure S3**).

**Figure 1:**
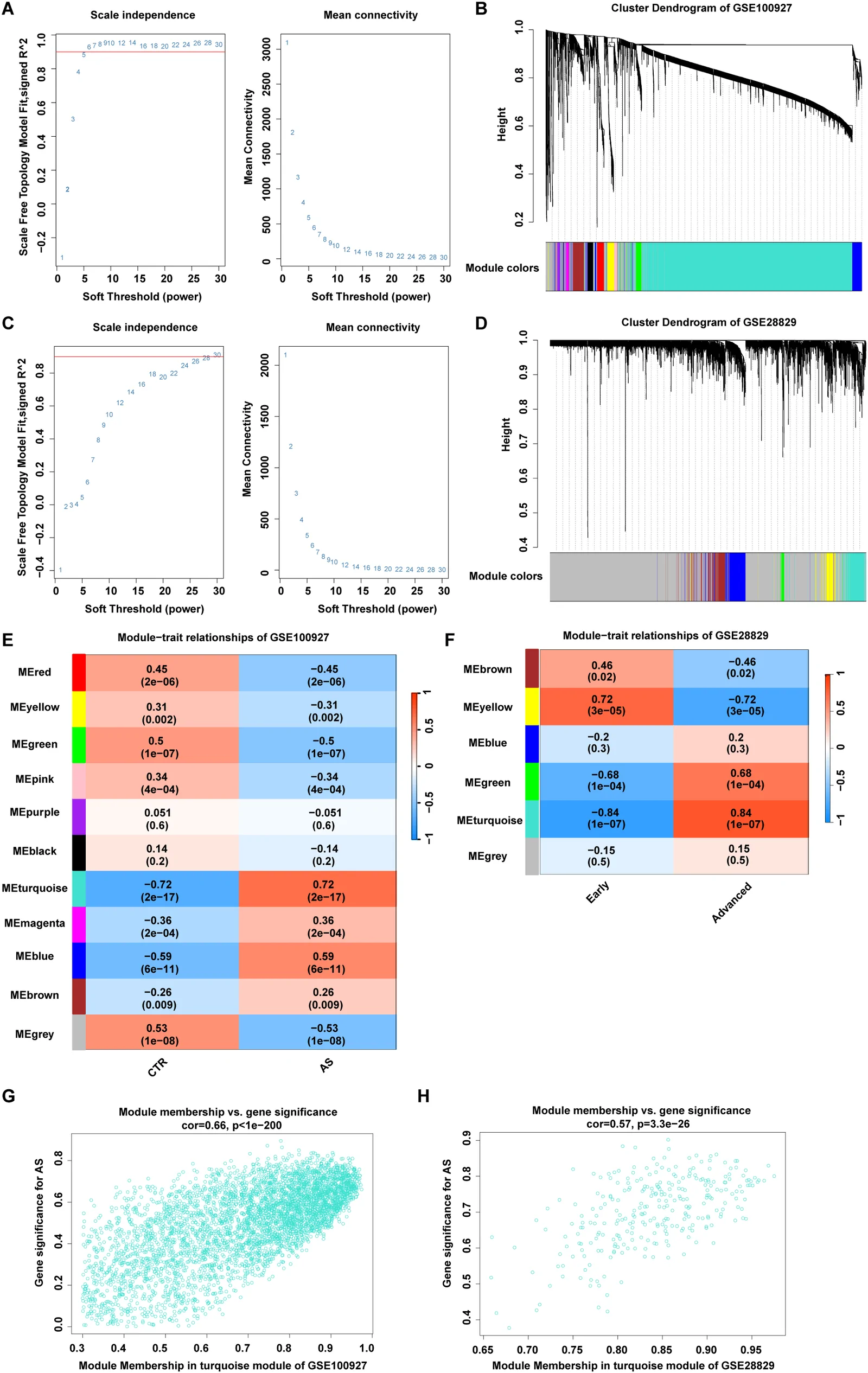
WGCNA identifies AS-associated co-expression modules. **(A)** Soft-thresholding power (β) analysis for GSE100927. The left panel shows scale-free topology model fit (signed R²) across candidate β values; the right panel shows mean connectivity. The red horizontal line denotes the target R² threshold used for β selection. **(B)** Gene clustering dendrogram of GSE100927 based on topological overlap, with module assignments shown as color bars. **(C)** Soft-thresholding power (β) analysis for GSE28829. **(D)** Gene clustering dendrogram of GSE28829 with corresponding module color assignments. **(E)** Module–trait relationships in GSE100927. Heatmap displays Pearson correlations between module eigengenes (rows) and phenotype (columns: AS and CTR); P values are shown in parentheses. **(F)** Module–trait relationships in GSE28829. **(G)** Scatter plot of module membership (MM) versus gene significance (GS) for AS within the turquoise module of GSE100927; correlation coefficient and P value are indicated. **(H)** Scatter plot of MM versus GS for AS within the turquoise module of GSE28829.

By applying cohort-specific soft-thresholding powers (β = 5 and β = 26) to maintain a scale-free topology (R^2^), we constructed adjacency matrices and identified a central ‘turquoise’ module that emerged as the most significantly upregulated network in AS across both datasets (GSE100927: r = 0.72, P = 2 × 10^-17^; GSE28829: r = 0.84, P = 1 × 10^-7^) (**Figures 1A–F**). Furthermore, intramodular analysis demonstrated a positive correlation between Module Membership (MM) and Gene Significance (GS) for AS within this turquoise network (GSE100927: r = 0.66, P < 1 × 10^-200^; GSE28829: r = 0.57, P = 3.3 × 10^-26^) (**Figures 1G, H**). The observed MM–GS correlation indicates that the turquoise module contains genes associated with the corresponding phenotypic contrasts, providing a network-level basis for the subsequent prioritization of anoikis-related candidate genes.

### Core anoikis regulators dictate the pathogenic rewiring of atherosclerosis

**Figure 2:**
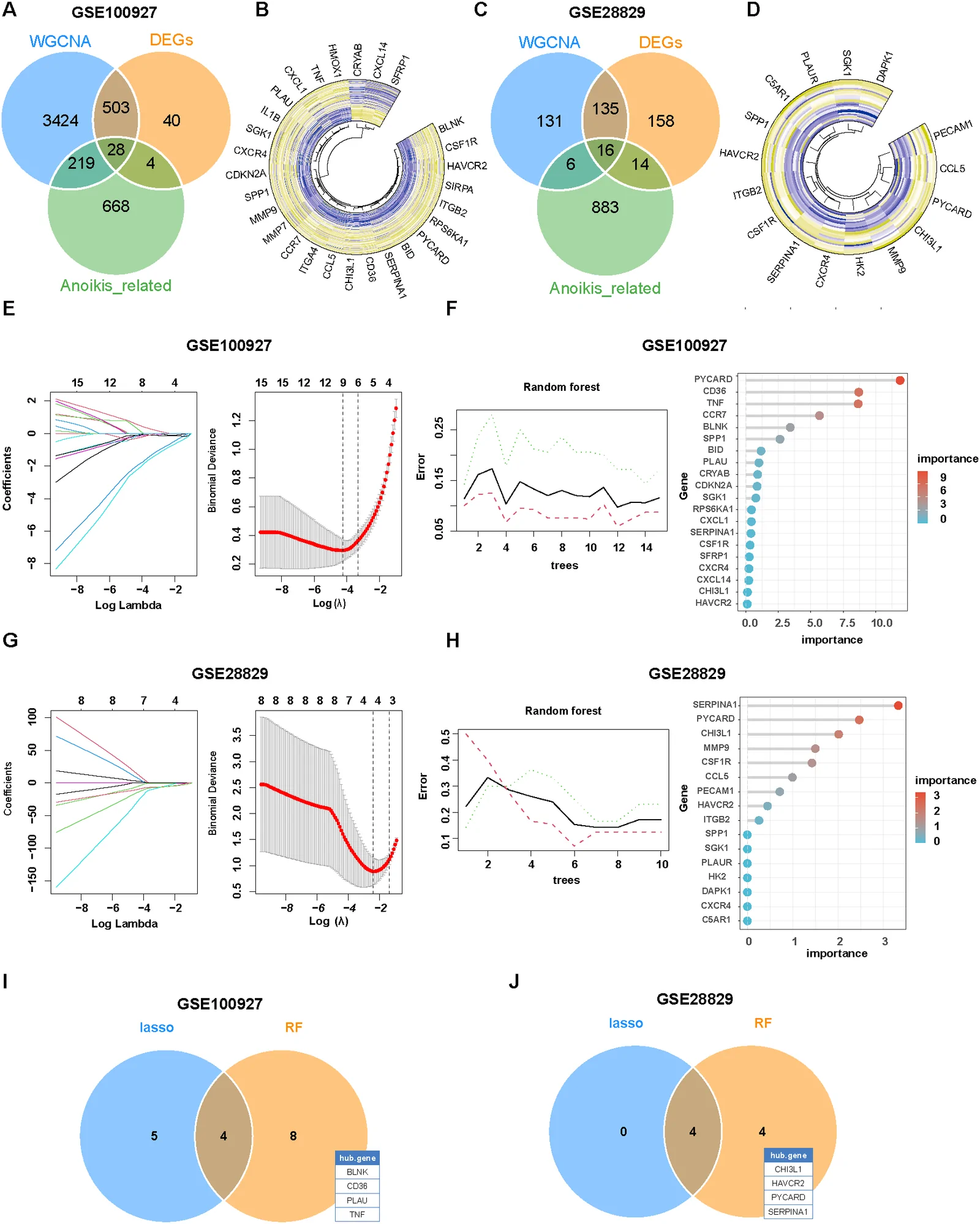
Integrated screening of AS-associated anoikis-related hub genes. **(A)** Venn diagram in GSE100927 showing the overlap among WGCNA-derived disease-associated module genes, DEGs (AS vs CTR), and ARGs. **(B)** Circular heatmap of the 28 intersected candidate genes in GSE100927. Samples are arranged by hierarchical clustering; the color gradient represents standardized relative expression levels across samples. **(C)** Venn diagram in GSE28829 showing the overlap among WGCNA module genes, DEGs, and ARGs. **(D)** Circular heatmap of the 16 intersected candidate genes in GSE28829. **(E)** LASSO logistic regression feature selection in GSE100927, including coefficient paths versus log(λ) (left) and cross-validation curve for λ selection (right). **(F)** RF analysis in GSE100927. Left: model error versus number of trees; right: variable-importance ranking of candidate genes. **(G)** LASSO feature selection in GSE28829. **(H)** RF analysis in GSE28829. **(I)** Overlap between LASSO-selected genes and RF-prioritized genes in GSE100927; the intersection defines four hub genes. **(J)** Overlap between LASSO-selected genes and RF-prioritized genes in GSE28829; the intersection defines four hub genes.

To pinpoint anoikis-related genes (ARGs) driving atherosclerotic transcriptional remodeling, we developed an integrative feature-selection pipeline. We first defined candidate AS-associated ARGs by intersecting three distinct gene spaces: (i) DEGs, (ii) WGCNA-derived disease-associated modules, and (iii) a curated ARG database. This stringent tri-directional screening yielded 28 and 16 candidate ARGs in the GSE100927 and GSE28829 cohorts, respectively (**Figures 2A, C**). Circular heatmaps confirmed that these candidates exhibited highly coordinated, non-random expression patterns within each cohort, indicative of synchronized disease-linked regulatory networks rather than isolated single-gene effects (**Figures 2B, D**).

To further distill these candidates into critical diagnostic or mechanistic hubs, we applied two independent machine learning algorithms: LASSO logistic regression and RF. LASSO identified optimal sparse feature subsets by applying an L1 penalty (λ) via cross-validation (**Figures 2E, G**), while RF ranked genes based on their variable importance for discriminating AS from control tissues (**Figures 2F, H**).

The final candidate genes were defined by independently intersecting the features selected by LASSO and Random Forest within GSE100927 and GSE28829, respectively, rather than by intersecting results across the two datasets. This dual-algorithm approach identified four disease-associated candidate ARGs in GSE100927 (BLNK, CD36, PLAU, and TNF; **Figure 2I**) and four plaque-progression-associated candidate ARGs in GSE28829 (CHI3L1, HAVCR2, PYCARD, and SERPINA1; **Figure 2J**). Their selection stability was subsequently evaluated using 1,000 stratified bootstrap resamples, with LASSO and Random Forest analyses repeated in each iteration (**Supplementary Figure S4**). The resulting selection frequencies provided resampling-based support for the robustness of these candidates while also showing algorithm-specific variation in their stability. Collectively, these analyses prioritized eight candidate ARGs for subsequent validation and functional investigation.

### Anoikis regulators serve as robust clinical biomarkers for atherosclerotic disease

To systematically assess the diagnostic potential of the selected anoikis-related hub genes in AS, we conducted ROC curve analyses across two internal validation datasets (GSE100927 and GSE28829) and an independent external cohort (GSE43292).

**Figure 3:**
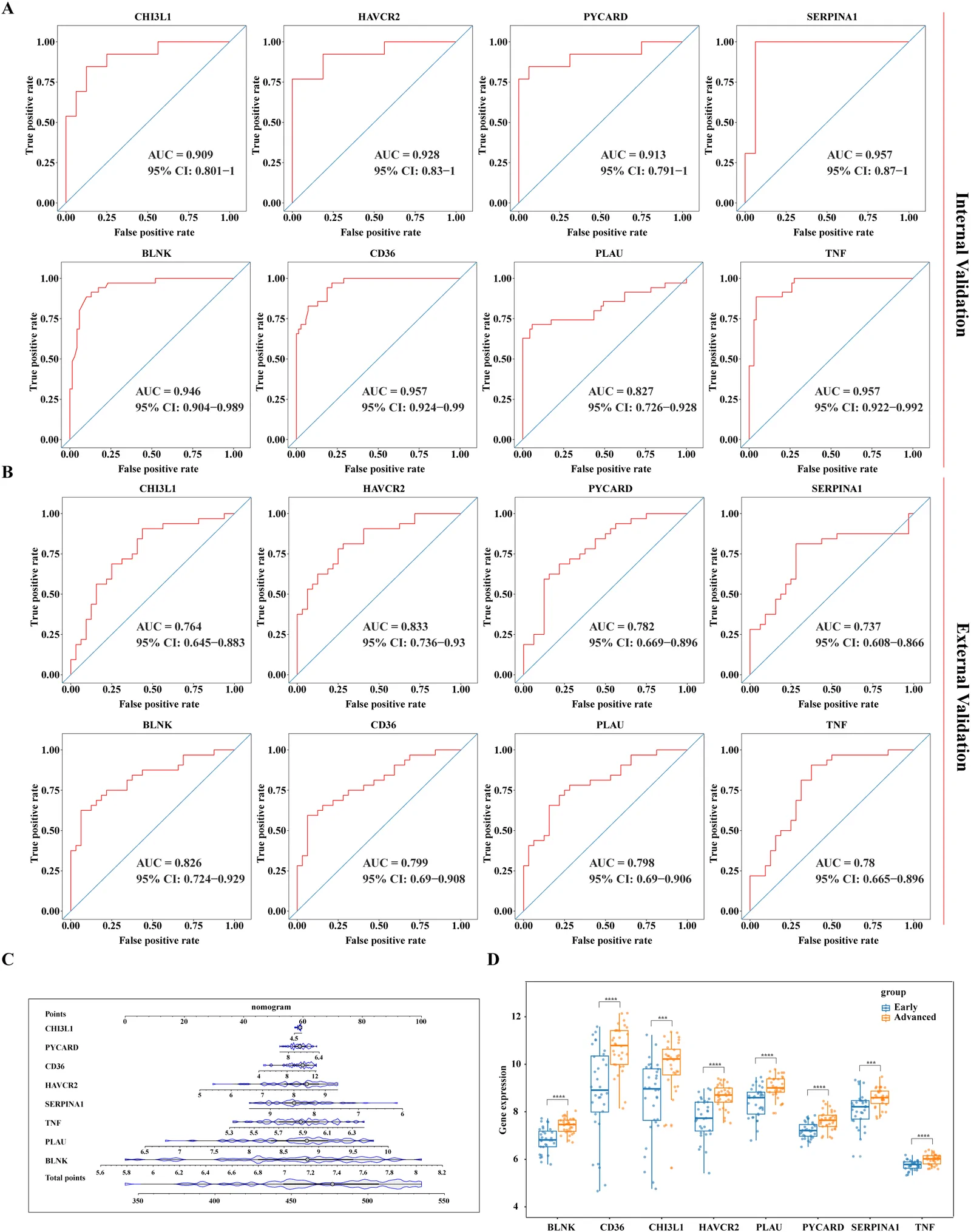
ROC-based diagnostic validation, nomogram construction, and expression comparison of eight hub genes. **(A)** Internal validation ROC curves for CHI3L1, HAVCR2, PYCARD, SERPINA1, BLNK, CD36, PLAU, and TNF. The red line indicates the ROC curve and the diagonal line indicates the no-discrimination reference. AUC and 95% CI are shown in each panel. **(B)** External validation ROC curves for the same eight hub genes in an independent cohort. **(C)** Nomogram integrating the eight hub genes. For each gene, an individual point value is assigned based on its contribution; points are summed to yield Total points, which maps to an individualized predicted risk. **(D)** Boxplots showing the expression of the eight hub genes in GSE43292, including 32 atherosclerotic carotid plaque samples and 32 patient-matched macroscopically intact adjacent carotid tissue samples. The y-axis represents processed and normalized gene-expression values obtained from the GEO Series Matrix. Dots represent individual samples, and boxes indicate the median and interquartile range. Statistical significance was assessed using two-sided Wilcoxon rank-sum tests. The reported P values were nominal and were not adjusted across the eight genes.

In the internal analyses, the eight candidate genes showed discriminatory performance for the corresponding cohort-specific comparisons: GSE100927 distinguished atherosclerotic from non-atherosclerotic tissues, whereas GSE28829 distinguished advanced from early atherosclerotic plaques (**Figure 3A**). Notably, SERPINA1, CD36, and TNF achieved the highest AUC values (AUC = 0.957 each), followed closely by BLNK, HAVCR2, PYCARD, and CHI3L1 (all AUC > 0.90). PLAU also demonstrated discriminatory performance (AUC = 0.827).

To further examine the molecular associations of these genes with atherosclerosis-related processes, we performed within-dataset correlation analyses. In GSE100927, the expression levels of BLNK, CD36, PLAU, and TNF were evaluated for correlations with genes involved in lipid uptake, foam-cell formation, and inflammatory activation, including CD36, OLR1, APOE, LPL, IL1B, and NLRP3. The same analysis was performed for CHI3L1, HAVCR2, PYCARD, and SERPINA1 within GSE28829. Several candidate genes showed significant correlations with these pathway-related genes, suggesting associations with lipid-handling, foam-cell-related, and inflammatory transcriptional programs (**Supplementary Figures S6–13**). These findings represent molecular-level associations and do not establish clinical or causal relationships.

The discriminatory performance of the candidate genes was subsequently evaluated in the independent GSE43292 cohort (**Figure 3B**). Despite expected cross-cohort differences, the genes retained discriminatory performance in this cohort. HAVCR2 (AUC = 0.833) and BLNK (AUC = 0.826) showed the highest external-cohort AUC values.

To integrate these molecular signatures into an exploratory transcriptome-based diagnostic model, we constructed an eight-gene nomogram using the GSE43292 dataset (**Figure 3C**). The model showed satisfactory discriminatory performance within this cohort, and its calibration and potential clinical utility were further evaluated in GSE43292 using bootstrap-corrected calibration analysis and decision curve analysis, respectively (**Supplementary Figure S5**). These results provide preliminary support for the diagnostic potential of the combined model; however, because model construction and these performance assessments were conducted in the same dataset, validation in larger independent clinical cohorts remains necessary.

Finally, expression profiling in the external validation cohort GSE43292 confirmed that all eight hub genes were significantly upregulated in atherosclerotic carotid plaque tissues compared with patient-matched adjacent carotid tissues (**Figure 3D**). This consistent overexpression, paired with their robust ROC performance, solidifies their biological plausibility and potential utility as non-invasive diagnostic biomarkers.

### Macrophage-dominated inflammatory microenvironment is associated with anoikis-related signatures

Given our previous findings of differential immune activation, we computationally deconvoluted the bulk transcriptomes of GSE28829 into 22 immune-cell subsets using CIBERSORT with the LM22 signature matrix. As GSE28829 comprises early and advanced atherosclerotic plaques, this analysis was used to characterize immune-cell patterns associated with plaque progression.

**Figure 4:**
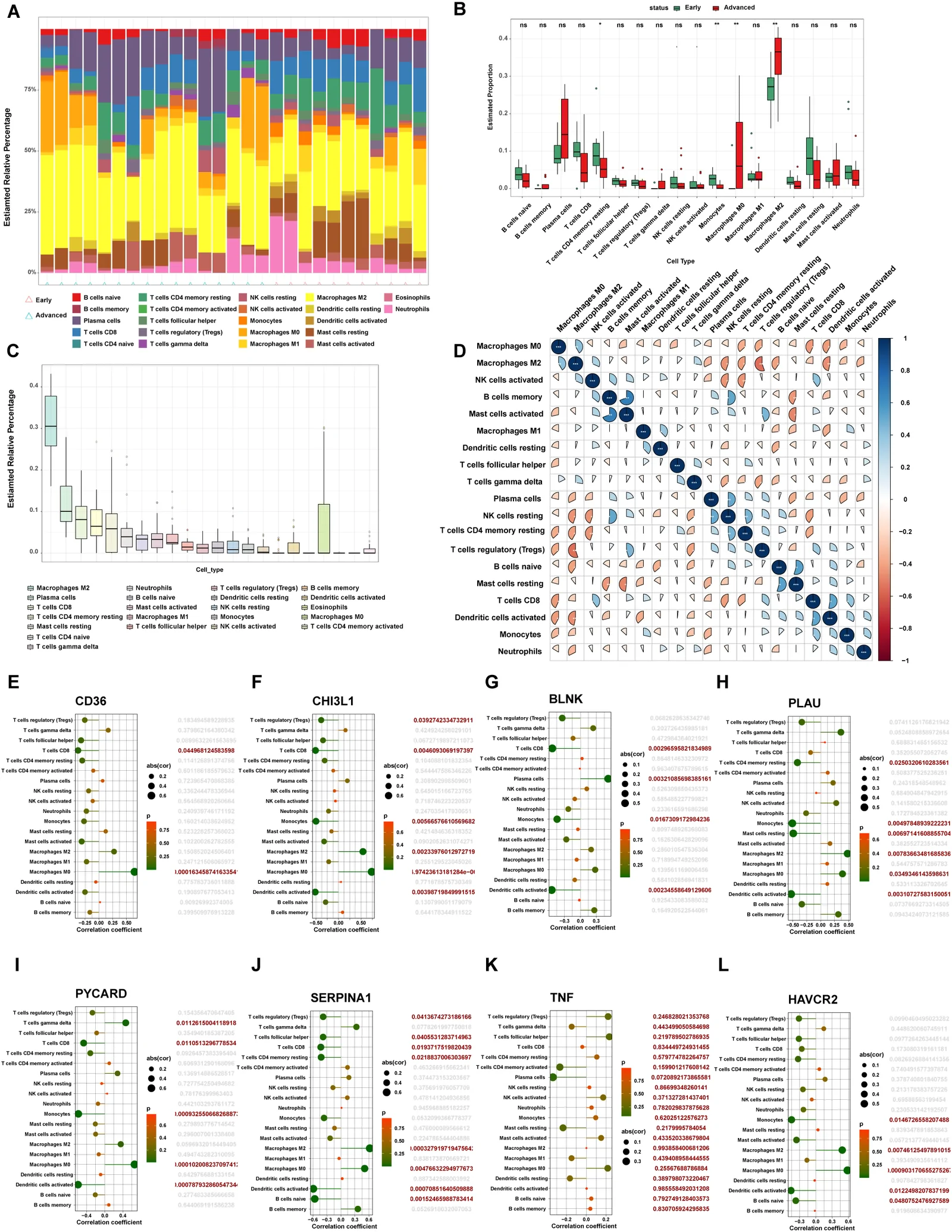
Immune infiltration landscape inferred by CIBERSORT in GSE28829 and its association with candidate genes. **(A)** Stacked bar plot showing the inferred immune composition for each sample (22 subsets). **(B)** Boxplots comparing the estimated immune-cell fractions between early and advanced atherosclerotic plaques. Statistical comparisons were performed using the two-sided Wilcoxon rank-sum test. Statistical significance is indicated above each cell subset (ns, *, **, ***). **(C)** Boxplots summarizing the overall distribution of inferred immune fractions across all samples. **(D)** Correlation matrix showing pairwise associations among the 22 inferred immune-cell fractions. **(E–L)** Dot plots showing associations between candidate-gene expression and estimated immune-cell fractions for CD36 **(E)**, CHI3L1 **(F)**, BLNK **(G)**, PLAU **(H)**, PYCARD **(I)**, SERPINA1 **(J)**, TNF **(K)**, and HAVCR2 **(L)**. Correlations were calculated using Pearson’s correlation test implemented with the cor.test function in **(R)** The x-axis denotes the Pearson correlation coefficient; dot size represents |r|, and dot color represents the nominal two-sided P value. P < 0.05 was considered statistically significant, and no multiple-comparison adjustment was applied.

The inferred immune composition showed a myeloid-enriched profile across the plaque cohort, with M2 macrophages representing the most abundant inferred immune-cell fraction, with a median estimated proportion of 30.52% (IQR, 25.77–37.78%) (**Figures 4A, C**). Comparisons between plaque stages revealed progression-associated immune remodelling (**Figure 4B**). Compared with early plaques, advanced plaques showed increased relative fractions of selected macrophage subsets and plasma cells, accompanied by reduced fractions of specific lymphocyte subsets. This myeloid-enriched pattern broadly aligns with the phagolysosomal and cytokine-signalling pathways identified in our enrichment analyses.

Pairwise correlation analysis revealed coordinated relationships among the inferred immune-cell subsets, including positive associations among selected innate immune populations and inverse associations between macrophage fractions and certain adaptive immune-cell subsets (**Figure 4D**). We subsequently examined the correlations between the eight candidate genes and the inferred immune-cell fractions (**Figures 4E–L**). CD36, CHI3L1, PLAU, PYCARD, SERPINA1, and HAVCR2 were positively correlated with M0 and/or M2 macrophages and inversely correlated with selected T-cell subsets, suggesting that their expression is associated with macrophage-enriched immune features during plaque progression.

BLNK expression was positively correlated with plasma cells, in line with its established role in B-cell-lineage signalling, whereas TNF showed broader associations across multiple immune-cell subsets. Collectively, these findings indicate that progression from early to advanced plaques is accompanied by macrophage-associated immune remodelling and that several candidate genes covary with this changing immune-cell composition.

BLNK expression was positively correlated with plasma cells, in line with its established role in B-cell-lineage signalling, whereas TNF showed broader associations across multiple immune-cell subsets. Collectively, these findings indicate that progression from early to advanced plaques is accompanied by macrophage-associated immune remodelling and that several candidate genes covary with this changing immune-cell composition.

**Figure 5:**
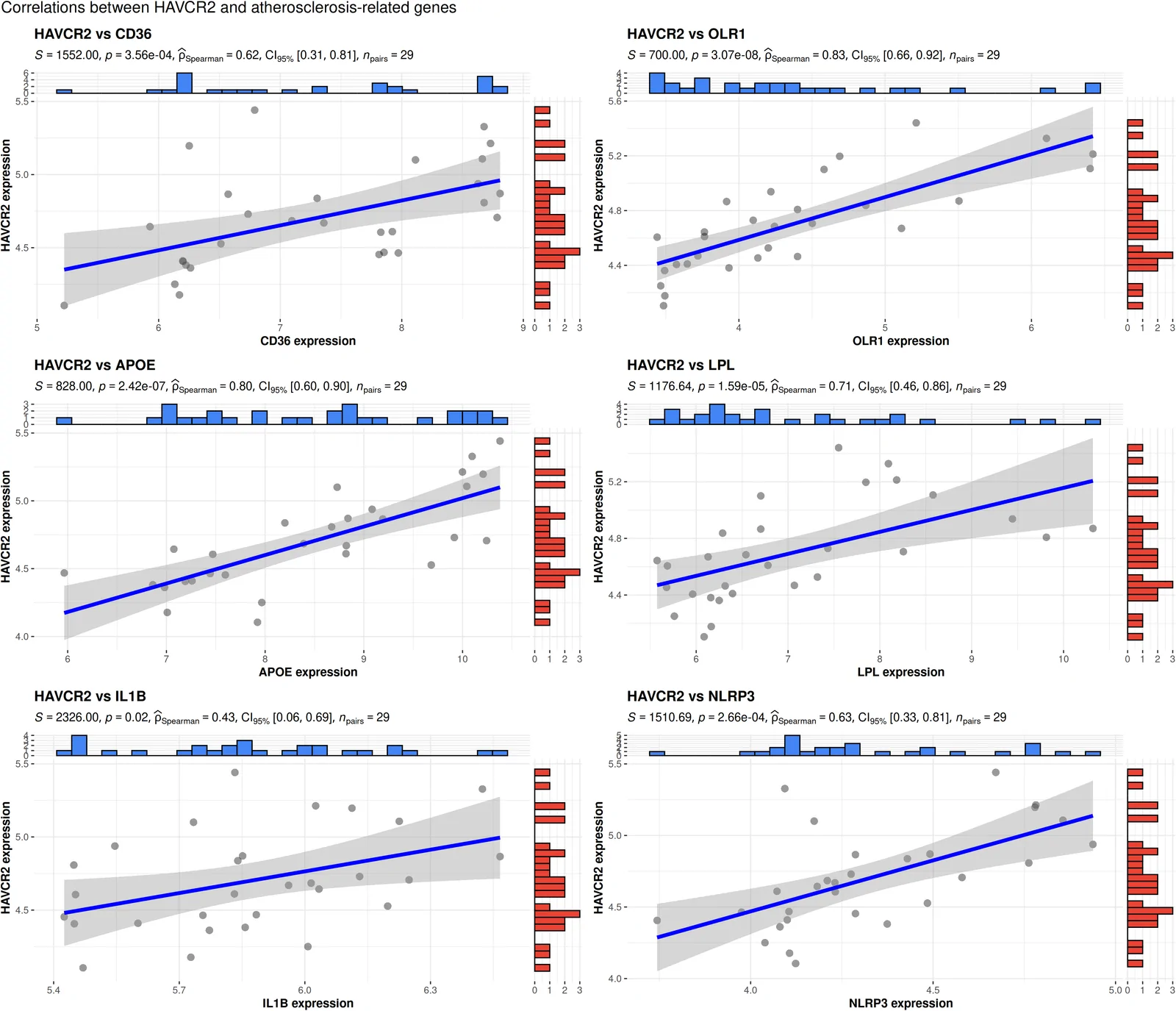
Correlations of HAVCR2 expression with lipid metabolism and inflammation-related genes in the GSE28829 dataset. Scatter plots showing the correlations between HAVCR2 expression and lipid metabolism-related genes (CD36, OLR1, APOE, and LPL) or inflammation-related genes (IL1B and NLRP3) across 29 samples from the GSE28829 dataset. HAVCR2 expression was positively correlated with CD36 (Spearman’s ρ = 0.62, *P* = 3.56 × 10^−4^), OLR1 (ρ = 0.83, *P* = 3.07 × 10^−8^), APOE (ρ = 0.80, *P* = 2.42 × 10^−7^), LPL (ρ = 0.71, *P* = 1.59 × 10^−5^), IL1B (ρ = 0.43, *P* = 0.02), and NLRP3 (ρ = 0.63, *P* = 2.66 × 10^−7^). Each point represents one sample. Blue lines indicate fitted linear regression lines, and gray shaded areas represent the corresponding 95% confidence intervals. Marginal histograms show the distributions of gene expression values. Correlations were evaluated using two-sided Spearman’s rank correlation tests.

In addition, we examined the correlations of the eight candidate genes with representative genes involved in lipid handling, foam-cell biology, and inflammatory activation within their respective discovery datasets. BLNK, CD36, PLAU, and TNF were assessed in GSE100927, whereas CHI3L1, HAVCR2, PYCARD, and SERPINA1 were assessed in GSE28829. These analyses revealed gene-specific associations with lipid-related and inflammatory transcriptional programs, providing additional molecular context without implying direct regulatory or causal relationships. Correlation results for the seven candidates other than HAVCR2 are presented in **Supplementary Figures S6–S12**, whereas the HAVCR2 correlation analysis is presented separately in **Figure 5** and described below in conjunction with its experimental evaluation.

### Macrophages serve as the primary cellular hub for anoikis in atherosclerotic lesions

**Figure 6:**
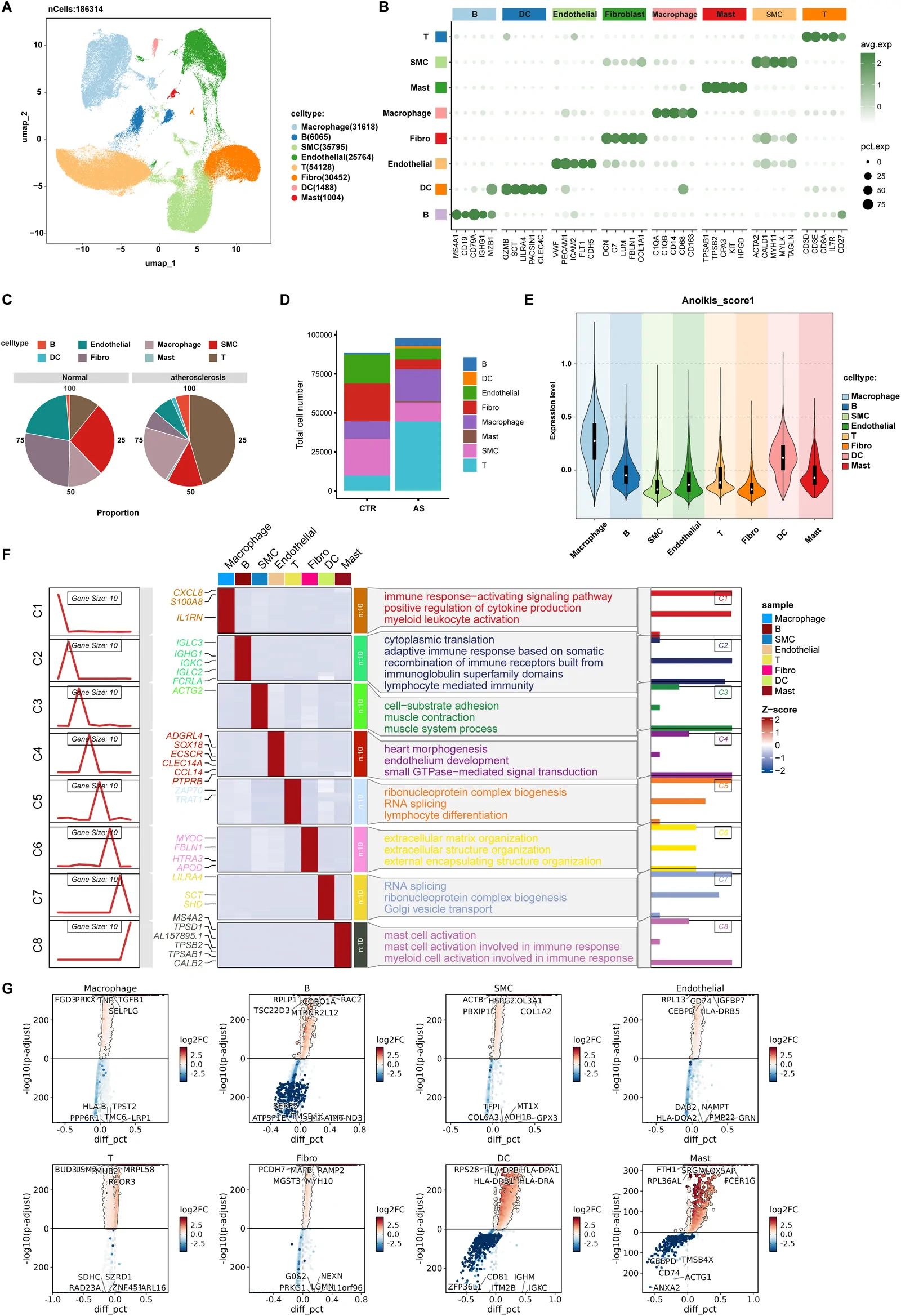
Single-cell atlas of Normal and AS samples reveals cell-type composition shifts, cell-specific anoikis activity, and lineage-stratified differential expression. **(A)** UMAP embedding of 186,314 single cells colored by annotated cell types: Macrophage, T, SMC, Endothelial, Fibro, B, DC, and Mast; numbers in parentheses indicate cell counts per type. **(B)** Dot plot of canonical marker genes across cell types. Dot color denotes average expression and dot size denotes the percentage of expressing cells. **(C)** Pie charts showing the relative proportions of the eight cell types in Normal and AS groups. **(D)** Stacked bar plot showing total cell numbers per group, partitioned by cell type. **(E)** Violin plots of Anoikis_score1 across the eight cell types; boxes indicate median and interquartile range. **(F)** Heatmap of representative marker genes organized into gene clusters C1–C8 (top genes shown per cluster; z-score scaled), accompanied by functional annotation (GO/pathway terms) and cell-type contribution summaries. **(G)** Cell type–stratified differential expression between AS and Normal shown as volcano-style scatter plots for each cell type. The x-axis represents the difference in detection percentage (diff_pct), and the y-axis represents −log10(adjusted P value); points are colored by log2 fold change and selected genes are labeled.

To elucidate the cellular heterogeneity underlying atherosclerotic transcriptional remodeling, we integrated scRNA-seq datasets from five independent cohorts, yielding a comprehensive atlas of 186,314 cells. Unsupervised clustering identified eight distinct cell lineages (**Figures 6A, B**): macrophages, T cells, smooth muscle cells (SMCs), endothelial cells (ECs), fibroblasts, B cells, dendritic cells (DCs), and mast cells. Dataset-split visualization of the Harmony-integrated atlas showed that the major annotated cell populations were generally represented across multiple datasets, although their relative contributions varied among datasets, consistent with differences in sample and vascular composition (**Supplementary Figure S13**). Descriptive analysis of cellular composition revealed higher relative proportions of immune populations, particularly T cells and macrophages, and lower relative proportions of stromal populations, including fibroblasts and ECs, in the AS group than in the CTR (normal/reference) group (**Figures 6C, D**). Sample-level analysis further demonstrated substantial variation in the relative proportions of the eight major cell types across individual samples and datasets (**Supplementary Figure S14**). This heterogeneity was consistent with differences in vascular origin, pathological context, tissue-processing and cell-capture strategies, and overall dataset composition. In particular, GSE245373 predominantly contributed immune-cell populations because it was generated from pre-sorted viable CD45+ cells. Accordingly, the pooled AS–CTR differences were interpreted as descriptive compositional patterns within the integrated dataset rather than as absolute estimates of disease-associated changes in cell abundance. These findings indicate an immune-enriched cellular composition in the AS samples, accompanied by reduced relative representation of structural cell populations.

To map the cellular distribution of the anoikis-related transcriptional signature, we calculated Anoikis-score1 at single-cell resolution (**Figure 6E**). Macrophages exhibited the highest median scores and the broadest distribution, whereas most stromal and adaptive immune-cell populations showed lower scores, indicating preferential enrichment of the eight-gene signature in macrophages. We next examined whether Anoikis-score1 was associated with downstream transcriptional programs within individual cell populations. Cell-type-stratified correlation analyses revealed associations between Anoikis-score1 and extracellular-matrix organization, disassembly, and cell–matrix adhesion programs, particularly in smooth muscle cells, fibroblasts, and endothelial cells (**Supplementary Figure S15**). Within macrophage states, the relationships between Anoikis-score1 and GMT-based pathway scores were evaluated using correlation heatmaps and scatter plots (**Supplementary Figures S16–S20**). These analyses showed state-dependent correlations of Anoikis-score1 with cholesterol-homeostasis, inflammatory, and NF-κB-related transcriptional signatures, with the strength and direction of the associations varying among macrophage subpopulations. Together, these findings connect the anoikis-related signature with macrophage inflammatory programs and stromal ECM-related processes beyond differences in cell identity. Pathway enrichment and cell-type-stratified differential-expression analyses further characterized lineage-specific transcriptional alterations across the plaque microenvironment (**Figures 6F, G**). In macrophages, classical inflammatory mediators (TNF, TGFB1) were significantly upregulated. Concurrently, SMCs and fibroblasts showed aberrant ECM and cytoskeletal signatures, validating our bulk transcriptomic observations of structural and contractile loss.

While macrophages exhibited the strongest anoikis-related transcriptional signature, disease-associated transcriptional heterogeneity was also observed within the adaptive immune compartment. Subclustering of 54,128 T cells identified 12 transcriptional states, including cytotoxic, interferon-responsive, helper, regulatory, and memory-associated populations. Functional enrichment of subset-specific gene signatures indicated the involvement of antigen-processing and presentation, MHC class II complex assembly, interferon/antiviral responses, and cytotoxic immune programs across specific T-cell states (**Supplementary Figure S21**). Cytotoxic_T and MAIT cells exhibited cytotoxic-effector and innate-like T-cell features, represented by TRGV7, SIGLEC7, KIR3DL2, KLRD1, and NKG7. The IFN_response population was characterized by interferon-stimulated genes, including IFI44L, RSAD2, IFIT1, IFIT2, IFIT3, OAS1, and MX1. MHC class II- and antigen-presentation-associated genes, including HLA-DRA, HLA-DPA1, HLA-DRB5, and HLA-DQA2, were observed among the differentially expressed genes in Th1 cells. Other subsets displayed heterogeneous combinations of T-cell receptor, activation, effector, stress-response, and metabolic features. Overall, these profiles were broadly consistent with the assigned T-cell identities, although several subsets shared transcriptional features and were not completely separable at the transcriptomic level (**Supplementary Figure S22**). Finally, regulon network analysis confirmed that this T-cell polarization is orchestrated by highly specialized TF circuitries—ranging from *TCF7*-driven naive maintenance to *NFATC3*-mediated effector activation and *NFE2L2* (*NRF2*)-linked oxidative stress adaptation (**Supplementary Figure S24**). Collectively, these single-cell analyses associate anoikis-related transcriptional signatures predominantly with macrophage states and immune-stromal remodelling in atherosclerotic lesions, alongside state-specific transcriptional changes in adaptive immune cells.

### TF-driven expansion of foam and inflammatory macrophages dictates lesional anoikis

**Figure 7:**
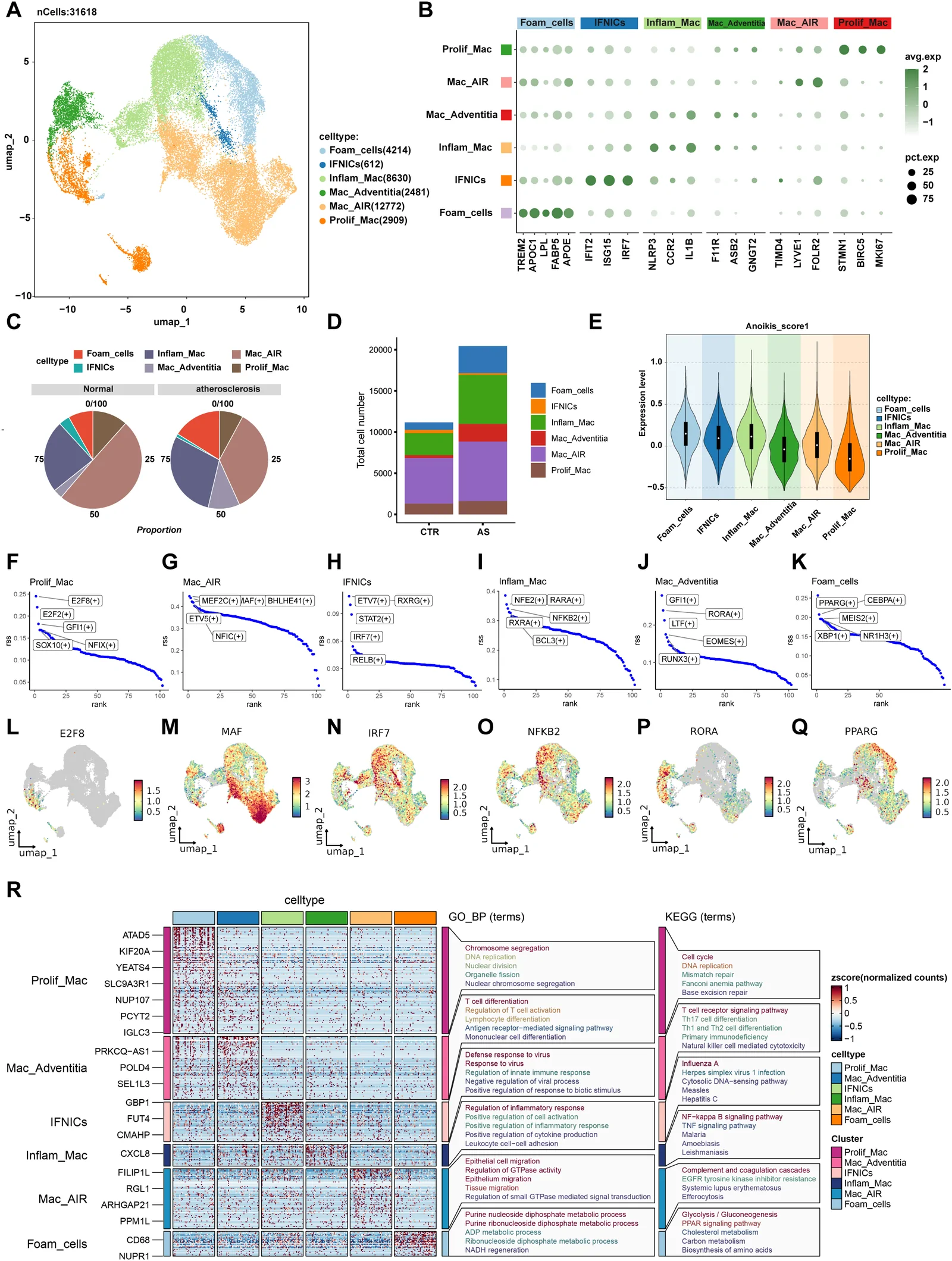
Macrophage re-clustering defines six lesion-associated states, their compositional shifts, anoikis activity, regulatory programs, and functional enrichment. **(A)** UMAP of 31,618 macrophage-lineage cells. Cell numbers for each state are shown in the legend. **(B)** Dot plot of representative marker genes across macrophage states. Dot color indicates average expression (avg.exp) and dot size indicates the fraction of expressing cells (pct.exp). **(C)** Pie charts showing the relative proportions of macrophage states in Normal and AS groups. **(D)** Stacked bar plot showing absolute cell numbers per group partitioned by macrophage state. **(E)** Violin plots of Anoikis_score1 across the six macrophage states; boxes denote median and interquartile range. **(F–K)** Regulon specificity score (RSS) curves showing state-specific TF regulons for Prolif_Mac **(F)**, Mac_AIR **(G)**, IFNICs **(H)**, Inflam_Mac **(I)**, Mac_Adventitia **(J)**, and Foam_cells **(K)**; top regulons are labeled. **(L–Q)** UMAP feature plots of representative TFs/regulon activities: E2F8 **(L)**, MAF **(M)**, IRF7 **(N)**, NFKB2 **(O)**, RORA **(P)**, and PPARG **(Q)**. **(R)** Heatmap of selected state-associated genes (z-score of normalized counts) across macrophage states (columns annotated by state). Right panels show representative enriched GO Biological Process (GO_BP) and KEGG terms for each state.

To decode the precise macrophage heterogeneity driving lesional inflammation and anoikis, we subclustered 31,618 macrophage-lineage cells into six transcriptionally distinct states: Foam_cells, interferon-stimulated (IFNICs), inflammatory (Inflam_Mac), Mac_Adventitia, active immune response (Mac_AIR), and Prolif_Mac (**Figures 7A, B**). Compositional analysis revealed a disease-associated shift in macrophage composition, characterized by a relative expansion of Foam_cells and Inflam_Mac subpopulations in AS samples (**Figures 7C, D**).

Crucially, mapping the Anoikis_score1 across these subpopulations demonstrated that anoikis activity was intensely enriched in Foam_cells and Inflam_Mac, while remaining lowest in Prolif_Mac (**Figure 7E**). This is consistent with that anoikis-linked transcriptional programs are preferentially coupled to terminally differentiated, stress-adapted foam and inflammatory phenotypes, rather than actively proliferating states.

To elucidate the upstream architects of these distinct phenotypic states, we evaluated TF activity alongside state-stratified differential expression (**Figures 7F–R**, **Supplementary Figure S23**). This integrative analysis confirmed that macrophage heterogeneity is governed by highly specialized TF circuitries driving discrete pathogenic signatures. Specifically, the lipid-metabolic core of Foam_cells was heavily enriched for cholesterol metabolism and PPAR signaling, directly anchored by the *PPARG*, *NR1H3*, and *XBP1* regulons. These master regulators strictly controlled lipid-handling and vesicle/lysosomal transcripts (e.g., *TREM2*, *CD36*, *TYROBP*). Conversely, the Inflam_Mac state was associated with increased NFKB2 regulon activity and the expression of inflammatory mediators and TNF/NF-κB-related genes (e.g., NLRP3, IL1B, NFKBIA). Furthermore, IFNICs exhibited a robust IRF7-driven antiviral signature (ISG15, IFI44L), while Prolif_Macs were tightly controlled by E2F8 and classic cell-cycle regulators (MKI67). Collectively, these high-resolution regulon maps and functional profiles mechanistically confirm that the AS-specific expansion of foam and inflammatory macrophages—orchestrated by distinct lipid and inflammatory TF networks—serves as the primary cellular engine for lesional anoikis and immunometabolic pathology.

### A regulatory handoff from NFKB2 to PPARG controls stage-specific anoikis activation during macrophage foam cell formation

**Figure 8:**
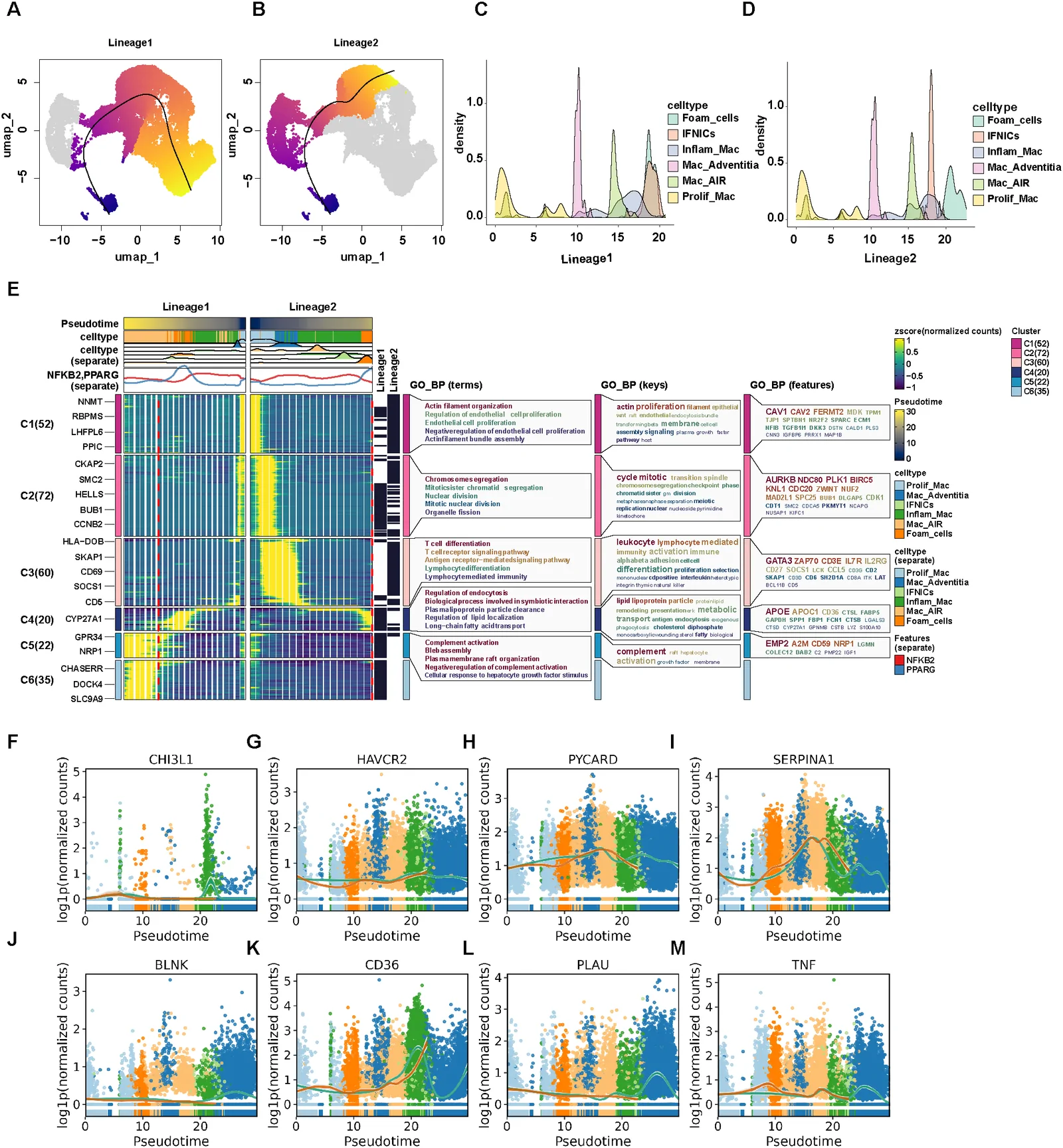
Pseudotime trajectories of macrophage states reveal lineage-specific dynamics of transcriptional programs and hub genes. **(A)** UMAP showing Lineage1 trajectory overlaid on macrophage embedding; cells are colored by pseudotime progression and the inferred trajectory is indicated by the black curve (cells not assigned to this lineage are shown in gray). **(B)** UMAP showing Lineage2 trajectory. **(C)** Density distributions of pseudotime along Lineage1 for each macrophage state. **(D)** Density distributions of pseudotime along Lineage2 for each macrophage state. **(E)** Heatmap of pseudotime-dependent genes across Lineage1 and Lineage2 (z-score of normalized counts), grouped into six gene clusters (C1–C6). Top tracks indicate pseudotime, macrophage state annotations, and smoothed expression trends of NFKB2 and PPARG along each lineage. Representative enriched GO_BP terms and key feature genes are shown for each cluster. **(F–M)** Scatter plots showing expression of hub genes along pseudotime (log1p normalized counts): CHI3L1 **(F)**, HAVCR2 **(G)**, PYCARD **(H)**, SERPINA1 **(I)**, BLNK **(J)**, CD36 **(K)**, PLAU **(L)**, and TNF **(M)**. Points represent single cells colored by macrophage state; smoothed curves indicate lineage-specific expression trends.

To characterize potential relationships among the identified macrophage subpopulations, we performed computational trajectory inference and pseudotime analysis using Slingshot. Two related state-transition paths were inferred from the Prolif_Mac population toward distinct macrophage states (**Figures 8A, B**). The trajectories shared an initial region but subsequently traversed different regions of the macrophage-state landscape, suggesting that these populations may occupy multiple coordinated transcriptional paths rather than a single linear continuum. Because the trajectories were inferred from transcriptomic profiles, they should be interpreted as exploratory state relationships rather than definitive evidence of irreversible lineage differentiation or biological bifurcation.

Pseudotime density distributions further demonstrated that the macrophage states occupied different, but partially overlapping, positions along the two inferred lineages (**Figures 8C, D**). Prolif_Mac was concentrated near the beginning of both trajectories, consistent with its specified role as the starting state. Mac_Adventitia and Mac_AIR were primarily distributed across intermediate pseudotime regions, whereas Inflam_Mac, IFNICs, and Foam_cells were more frequently represented in intermediate-to-late regions, with some differences between the two lineages. These patterns are consistent with a transcriptional continuum from proliferative states toward inflammatory and lipid-associated macrophage states, although the directionality and biological sequence of these relationships require experimental validation.

Pseudotime-dependent module analysis identified coordinated changes in transcriptional and transcription factor-associated programs along the inferred lineages (**Figure 8E**). Early pseudotime regions were enriched for cell-cycle, chromosome-segregation, and cytoskeletal-remodeling programs. Intermediate regions showed increased expression of immune-associated modules involving T-cell interaction, endocytosis, and inflammatory functions. A lipid-handling and endocytic module containing APOE, APOC1, CD36, CTSL, and FABP5 became prominent across intermediate-to-late regions. NFKB2-associated activity was most evident in an intermediate region, whereas PPARG-associated activity increased toward later pseudotime, particularly along Lineage 2. Together, these patterns are consistent with a relative shift from inflammation-associated toward lipid-processing transcriptional states. However, they represent transcriptome-derived associations and do not establish a causal NFKB2-to-PPARG regulatory switch.

The eight ARG hub genes displayed heterogeneous pseudotime-associated expression patterns (**Figures 8F–M**). CHI3L1 showed a pronounced increase within a later-pseudotime cell population, whereas HAVCR2 exhibited a modest increase across intermediate-to-late regions. PYCARD and SERPINA1 showed transient elevations in intermediate regions, while CD36 increased prominently toward later pseudotime, consistent with its association with lipid-handling macrophage states. PLAU and TNF displayed state-dependent variation, whereas BLNK remained comparatively low across most of the trajectory. Overall, these findings provide an associative trajectory-based framework linking macrophage states, functional gene modules, transcription factor-associated patterns, and ARG hub-gene expression. They do not establish the direction, irreversibility, or causal biological significance of the inferred transitions.

### HAVCR2 is positively associated with lipid-handling and inflammatory gene programs

To further contextualize the molecular associations of HAVCR2, we examined its expression correlations with atherosclerosis-related genes in the independent GSE28829 cohort. HAVCR2 expression showed positive correlations with genes involved in lipid uptake and foam-cell biology, including CD36 (Spearman’s ρ = 0.62), OLR1 (ρ = 0.83), APOE (ρ = 0.80), and LPL (ρ = 0.71). HAVCR2 was also positively correlated with the inflammatory genes IL1B (ρ = 0.43) and NLRP3 (ρ = 0.63) (all n = 29; **Figure 5**). These associations link HAVCR2 expression with lipid-handling and inflammatory transcriptional programs in carotid plaque tissues and provided additional molecular context for the subsequent functional experiments.

### Macrophage-specific HAVCR2 upregulation drives lipid-induced atherosclerotic plaque progression

**Figure 9:**
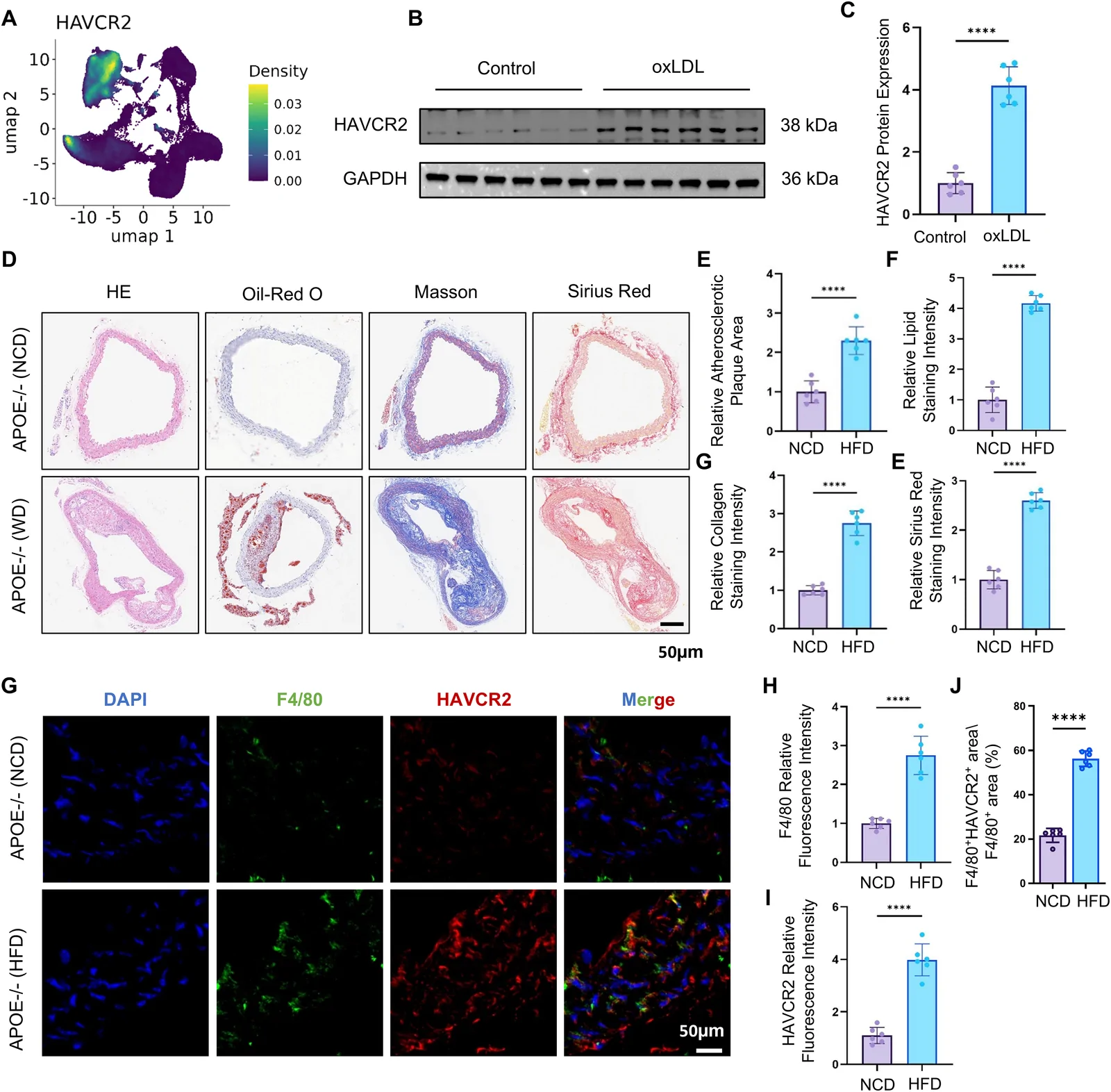
Validation of HAVCR2 expression in atherosclerosis using single-cell localization, oxLDL stimulation, and ApoE^−/−^ mouse lesions. **(A)** UMAP density plot showing the distribution of HAVCR2 expression at single-cell level. **(B)** Western blot analysis of HAVCR2 protein expression in untreated and oxLDL-treated THP-1-derived macrophages, with GAPDH as the loading control. **(C)** Densitometric quantification of HAVCR2 expression in **(B)**. Six independently cultured dishes were analyzed per condition (n = 6 independent culture-level experimental replicates per group). **(D)** Representative aortic root sections from ApoE−/− mice fed NCD or HFD, stained with H&E, Oil Red O, Masson’s trichrome, and Sirius Red. Scale bar, 200 μm **(E)** Quantification of relative plaque area, lipid-staining intensity, and collagen/fibrosis-associated staining intensity in the NCD and HFD groups. Data were obtained from one independent *in vivo* experiment with six ApoE−/− mice per group (n = 6 independent biological replicates per group). **(F)** Representative immunofluorescence images of aortic root lesions showing DAPI, F4/80, HAVCR2, and merged signals in the NCD and HFD groups. **(G)** Quantification of F4/80 and HAVCR2 fluorescence intensity in **(F)**, with six individual mice analyzed per group (n = 6 biological replicates per group). Scale bar, 50 μmData are presented as mean ± SD. Statistical significance was assessed using an unpaired two-sided Student’s t-test. ****P < 0.0001.

To substantiate the transcriptomic findings, HAVCR2 was further examined across single-cell, *in vitro*, and *in vivo* models. At single-cell resolution, HAVCR2 expression was preferentially detected in macrophage populations, particularly in disease-associated macrophage states, rather than being uniformly distributed across all plaque cell types (**Figure 9A**). This distribution was consistent with the association of HAVCR2 with macrophage-enriched immune features in the bulk analyses, although it represents an expression-localization association rather than evidence of a unique or causal role.

To assess the response of HAVCR2 to atherogenic lipid stress, THP-1-derived macrophages were exposed to oxLDL. Western blot analysis showed that oxLDL stimulation increased HAVCR2 protein expression compared with untreated controls (**Figures 9B, C**). The *in vivo* relevance of these observations was further examined in ApoE−/− mice. Compared with the NCD group, HFD feeding markedly increased aortic-root plaque burden, lipid deposition, and collagen/fibrosis-associated staining (**Figures 9D–F**). Immunofluorescence analysis showed increased F4/80 and HAVCR2 signals in HFD-induced lesions, together with greater overlap between the two signals (**Figures 9G–J**).

**Figure 10:**
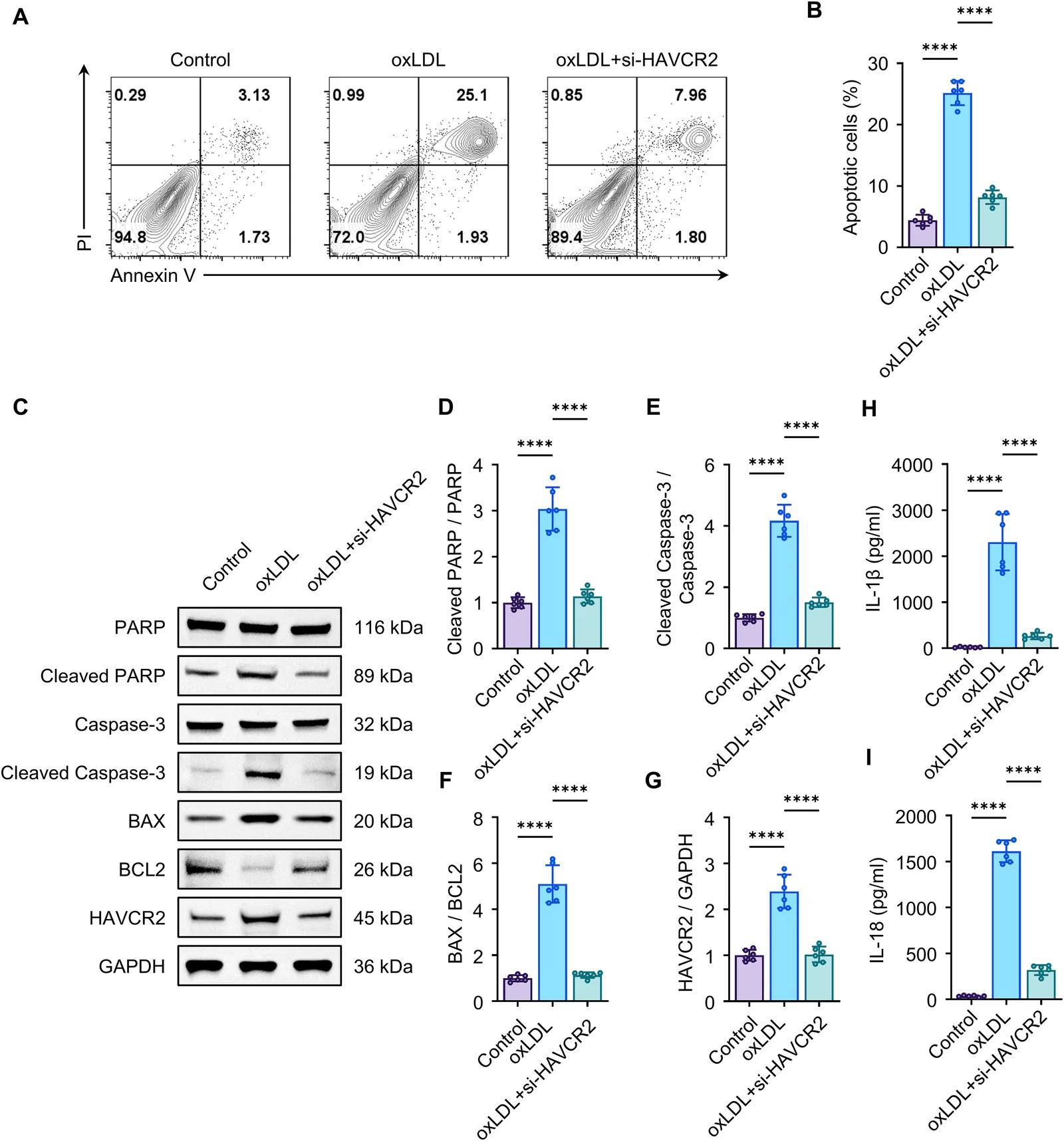
HAVCR2 silencing attenuates oxLDL-induced apoptosis and inflammatory responses in THP-1-derived macrophages. **(A)** Representative Annexin V–FITC/propidium iodide **(PI)** flow-cytometry plots of untreated control cells, oxLDL-treated cells, and oxLDL-treated cells transfected with HAVCR2-targeting siRNA (oxLDL + si-HAVCR2). The percentages of cells within each quadrant are indicated. **(B)** Quantification of the total apoptotic-cell fraction based on Annexin V staining. **(C)** Representative Western blots showing PARP, cleaved PARP, caspase-3, cleaved caspase-3, BAX, BCL2, HAVCR2, and GAPDH protein expression in the three experimental groups. GAPDH was used as the loading control. **(D–G)** Densitometric quantification of the cleaved PARP/PARP ratio **(D)**, cleaved caspase-3/caspase-3 ratio **(E)**, BAX/BCL2 ratio **(F)**, and HAVCR2/GAPDH ratio **(G)**. **(H, I)** Concentrations of IL-1β **(H)** and IL-18 **(I)** in culture supernatants. Bars represent the mean ± SD, and dots represent independent culture-level experimental replicates. Statistical significance was assessed using [insert statistical test]. ***P < 0.001; ****P < 0.0001.

We next examined the functional consequences of HAVCR2 suppression under oxLDL stimulation. Flow-cytometric analysis showed that oxLDL increased the proportion of apoptotic macrophages, whereas HAVCR2 silencing attenuated this response (**Figures 10A, B**). Consistently, oxLDL increased cleaved PARP, cleaved caspase-3, and the BAX/BCL2 ratio, while HAVCR2 silencing reduced these apoptosis-associated changes (**Figures 10C–F**). The reduction in HAVCR2 protein expression confirmed the effectiveness of the intervention (**Figure 10G**). ELISA measurements further showed that HAVCR2 silencing reduced oxLDL-induced secretion of the inflammatory cytokines IL-1β and IL-18 (**Figures 10H, I**). These findings indicate that HAVCR2 expression is associated with oxLDL-induced apoptotic and inflammatory responses in THP-1-derived macrophages.

These findings support the association of HAVCR2 expression with macrophage-enriched atherosclerotic lesions and lesion-associated pathological remodeling.

## Discussion

AS is a chronic inflammatory arterial disease driven by persistent lipid-induced injury and maladaptive immune responses, which collectively reshape the vessel wall and precipitate plaque instability (19–21). Recent large-scale single-cell transcriptomic studies have shifted the paradigm of plaque biology, demonstrating that lesional microenvironments are organized around highly specialized, discrete immune and vascular cell states rather than uniform lineages (22, 23). Despite these advances, the precise mechanisms by which adhesion-dependent survival programs—specifically anoikis—interface with persistent immune cell retention within the necrotic plaque niche remain insufficiently characterized (24).

In this study, we identified an anoikis-related transcriptional signature associated with atherosclerosis and plaque progression, featuring *HAVCR2*, *CD36*, *TNF*, *CHI3L1*, *PLAU*, *PYCARD*, *SERPINA1*, and *BLNK*. Rather than serving as isolated biomarkers, these candidate genes were associated with lipid uptake-inflammation coupling, inflammasome-mediated cell-death wiring, and matrix-adhesion signaling (25). Together, these programs may contribute to lesional immune retention and survival under matrix-disrupted conditions.

Among the identified candidate genes, HAVCR2 was prioritized for further investigation based on its identification in the plaque-progression-associated GSE28829 cohort, its macrophage-associated expression pattern in the integrated single-cell analysis, its expression-level response to oxLDL stimulation and in ApoE−/− atherosclerotic lesions, and supporting evidence from the existing literature (17). Our findings establish a previously unrecognized macrophage-centric role for HAVCR2 in atherosclerosis, which is mechanistically and conceptually distinct from the prior demonstration of its function on T cells. While Foks et al. showed that antibody-mediated blockade of HAVCR2 accelerates lesion growth via T-cell modulation, our study provides the first evidence, to our knowledge (26), that HAVCR2 expression per se in lesional macrophages is dynamically regulated by atherogenic stimuli. Specifically, we demonstrate that HAVCR2 is markedly upregulated in progressive human atheroma, correlates positively with lipid metabolism and inflammatory gene signatures, and is directly induced by oxLDL in macrophages. Furthermore, its genetic ablation in macrophages ameliorates apoptosis and inflammatory markers. Collectively, these data position HAVCR2 not merely as a T-cell checkpoint, but as a macrophage-intrinsic stress-responsive gene that actively tracks with lesion progression, thereby extending the existing paradigm from a causal immune modifier to a correlative and potentially therapeutic disease biomarker in the myeloid compartment (26). Crucially, our single-cell trajectory analyses revealed that *HAVCR2* is highly enriched in terminally differentiated foam-cell and inflammatory macrophage states. This progressive upregulation suggested that HAVCR2 acts either as an adaptive “brake” attempting to restrain chronic inflammation, or as a distinct survival marker for macrophages persisting under severe lipid and ECM stress (27). The recent identification of HAVCR2 co-expression patterns in arteriosclerotic T-cell compartments further reinforces the likelihood of a microenvironment-wide checkpoint remodeling in AS (28).

Our data implicate *CD36* as the mechanistic engine linking lipid handling to macrophage persistence. Beyond functioning as a canonical scavenger receptor for oxLDL, CD36 serves as a signaling platform that drives pro-atherogenic intracellular cascades and mitochondrial metabolic reprogramming (29, 30). This metabolic rewiring stabilizes a lesional macrophage state that is simultaneously highly inflammatory and resistant to apoptotic clearance (31, 32). Furthermore, the inclusion of *PYCARD* (ASC) indicates that inflammasome competence is deeply embedded within the anoikis signature. Consistent with the CANTOS trial—which validated the causal role of IL-1β in cardiovascular events (8)—and studies utilizing the NLRP3 inhibitor MCC950 (33), our observation that *PYCARD* tightly co-varies with inflammatory macrophage states suggests that inflammasome-driven cytokine amplification is integral to adhesion-stress tolerance and lesional persistence.

Because anoikis is fundamentally triggered by the loss of appropriate cell-matrix anchorage, plaque ECM remodeling provides a natural mechanistic bridge between inflammation and survival (24, 34). Prior studies demonstrate that integrin signaling regulates inflammatory activation and fibronectin deposition in AS (35, 36). Within our hub signature, genes such as *SERPINA1* and *BLNK* further contextualize this advanced microenvironment. Genetic variants in *SERPINA1* are strongly linked to large-artery stroke risk (37, 38), reflecting a disrupted protease-balance milieu typical of advanced AS. Concurrently, *BLNK*, classically associated with B-cell receptor signaling, has recently been shown to modulate macrophage cytoskeletal dynamics and migration (39), mechanistically resonating with our finding that the candidate-gene signature is associated with immune trafficking and spatial retention.

A strength of this study is the integration of bulk transcriptomic analysis, single-cell state localization, trajectory inference, molecular correlation analysis, and *in vitro* and *in vivo* validation. The positive correlations between HAVCR2 and genes involved in lipid handling and inflammatory activation provide additional molecular context, while HAVCR2 loss-of-function experiments demonstrate its association with oxLDL-induced apoptotic and inflammatory responses in THP-1-derived macrophages. Several limitations should be acknowledged. Although HAVCR2 silencing attenuated oxLDL-induced apoptosis, this lipid-stress model does not fully reproduce classical anoikis directly triggered by loss of cell–ECM attachment. Further validation using low-adhesion culture systems is therefore warranted. Moreover, the current molecular and *in vitro* evidence does not establish the *in vivo* role of HAVCR2 in plaque progression, which will require further genetic validation. Spatial transcriptomic studies may also help define the distribution of anoikis-related signatures relative to macrophage states and the matrix-disrupted necrotic core.

Furthermore, apparently different patterns of T-cell composition were observed between the bulk transcriptomic immune-deconvolution and integrated single-cell analyses. These findings are not directly comparable because the two analyses addressed different biological contrasts and used different analytical frameworks. The GSE28829 CIBERSORT analysis compared early with advanced atherosclerotic plaques, whereas the integrated scRNA-seq analysis descriptively compared a heterogeneous AS group with a normal/reference group comprising non-atherosclerotic donor tissues and lesion-adjacent reference tissues. In addition, CIBERSORT estimates relative fractions of predefined immune-cell categories from bulk expression profiles, whereas scRNA-seq measures the relative representation of captured and quality-controlled cells and resolves more heterogeneous T-cell states. Previous single-cell studies have identified T cells as a major immune population in human atherosclerotic plaques and reported substantial heterogeneity in their activation and differentiation states (22, 40). One possible interpretation is that the relative distribution of selected T-cell subsets changes during progression from early to advanced plaques as macrophage and other myeloid compartments are remodelled, even though T cells may remain more highly represented in atherosclerotic than in reference vascular tissues. Accordingly, the apparently opposite patterns in **Figures 4** and **6** likely reflect differences in biological contrast, relative-composition measurements, cellular resolution, and dataset composition rather than directly contradictory estimates of the same disease-associated change. An additional limitation concerns the use of CIBERSORT with the LM22 reference matrix. LM22 represents predefined, relatively broad immune-cell categories and does not explicitly include atherosclerosis-specific foam cells or the diverse functional macrophage states present within plaques. Consequently, disease-associated macrophages may be assigned to the most transcriptionally similar LM22 reference populations, potentially biasing the estimated cell fractions. To provide complementary, higher-resolution information, our single-cell analysis resolved macrophages into six transcriptionally defined states: Foam_cells, IFNICs, Inflam_Mac, Mac_Adventitia, Mac_AIR, and Prolif_Mac. Therefore, the CIBERSORT results should be interpreted as relative trends in broad immune-cell composition rather than precise estimates of specific plaque-associated macrophage subpopulations.

However, while the GSE100927 dataset includes samples from carotid, femoral, and infra-popliteal arteries—which may introduce heterogeneity due to vascular origin differences—our pooling strategy was designed to capture core transcriptional features common to atherosclerosis across vascular beds, an approach widely used in the field (41). Similarly, GSE28829 contains early and advanced atherosclerotic plaques rather than non-atherosclerotic controls, and was therefore used to identify progression-associated rather than disease-versus-health transcriptional changes (41, 42). Nevertheless, this study has certain limitations. Within-dataset correlations linked the eight candidate genes to lipid-handling, foam-cell, and inflammatory transcriptional programs; however, these molecular associations do not establish causality or clinical relevance. Because the GEO cohorts lacked complete and harmonized clinical characteristics and longitudinal cardiovascular outcomes, gene–phenotype and prognostic associations could not be reliably assessed. Prospective cohorts with standardized clinical data and follow-up are therefore needed to establish the clinical utility of these candidates. The integrated scRNA-seq analysis also has several limitations. The included public datasets differed in vascular origin, donor characteristics, sampling strategy, cell-selection procedure, and experimental protocol. Although Harmony reduced dataset-associated separation, it cannot eliminate biological heterogeneity related to these factors. Moreover, the normal/reference and atherosclerotic samples differed partly in anatomical origin, preventing the independent effects of disease status and vascular location from being fully distinguished. One dataset was also generated from pre-sorted viable CD45+ cells and therefore contributed only immune populations, limiting quantitative comparisons of cell-type proportions across datasets. Accordingly, the integrated results should be interpreted as associations within the pooled datasets rather than disease-specific or causal effects. Validation in larger cohorts containing anatomically matched normal and atherosclerotic tissues is warranted.

## Conclusion

In summary, we define a conserved, macrophage-anchored anoikis regulatory program in AS that orchestrates lipid handling (CD36), inflammatory amplification (TNF, PYCARD), and matrix-adhesion survival signaling. The highly specific induction and lesional localization of HAVCR2 highlight it as a prioritized diagnostic biomarker and a promising therapeutic node. These findings provide a robust mechanistic framework for future investigations into how adhesion-stress adaptation shapes immune-cell persistence and drives atherosclerotic plaque progression.

## Data Availability

The original contributions presented in the study are included in the article/**Supplementary Material**. Further inquiries can be directed to the corresponding authors.

## References

[1] Roth GA Mensah GA Johnson CO Addolorato G Ammirati E Baddour LM . Global burden of cardiovascular diseases and risk factors, 1990-2019: Update from the GBD 2019 study. J Am Coll Cardiol. (2020) 76:2982–3021. doi: 10.1016/j.jacc.2020.11.010 33309175 PMC7755038

[2] Libby P Buring JE Badimon L Hansson GK Deanfield J Bittencourt MS . Atherosclerosis. Nat Rev Dis Primers. (2019) 5:56. doi: 10.1016/b978-1-4377-2930-6.00008-2 31420554

[3] Davignon J Ganz P . Role of endothelial dysfunction in atherosclerosis. Circulation. (2004) 109:III27–32. doi: 10.1161/01.cir.0000131515.03336.f8 15198963

[4] Tabas I Williams KJ Boren J . Subendothelial lipoprotein retention as the initiating process in atherosclerosis: Update and therapeutic implications. Circulation. (2007) 116:1832–44. doi: 10.1161/CIRCULATIONAHA.106.676890 17938300

[5] Bennett MR Sinha S Owens GK . Vascular smooth muscle cells in atherosclerosis. Circ Res. (2016) 118:692–702. doi: 10.1161/circresaha.115.306361 26892967 PMC4762053

[6] Basatemur GL Jorgensen HF Clarke MCH Bennett MR Mallat Z . Vascular smooth muscle cells in atherosclerosis. Nat Rev Cardiol. (2019) 16:727–44. doi: 10.1038/s41569-019-0227-9 31243391

[7] Hansson GK Hermansson A . The immune system in atherosclerosis. Nat Immunol. (2011) 12:204–12. doi: 10.1038/ni.2001 21321594

[8] Ridker PM Everett BM Thuren T MacFadyen JG Chang WH Ballantyne C . Antiinflammatory therapy with canakinumab for atherosclerotic disease. N Engl J Med. (2017) 377:1119–31. doi: 10.1056/nejmoa1707914 28845751

[9] Clarke MC Figg N Maguire JJ Davenport AP Goddard M Littlewood TD . Apoptosis of vascular smooth muscle cells induces features of plaque vulnerability in atherosclerosis. Nat Med. (2006) 12:1075–80. doi: 10.1038/nm1459 16892061

[10] Michel JB . Anoikis in the cardiovascular system: Known and unknown extracellular mediators. Arterioscler Thromb Vasc Biol. (2003) 23:2146–54. doi: 10.1161/01.ATV.0000099882.52647.E4 14551156

[11] Escate R Padro T Badimon L . LDL accelerates monocyte to macrophage differentiation: Effects on adhesion and anoikis. Atherosclerosis. (2016) 246:177–86. doi: 10.1016/j.atherosclerosis.2016.01.002 26800307

[12] SChadt EE . Molecular networks as sensors and drivers of common human diseases. Nature. (2009) 461:218–23. doi: 10.1038/nature08454 19741703

[13] Cochain C Vafadarnejad E Arampatzi P Pelisek J Winkels H Ley K . Single-cell RNA-Seq reveals the transcriptional landscape and heterogeneity of aortic macrophages in murine atherosclerosis. Circ Res. (2018) 122:1661–74. doi: 10.1161/circresaha.117.312509 29545365

[14] Wirka RC Wagh D Paik DT Pjanic M Nguyen T Miller CL . Atheroprotective roles of smooth muscle cell phenotypic modulation and the TCF21 disease gene as revealed by single-cell analysis. Nat Med. (2019) 25:1280–89. doi: 10.1038/s41591-019-0512-5 31359001 PMC7274198

[15] Depuydt MAC Prange KHM Slenders L Ord T Elbersen D Boltjes A . Microanatomy of the human atherosclerotic plaque by single-cell transcriptomics. Circ Res. (2020) 127:1437–55. doi: 10.1161/circresaha.120.316770 32981416 PMC7641189

[16] Newman AM Liu CL Green MR Gentles AJ Feng W Xu Y . Robust enumeration of cell subsets from tissue expression profiles. Nat Methods. (2015) 12:453–7. doi: 10.1038/nmeth.3337 25822800 PMC4739640

[17] Liu C Zhang H Chen Y Wang S Chen Z Liu Z . Identifying RBM47, HCK, CD53, TYROBP, and HAVCR2 as hub genes in advanced atherosclerotic plaques by network-based analysis and validation. Front Genet. (2020) 11:602908. doi: 10.3389/fgene.2020.602908 33519905 PMC7844323

[18] Kilkenny C Browne WJ Cuthi I Emerson M Altman DG . Improving bioscience research reporting: The ARRIVE guidelines for reporting animal research. Vet Clin Pathol. (2012) 41:27–31. doi: 10.1016/j.joca.2012.02.010 22390425

[19] Libby P . The changing landscape of atherosclerosis. Nature. (2021) 592:524–33. doi: 10.1038/s41586-021-03392-8 33883728

[20] Kong P Cui ZY Huang XF Zhang DD Guo RJ Han M . Inflammation and atherosclerosis: Signaling pathways and therapeutic intervention. Signal Transduct Target Ther. (2022) 7:131. doi: 10.1038/s41392-022-00955-7 35459215 PMC9033871

[21] Wolf D Ley K . Immunity and inflammation in atherosclerosis. Circ Res. (2019) 124:315–27. doi: 10.1161/circresaha.118.313591 30653442 PMC6342482

[22] Fernandez DM Rahman AH Fernandez NF Chudnovskiy A Amir ED Amadori L . Single-cell immune landscape of human atherosclerotic plaques. Nat Med. (2019) 25:1576–88. doi: 10.1038/s41591-019-0590-4 31591603 PMC7318784

[23] Pan H Xue C Auerbach BJ Fan J Bashore AC Cui J . Single-cell genomics reveals a novel cell state during smooth muscle cell phenotypic switching and potential therapeutic targets for atherosclerosis in mouse and human. Circulation. (2020) 142:2060–75. doi: 10.1161/circulationaha.120.048378 32962412 PMC8104264

[24] de Sousa Mesquita AP de Araujo Lopes S Pernambuco Filho PCA Nader HB Lopes CC . Acquisition of anoikis resistance promotes alterations in the Ras/ERK and PI3K/Akt signaling pathways and matrix remodeling in endothelial cells. Apoptosis. (2017) 22:1116–37. doi: 10.1007/s10495-017-1392-0 28653224

[25] Zhao T Su Z Li Y Zhang X You Q . Chitinase-3 like-protein-1 function and its role in diseases. Signal Transduct Target Ther. (2020) 5:201. doi: 10.1038/s41392-020-00303-7 32929074 PMC7490424

[26] Foks AC Ran IA Wasserman L Frodermann V Ter Borg MN de Jager SC . T-cell immunoglobulin and mucin domain 3 acts as a negative regulator of atherosclerosis. Arterioscler Thromb Vasc Biol. (2013) 33:2558–65. doi: 10.1161/atvbaha.113.301879 23990206

[27] Wang F Zhou F Peng J Chen H Xie J Liu C . Macrophage Tim-3 maintains intestinal homeostasis in DSS-induced colitis by suppressing neutrophil necroptosis. Redox Biol. (2024) 70:103072. doi: 10.1016/j.redox.2024.103072 38330550 PMC10865407

[28] Cui L Chen L Dai Y Ou J Qiu M Wang S . Increased level of Tim-3(+)PD-1(+)CD4(+)T cells with altered function might be associated with lower extremity arteriosclerosis obliterans. Front Immunol. (2022) 13:871362. doi: 10.3389/fimmu.2022.871362 35757718 PMC9229777

[29] Chen Y Kennedy DJ Ramakrishnan DP Yang M Huang W Li Z . Oxidized LDL-bound CD36 recruits an Na(+)/K(+)-ATPase-Lyn complex in macrophages that promotes atherosclerosis. Sci Signal. (2015) 8:ra91. doi: 10.1126/scisignal.aaa9623 PMC4852751 26350901

[30] Chen Y Yang M Huang W Chen W Zhao Y Schulte ML . Mitochondrial metabolic reprogramming by CD36 signaling drives macrophage inflammatory responses. Circ Res. (2019) 125:1087–102. doi: 10.1161/circresaha.119.315833 31625810 PMC6921463

[31] Ma C Li Y Tian M Deng Q Qin X Lu H . Gsalpha regulates macrophage foam cell formation during atherosclerosis. Circ Res. (2024) 134:e34–51. doi: 10.1161/circresaha.123.323156 38375634 PMC10978275

[32] Liu X Guo JW Lin XC Tuo YH Peng WL He SY . Macrophage NFATc3 prevents foam cell formation and atherosclerosis: Evidence and mechanisms. Eur Heart J. (2021) 42:4847–61. doi: 10.1093/eurheartj/ehab660 34570211

[33] van der Heijden T Kritikou E Venema W van Duijn J van Santbrink PJ Slutter B . NLRP3 inflammasome inhibition by MCC950 reduces atherosclerotic lesion development in apolipoprotein E-deficient mice-brief report. Arterioscler Thromb Vasc Biol. (2017) 37:1457–61. doi: 10.1161/atvbaha.117.309575 28596375

[34] Johnson JL . Metalloproteinases in atherosclerosis. Eur J Pharmacol. (2017) 816:93–106. doi: 10.1016/j.ejphar.2017.09.007 28893577

[35] Al-Yafeai Z Yurdagul A Jr. Peretik JM Alfaidi M Murphy PA Orr AW . Endothelial FN (fibronectin) deposition by alpha5beta1 integrins drives atherogenic inflammation. Arterioscler Thromb Vasc Biol. (2018) 38:2601–14. doi: 10.1161/atvbaha.118.311705 30354234 PMC6209122

[36] Yun S Hu R Schwaemmle ME Scherer AN Zhuang Z Koleske AJ . Integrin alpha5beta1 regulates PP2A complex assembly through PDE4D in atherosclerosis. J Clin Invest. (2019) 129:4863–74. doi: 10.1172/jci127692 31408443 PMC6819111

[37] Malik R Dau T Gonik M Sivakumar A Deredge DJ Edeleva EV . Common coding variant in SERPINA1 increases the risk for large artery stroke. Proc Natl Acad Sci USA. (2017) 114:3613–8. doi: 10.1073/pnas.1616301114 28265093 PMC5389268

[38] Liu Q Cui P Zheng K Wang J Jiang W Liu G . SERPINA1 gene expression in whole blood links the rs6647 variant G allele to an increased risk of large artery atherosclerotic stroke. FASEB J. (2020) 34:10107–16. doi: 10.1096/fj.201903197r 32725952

[39] Yang YH Xie KF Yang S Wang H Ma HH Zhou M . BLNK negatively regulates innate antifungal immunity through inhibiting c-Cbl-mediated macrophage migration. Proc Natl Acad Sci USA. (2024) 121:e2400920121. doi: 10.1073/pnas.2400920121 39413134 PMC11513953

[40] Bleckwehl T Babler A Tebens M Maryam S Nyberg M Bosteen M . Encompassing view of spatial and single-cell RNA sequencing renews the role of the microvasculature in human atherosclerosis. Nat Cardiovasc Res. (2025) 4:26–44. doi: 10.1038/s44161-024-00582-1 39715784

[41] Wang Q Xue Q . Bioinformatics analysis of potential common pathogenic mechanism for carotid atherosclerosis and Parkinson's disease. Front Aging Neurosci. (2023) 15:1202952. doi: 10.3389/fnagi.2023.1202952 37649719 PMC10464527

[42] Fan J Zhu T Tian X Liu S Zhang SL . Exploration of ferroptosis and necroptosis-related genes and potential molecular mechanisms in psoriasis and atherosclerosis. Front Immunol. (2024) 15:1372303. doi: 10.3389/fimmu.2024.1372303 39072329 PMC11272566

