# Supplementary material for: Multi-omics characterization of anoikis-related hub genes and macrophage-centered immune persistence with validation of HAVCR2 in atherosclerosis
Source: Front Immunol. 2026 Sep 11;17:1918938. doi: 10.3389/fimmu.2026.1918938 (PMC13613088; doi:10.3389/fimmu.2026.1918938)
Supplement: Supplementary file 1 [file Image1.pdf]

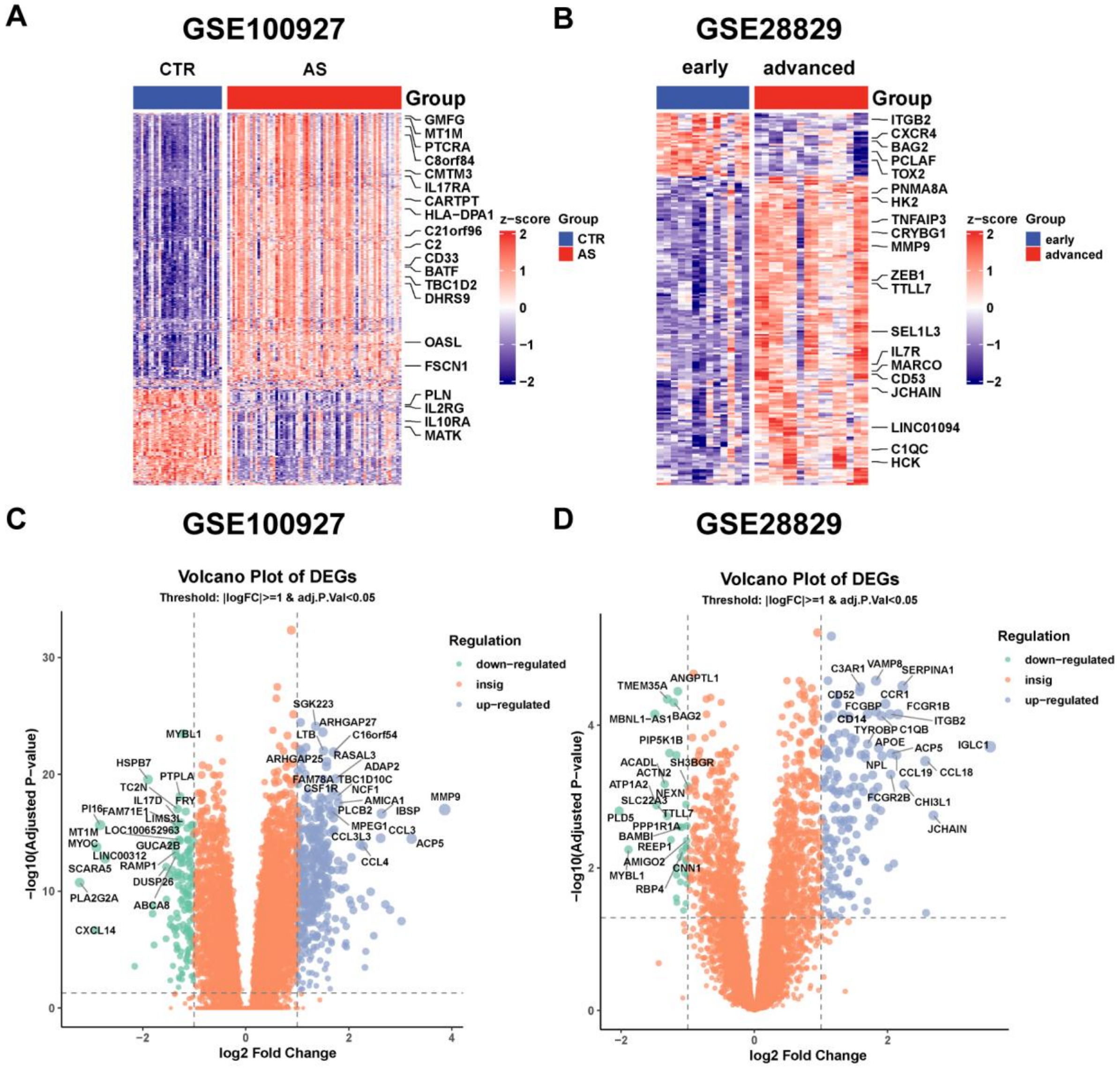

Figure S1. Differential expression landscape in two GEO cohorts.

(A) Heatmap of representative DEGs in GSE100927. Columns denote individual samples and rows denote genes; expression values are z-score scaled (red, higher; blue, lower). **(B)** Heatmap of representative DEGs in GSE28829. **(C)** Volcano plot of differential expression results in GSE100927. The x-axis indicates log2 fold change and the y-axis indicates −log10(adjusted P value). Dashed vertical lines mark |log2FC| = 1 and the dashed horizontal line marks adjusted P = 0.05. Points are colored by significance and direction (upregulated, downregulated, or not significant), and selected DEGs are annotated. **(D)** Volcano plot of differential expression results in GSE28829.

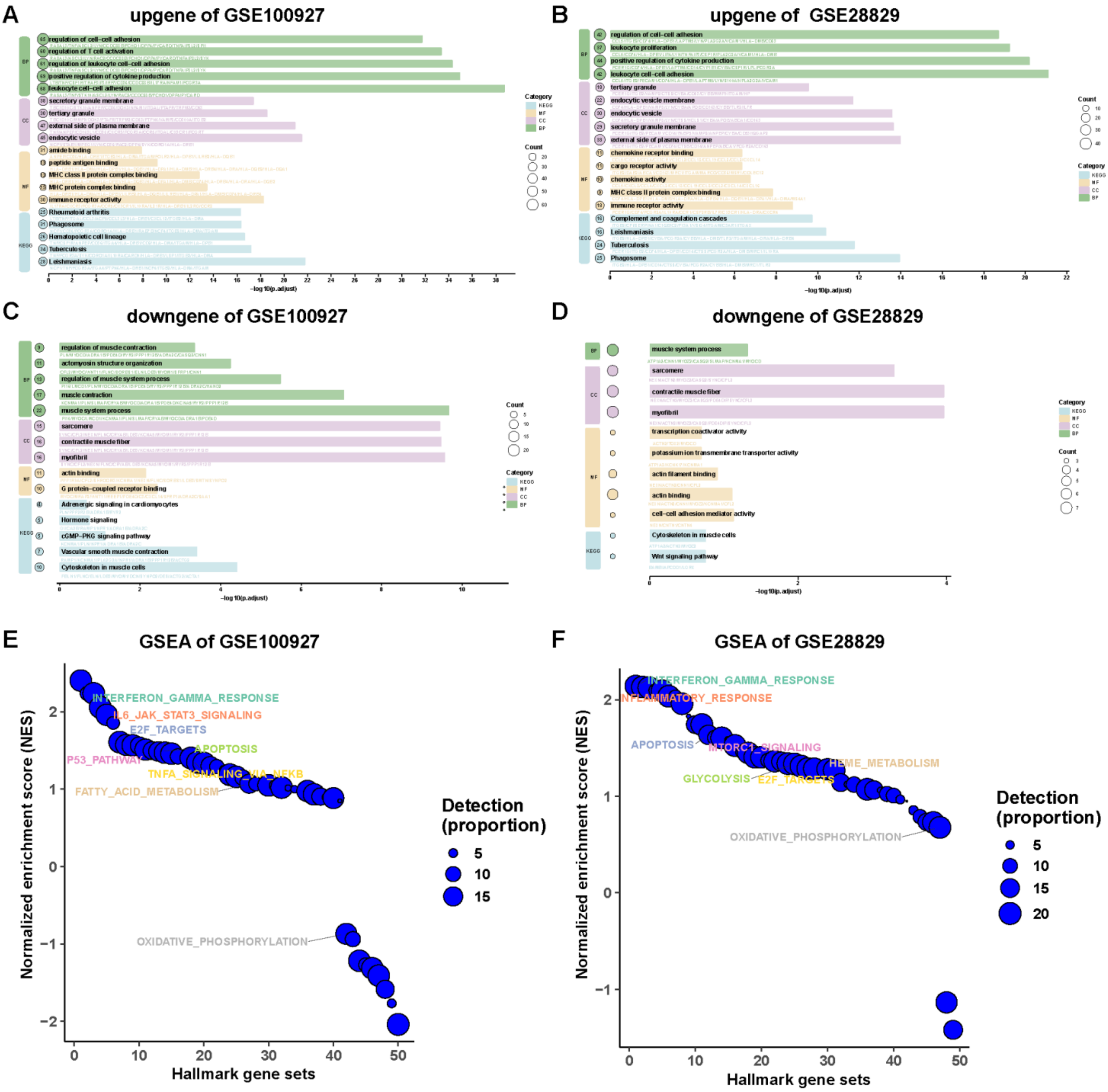

Figure S2. GO/KEGG enrichment and Hallmark GSEA of AS-associated transcriptomic alterations.

(A–B) Functional enrichment of upregulated genes in GSE100927 (A) and GSE28829 (B). Bars represent the top enriched terms from GO Biological Process (BP), Cellular Component (CC), Molecular Function (MF), and KEGG pathways, colored by category. The x-axis shows −log10(adjusted P value). Bubble size indicates the number of genes contributing to each term (Count). **(C–D)** Functional enrichment of downregulated genes in GSE100927 (C) and GSE28829 (D). **(E–F)** Hallmark GSEA results for GSE100927 (E) and GSE28829 (F). The y-axis indicates normalized enrichment score (NES) and the x-axis lists Hallmark gene sets. Bubble size denotes the detection proportion as indicated in the legend; positive versus negative NES values reflect enrichment toward the corresponding phenotype in the comparison.

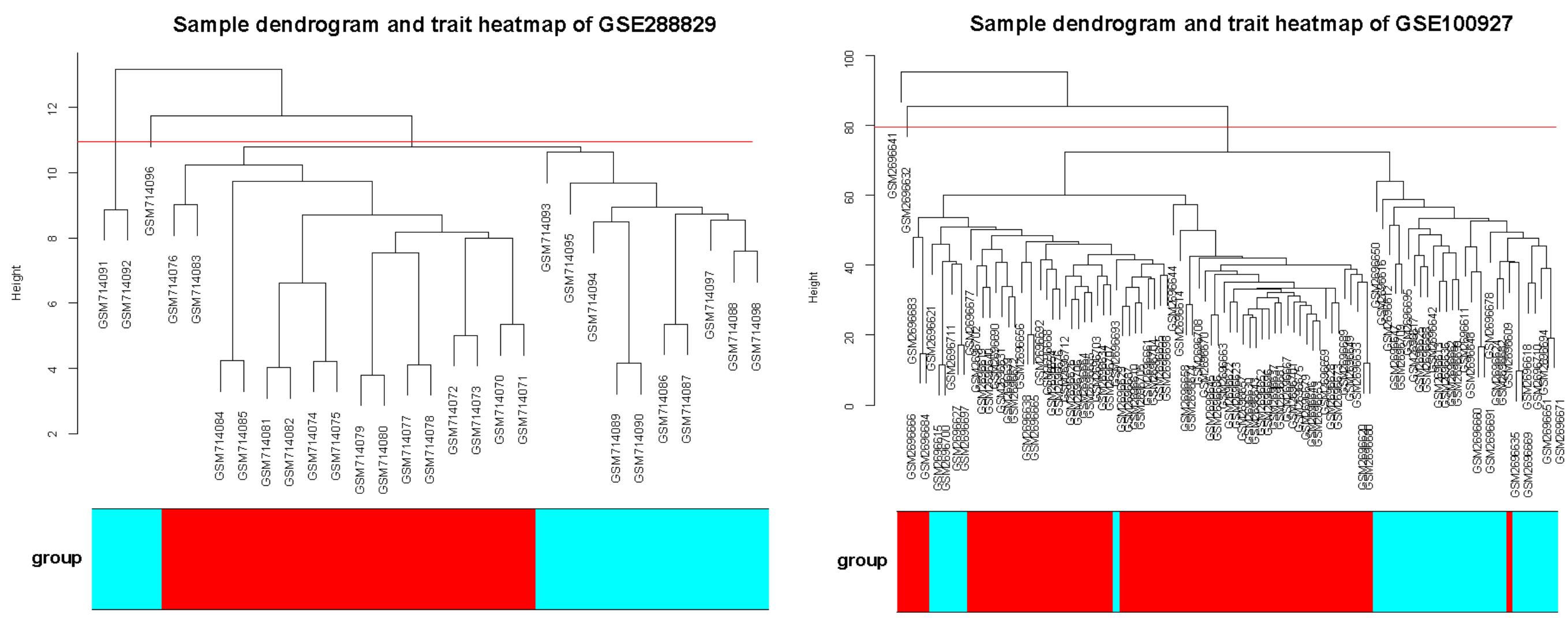

Figure S3. Sample dendrogram and trait heatmap for WGCNA quality control.

Left: GSE28829; right: GSE100927. Samples were hierarchically clustered based on their global gene-expression profiles. The red horizontal lines indicate the cut heights used to identify potential outlier samples. The trait color bars show the corresponding sample classifications: early versus advanced atherosclerotic plaques in GSE28829 and control (CTR) versus atherosclerotic (AS) tissues in GSE100927. The colors denote the two groups indicated in each panel and are aligned with the sample order in the corresponding dendrogram.

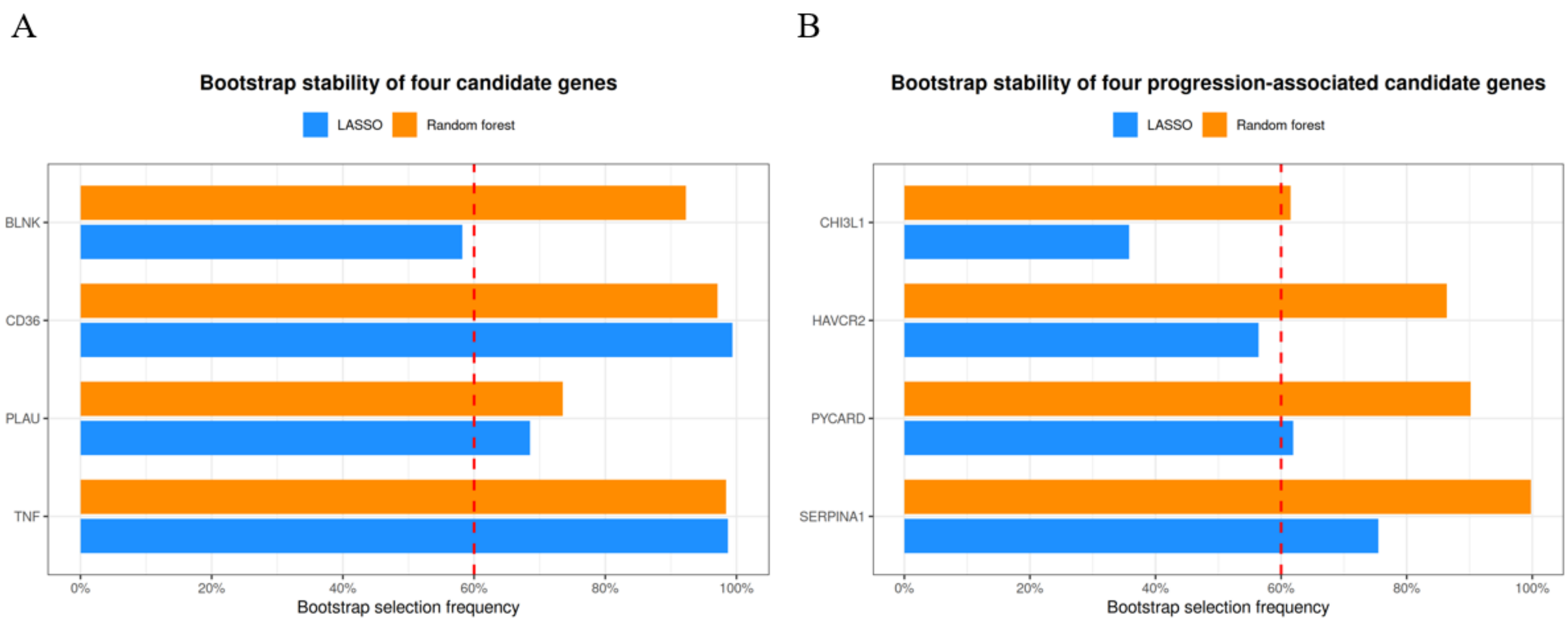

Figure S4. Bootstrap stability assessment of candidate-gene selection in GSE100927 and GSE28829.

(A) Bootstrap selection frequencies of the four disease-associated candidate genes identified from GSE100927 (BLNK, CD36, PLAU, and TNF). (B) Bootstrap selection frequencies of the four plaque-progression-associated candidate genes identified from GSE28829 (CHI3L1, HAVCR2, PYCARD, and SERPINA1). Stratified bootstrap resampling was performed for 1,000 iterations, with LASSO and Random Forest feature selection repeated within each resample. Bars indicate the proportion of successful bootstrap iterations in which each gene was selected by LASSO (blue) or ranked among the top Random Forest features (orange). The red dashed line indicates the prespecified 60% stability threshold.

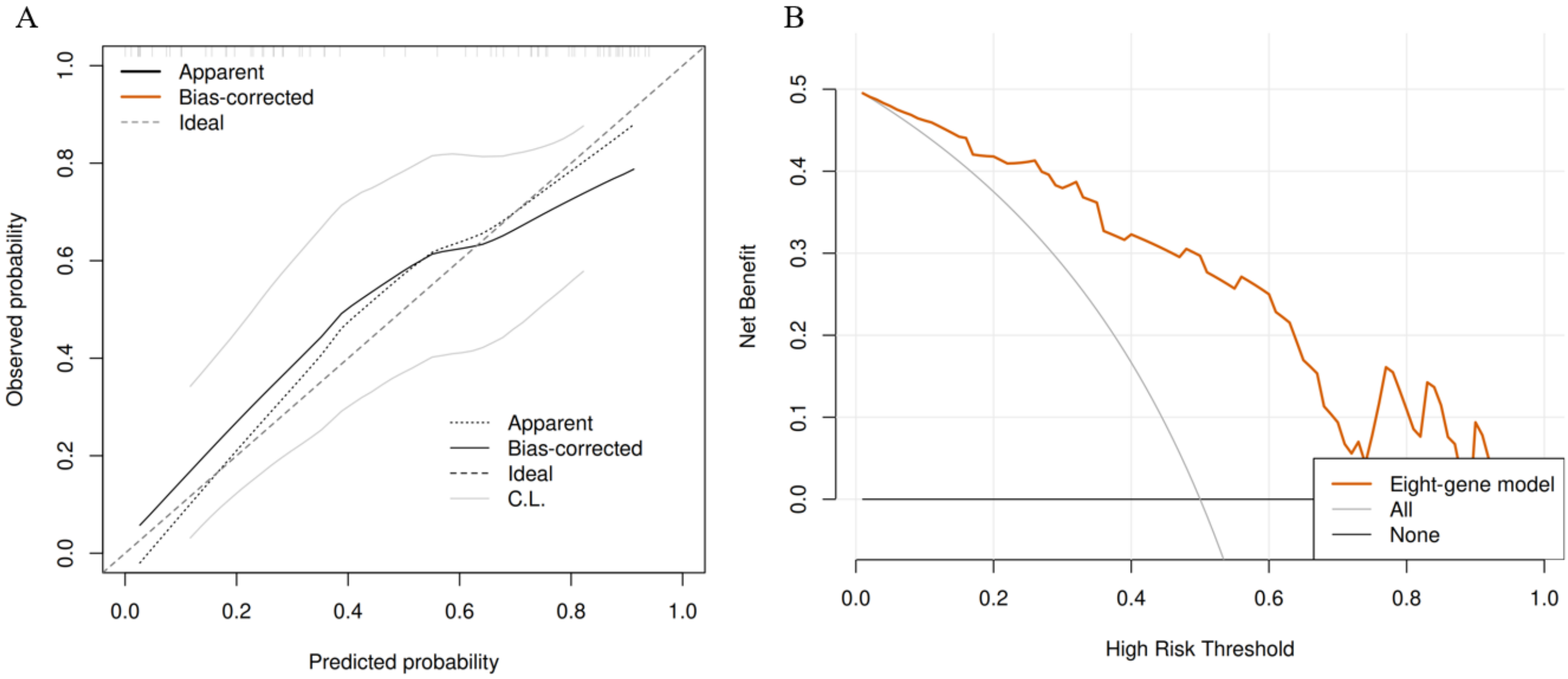

Figure S5. Internal calibration and decision-curve evaluation of the eight-gene model in the GSE43292 cohort. (A) Bootstrap-calibrated calibration curve of the eight-gene model. The apparent and bias-corrected calibration curves, ideal reference line, and corresponding confidence limits (C.L.) are shown. **(B)** Decision curve analysis of the eight-gene model. The orange line represents the eight-gene model, whereas the grey and black lines represent the “all” and “none” classification strategies, respectively. Both analyses were conducted in the GSE43292 model-development cohort and therefore represent internal model evaluation rather than external validation.

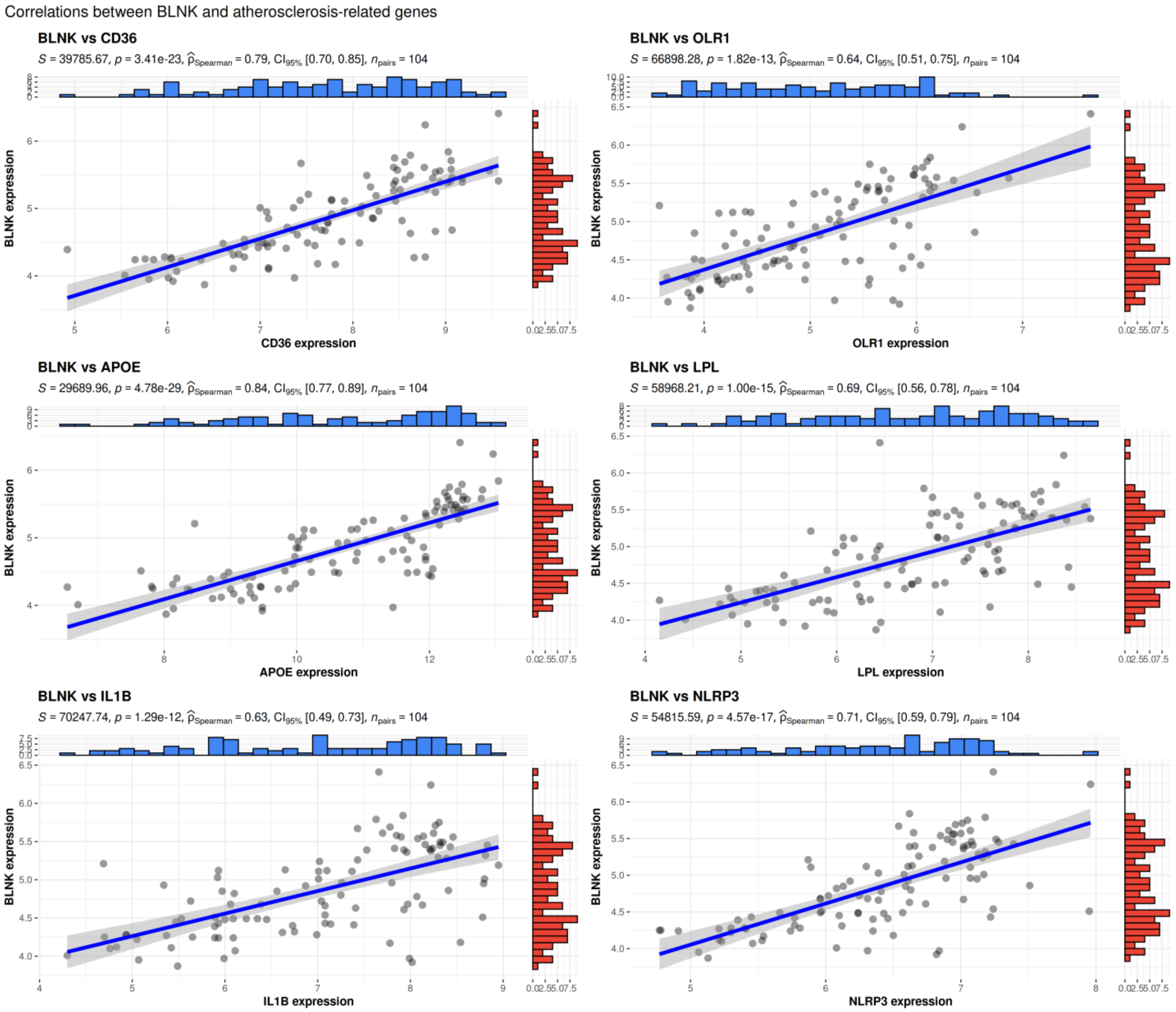

Figure S6. Correlation of BLNK Expression with Key Genes Involved in Lipid Handling, Foam-Cell Formation, and Inflammasome Activation in Atherosclerosis

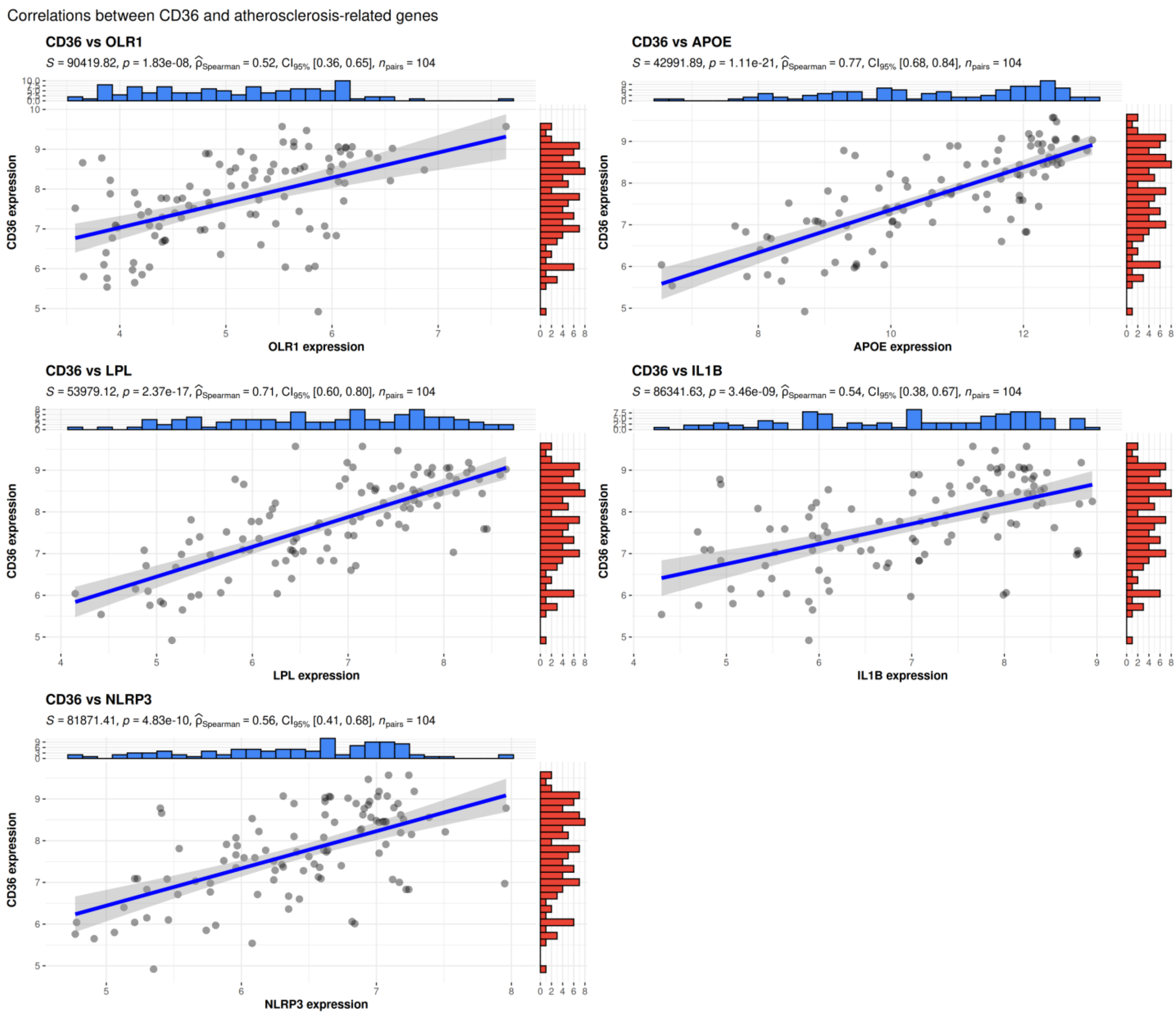

Figure S7. Correlation of CD36 Expression with Key Genes Involved in Lipid Handling, Foam-Cell Formation, and Inflammasome Activation in Atherosclerosis

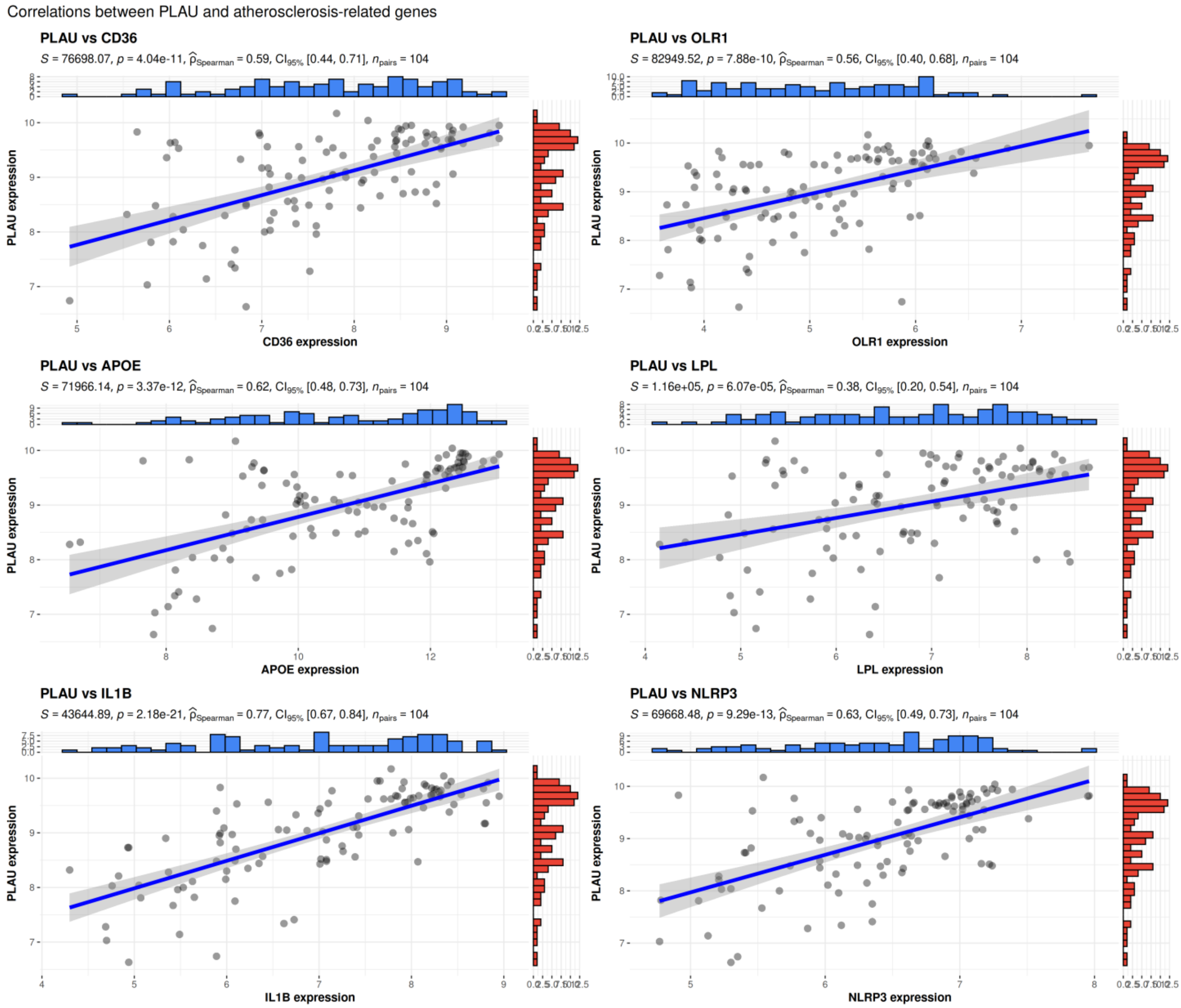

Figure S8. Correlation of PLAU Expression with Key Genes Involved in Lipid Handling, Foam-Cell Formation, and Inflammasome Activation in Atherosclerosis

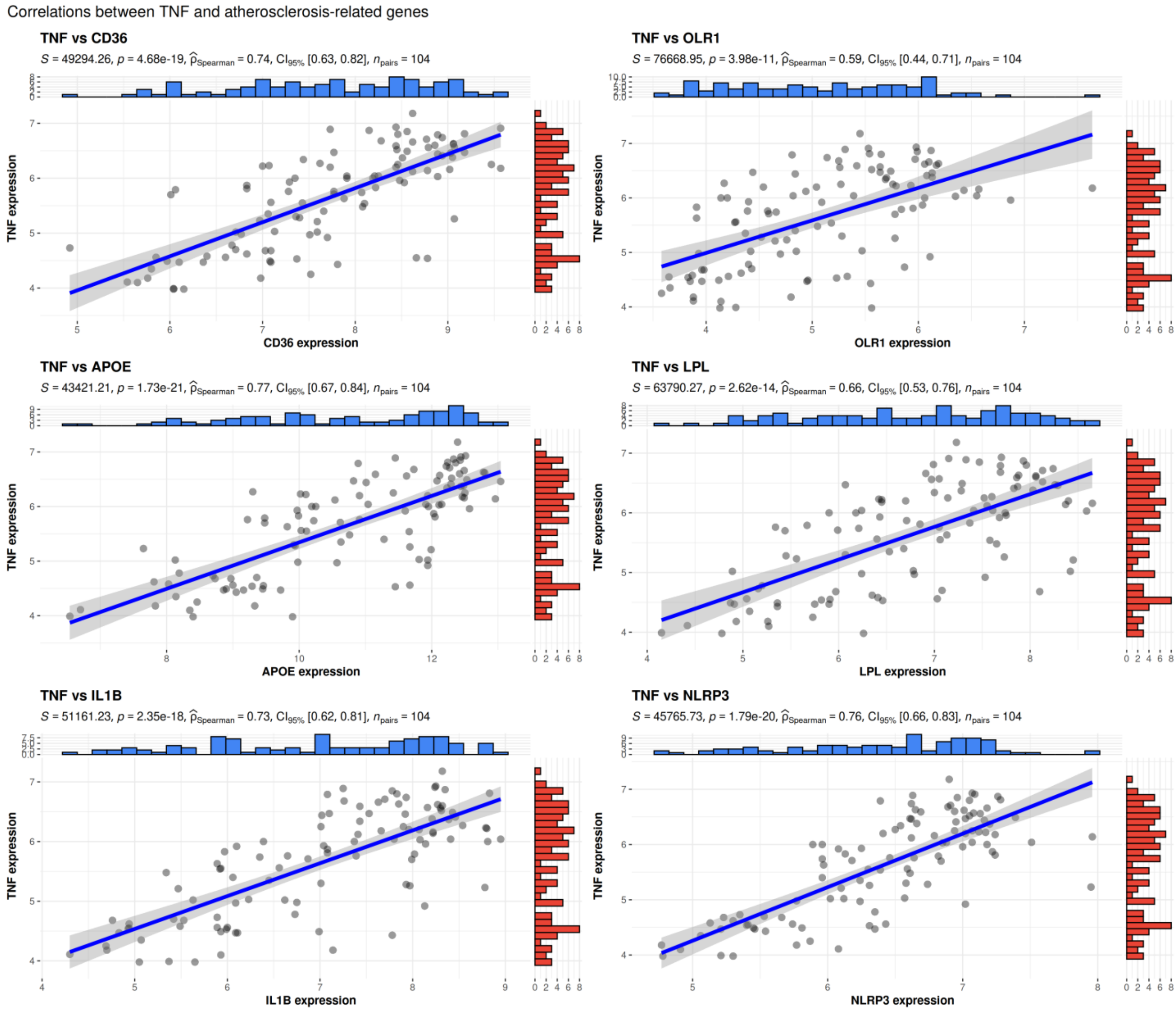

Figure S9. Correlation of TNF Expression with Key Genes Involved in Lipid Handling, Foam-Cell Formation, and Inflammasome Activation in Atherosclerosis

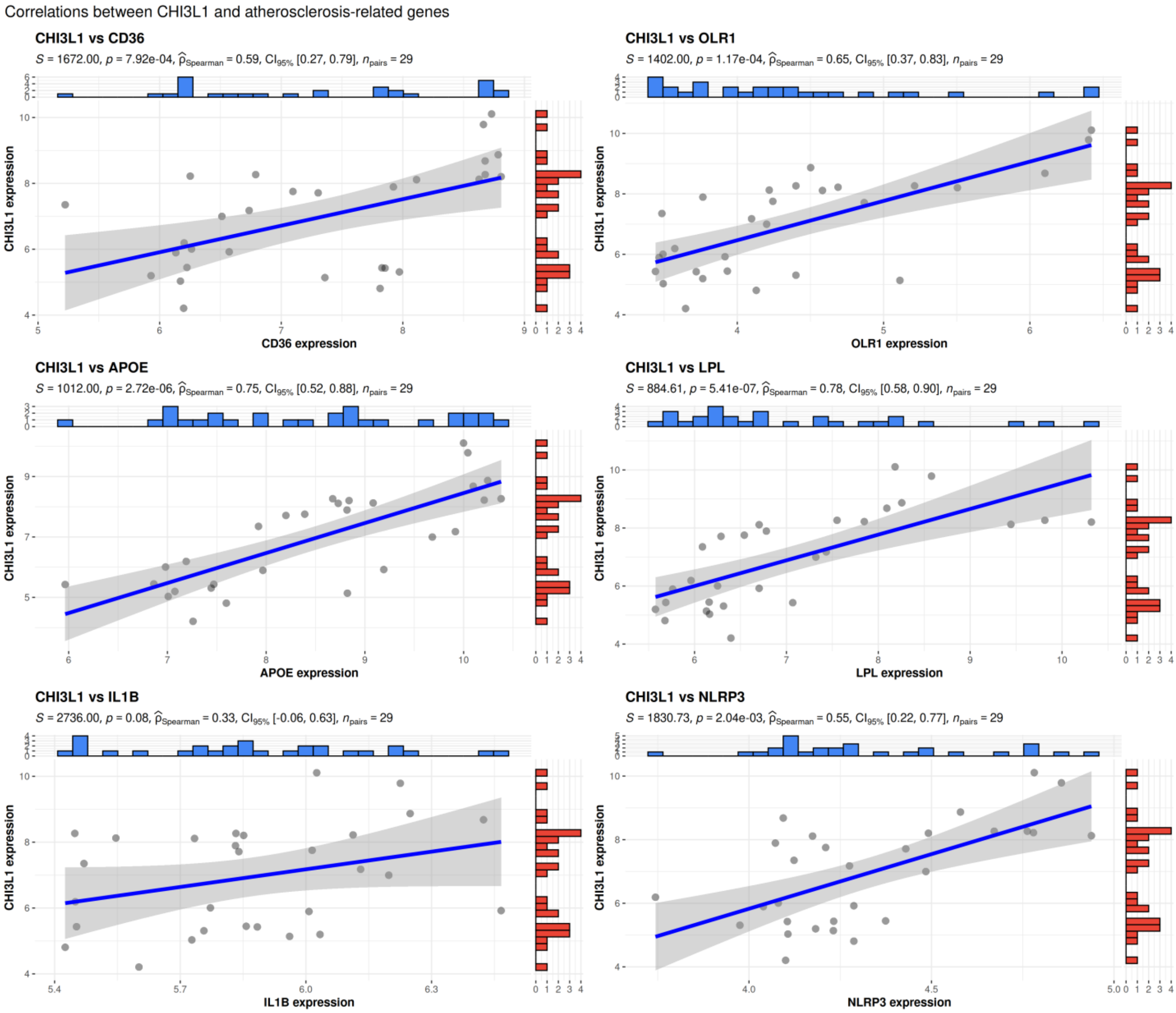

Figure S10. Correlation of CH13L1 Expression with Key Genes Involved in Lipid Handling, Foam-Cell Formation, and Inflammasome Activation in Atherosclerosis

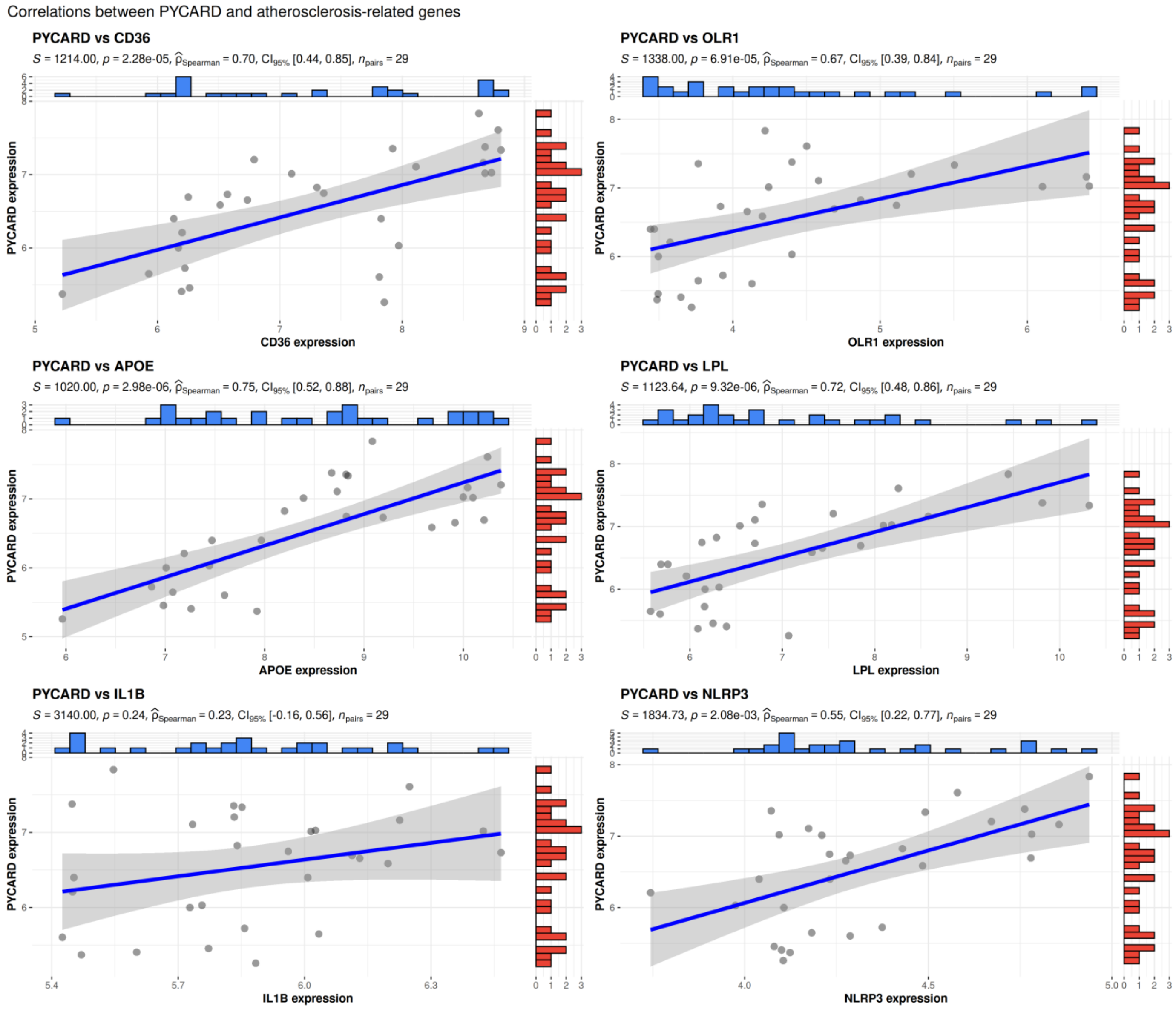

Figure S11. Correlation of PYCARD Expression with Key Genes Involved in Lipid Handling, Foam-Cell Formation, and Inflammasome Activation in Atherosclerosis

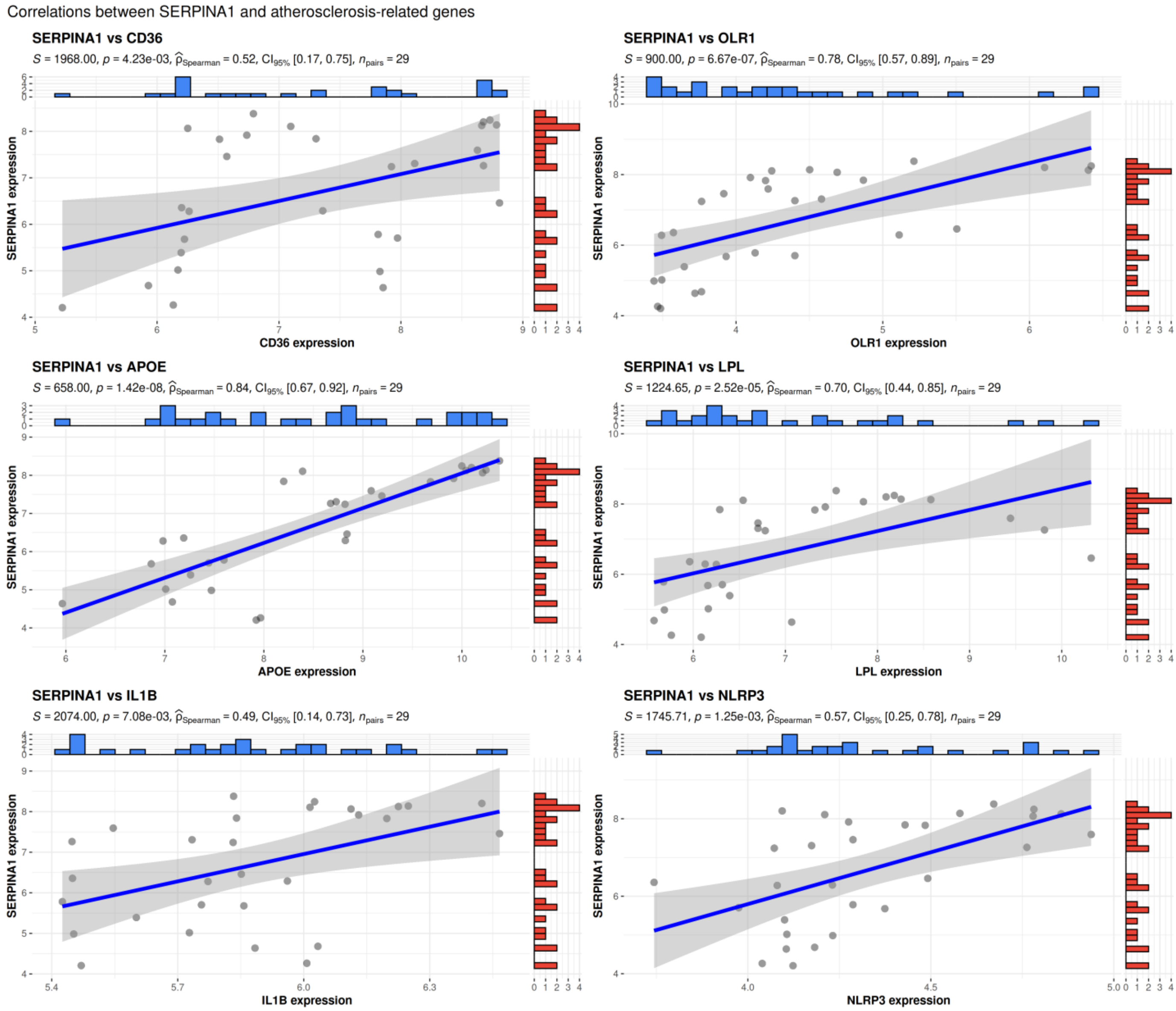

Figure S12. Correlation of SERPINA1 Expression with Key Genes Involved in Lipid Handling, Foam-Cell Formation, and Inflammasome Activation in Atherosclerosis

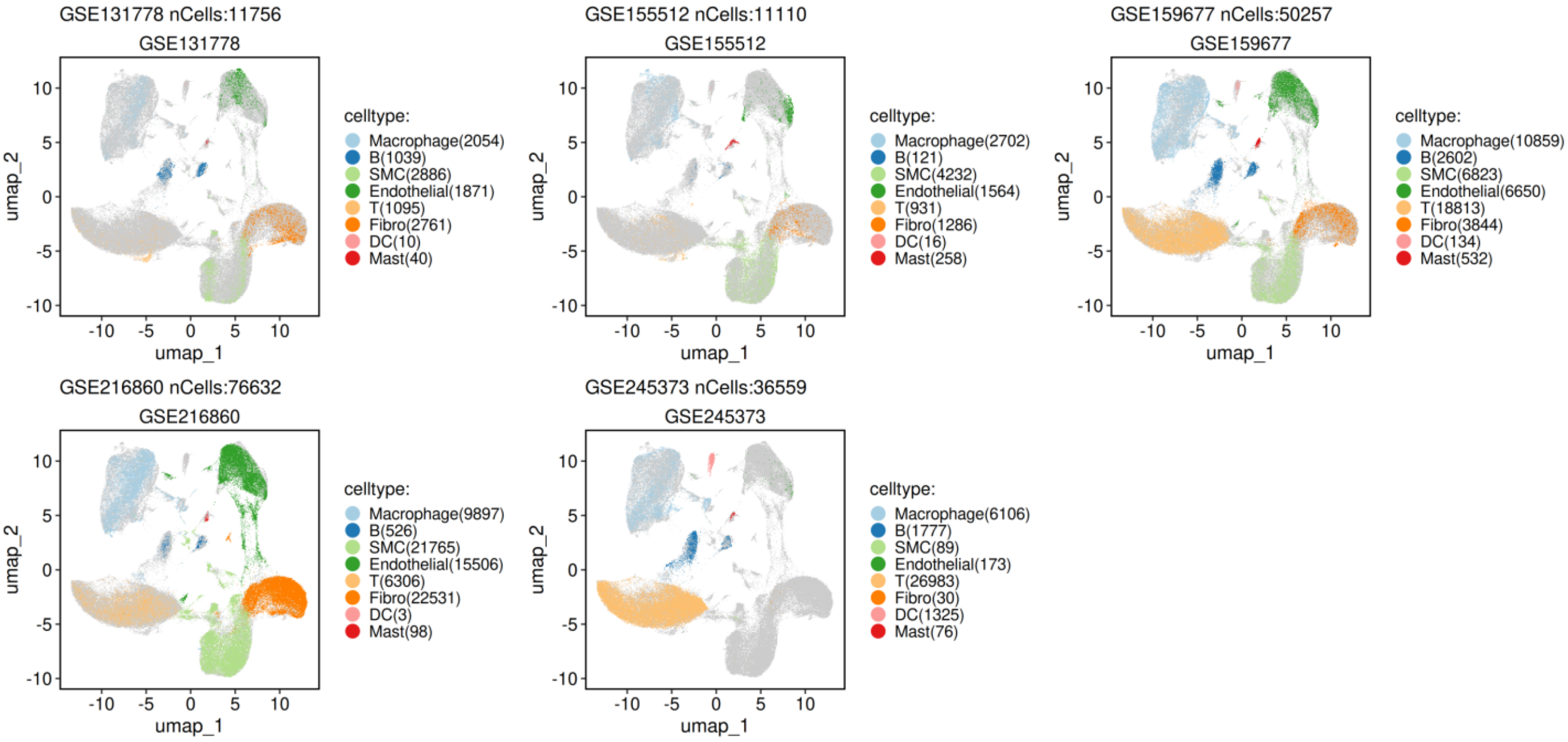

Figure S13. Dataset-specific contributions to the integrated single-cell atlas. UMAP projections showing the contribution of each scRNA-seq dataset to the integrated atlas. Each panel displays the same Harmony-integrated UMAP coordinates, with cells originating from the indicated dataset colored according to their annotated cell type and cells from the remaining datasets shown in grey. The total number of cells contributed by each dataset is indicated above the corresponding panel, while the legends report the number of cells assigned to each of the eight major cell types: macrophages, B cells, smooth muscle cells (SMCs), endothelial cells, T cells, fibroblasts, dendritic cells (DCs), and mast cells.

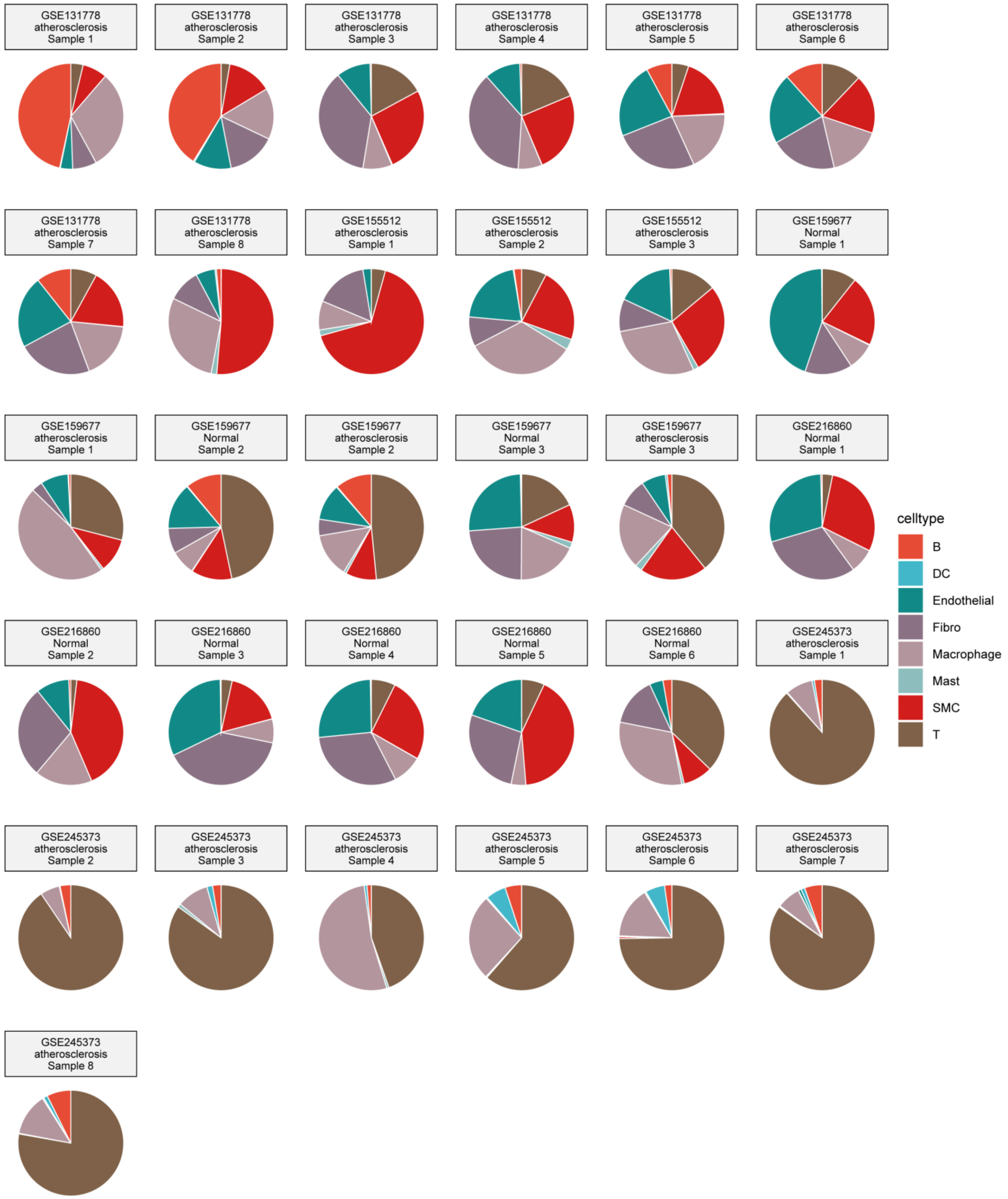

Figure S14. Sample-level cell-type composition of the integrated scRNA-seq datasets. Pie charts show the relative proportions of eight major cell types (B cells, dendritic cells, endothelial cells, fibroblasts, macrophages, mast cells, smooth muscle cells, and T cells) among quality-controlled cells from each sample in GSE131778, GSE155512, GSE159677, GSE216860, and GSE245373. Each panel is labelled by dataset, pathological group, and sample identifier, and each sector represents the proportion of captured cells assigned to the corresponding cell type. The sample-level variation reflects differences in tissue origin, disease context, cell-capture strategy, and dataset composition. GSE245373 was generated from pre-sorted viable CD45+ cells and therefore primarily contributed immune-cell populations.

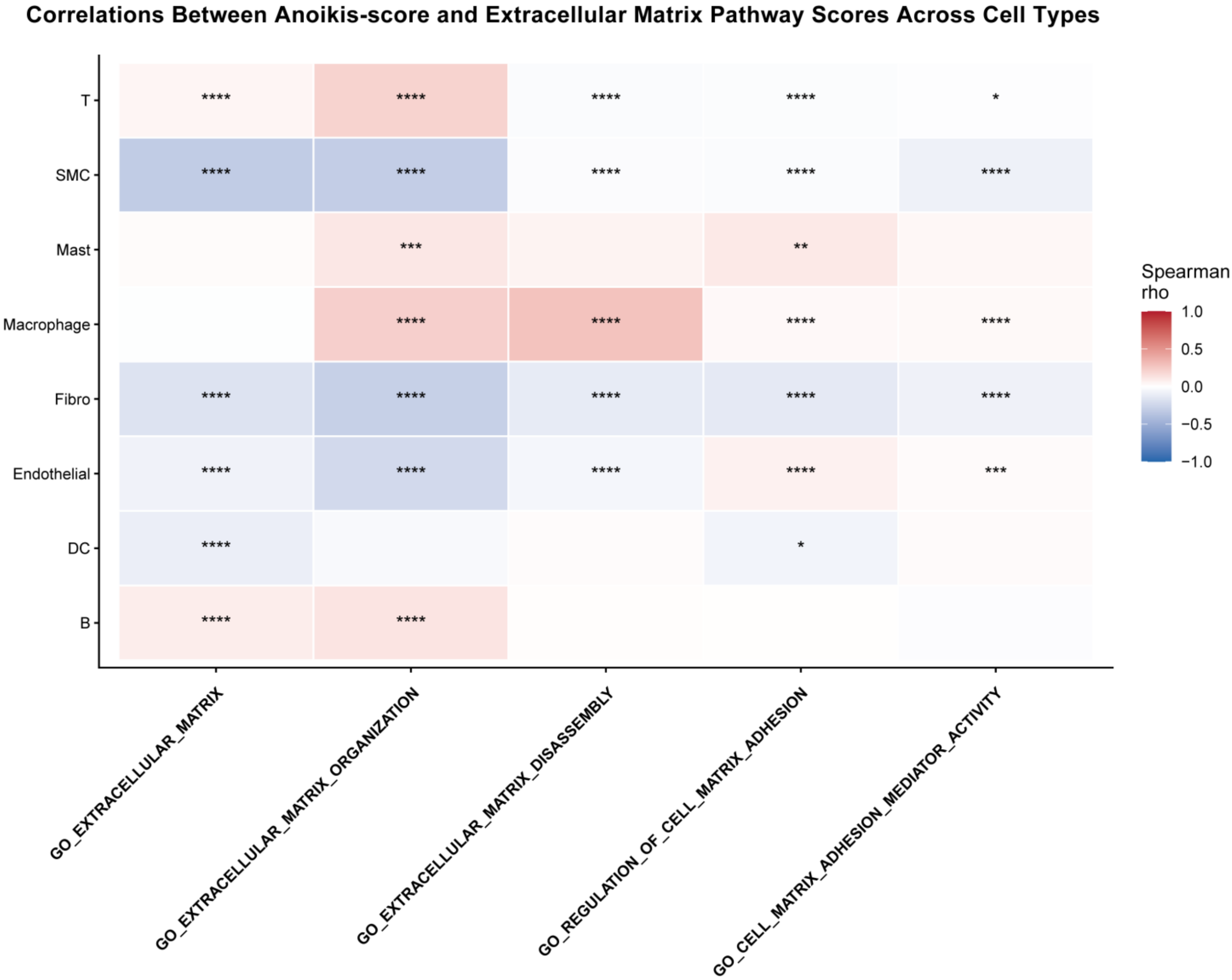

Figure S15. Correlations between Anoikis-score1 and extracellular-matrix-related gene-set scores across major cell types. Heatmap showing cell-type-stratified Spearman correlations between Anoikis-score1 and five Gene Ontology gene-set scores: extracellular matrix, extracellular matrix organization, extracellular matrix disassembly, regulation of cell–matrix adhesion, and cell–matrix adhesion mediator activity. Colors represent Spearman's rank correlation coefficient (ρ), with red indicating positive and blue indicating negative correlations. Asterisks denote nominal two-sided statistical significance: *P < 0.05, **P < 0.01, ***P < 0.001, and ****P < 0.0001. SMC, smooth muscle cells; DC, dendritic cells; Fibro, fibroblasts.

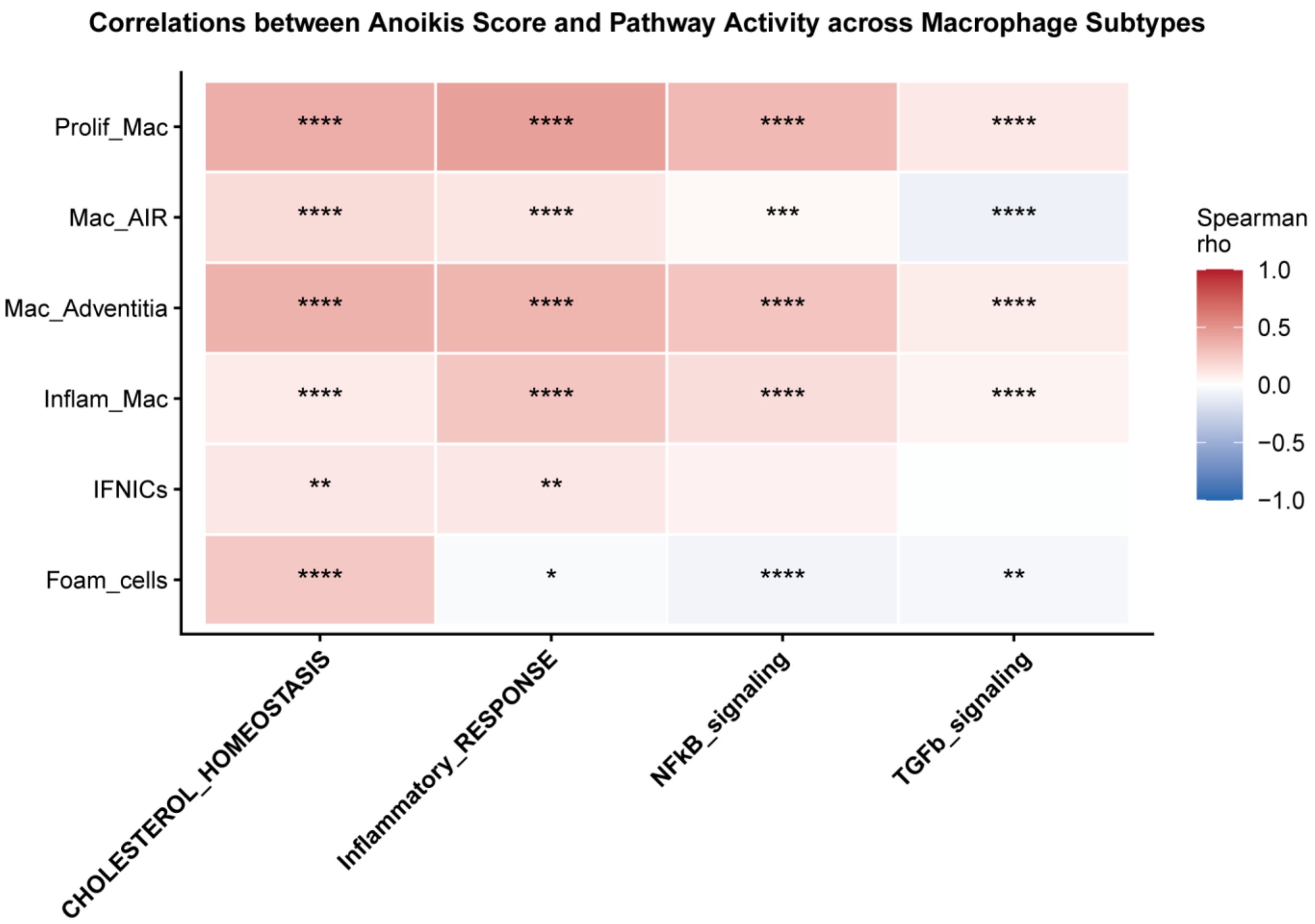

Figure S16. Correlations between Anoikis-score1 and pathway-related gene-set scores across macrophage states. Heatmap showing Spearman correlations between Anoikis-score1 and gene-set scores for cholesterol homeostasis, inflammatory response, TNF-α signaling via NF-κB, and TGF-β signaling within six macrophage states: Prolif_Mac, Mac_AIR, Mac_Adventitia, Inflam_Mac, IFNICs, and Foam_cells. Colors represent Spearman's rank correlation coefficient (ρ), with red indicating positive and blue indicating negative correlations. Asterisks denote nominal two-sided statistical significance: *$P < 0.05$, **$P < 0.01$, ***$P < 0.001$, and ****$P < 0.0001$.

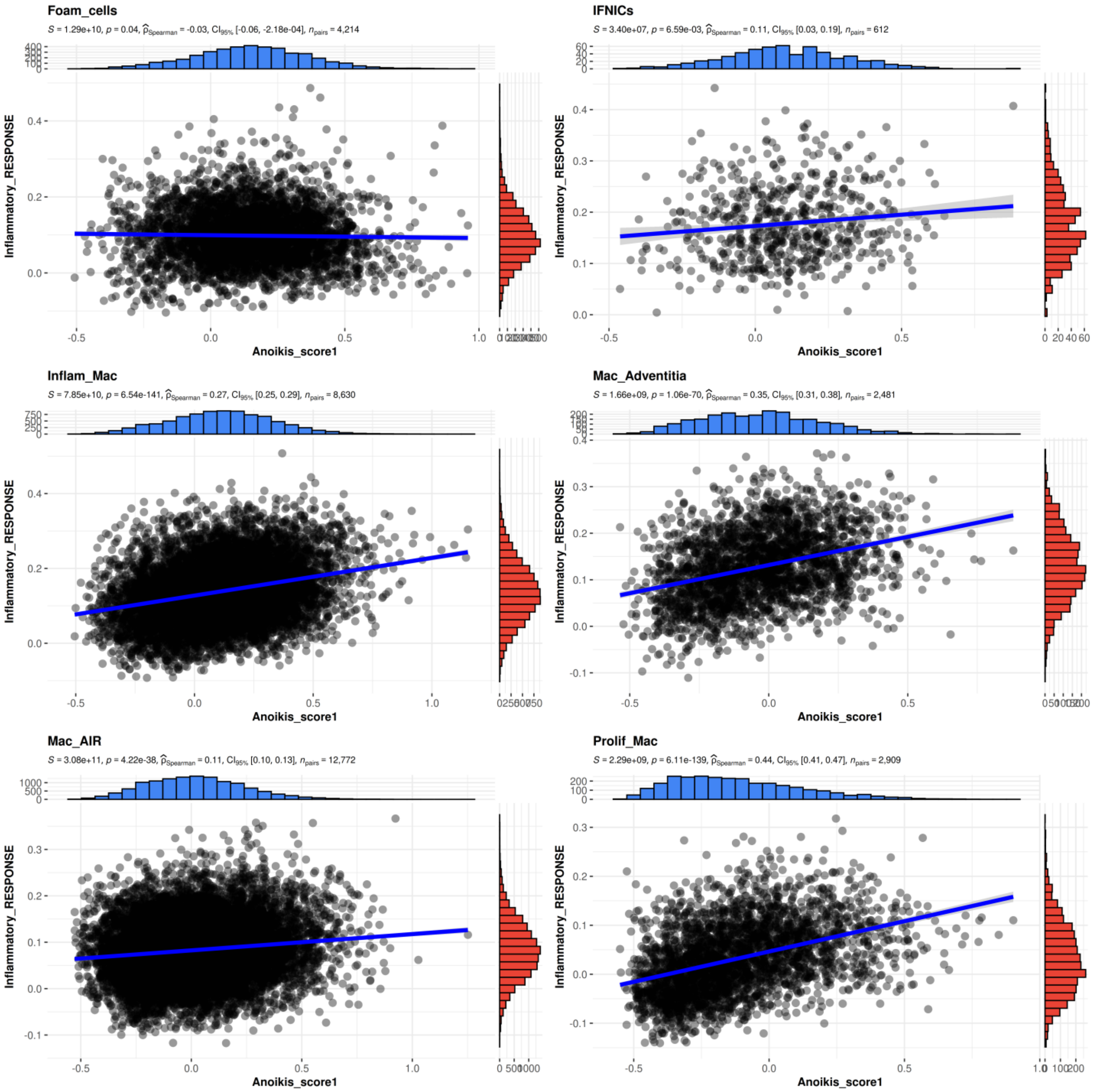

Figure S17. Associations between Anoikis-score1 and inflammatory-response gene-set scores across macrophage states. Cell-level scatterplots show the relationships between Anoikis-score1 and inflammatory-response gene-set scores in Foam_cells, IFNICs, Inflam_Mac, Mac_Adventitia, Mac_AIR, and Prolif_Mac. Each point represents an individual cell. Blue lines indicate fitted linear trends, with shaded areas representing 95% confidence intervals. Marginal histograms show the distributions of the two scores within each macrophage state. Spearman's rank correlation coefficient (ρ), its 95% confidence interval, P value, and the number of cell pairs analyzed are reported above each panel. Correlations were calculated separately within each macrophage state.

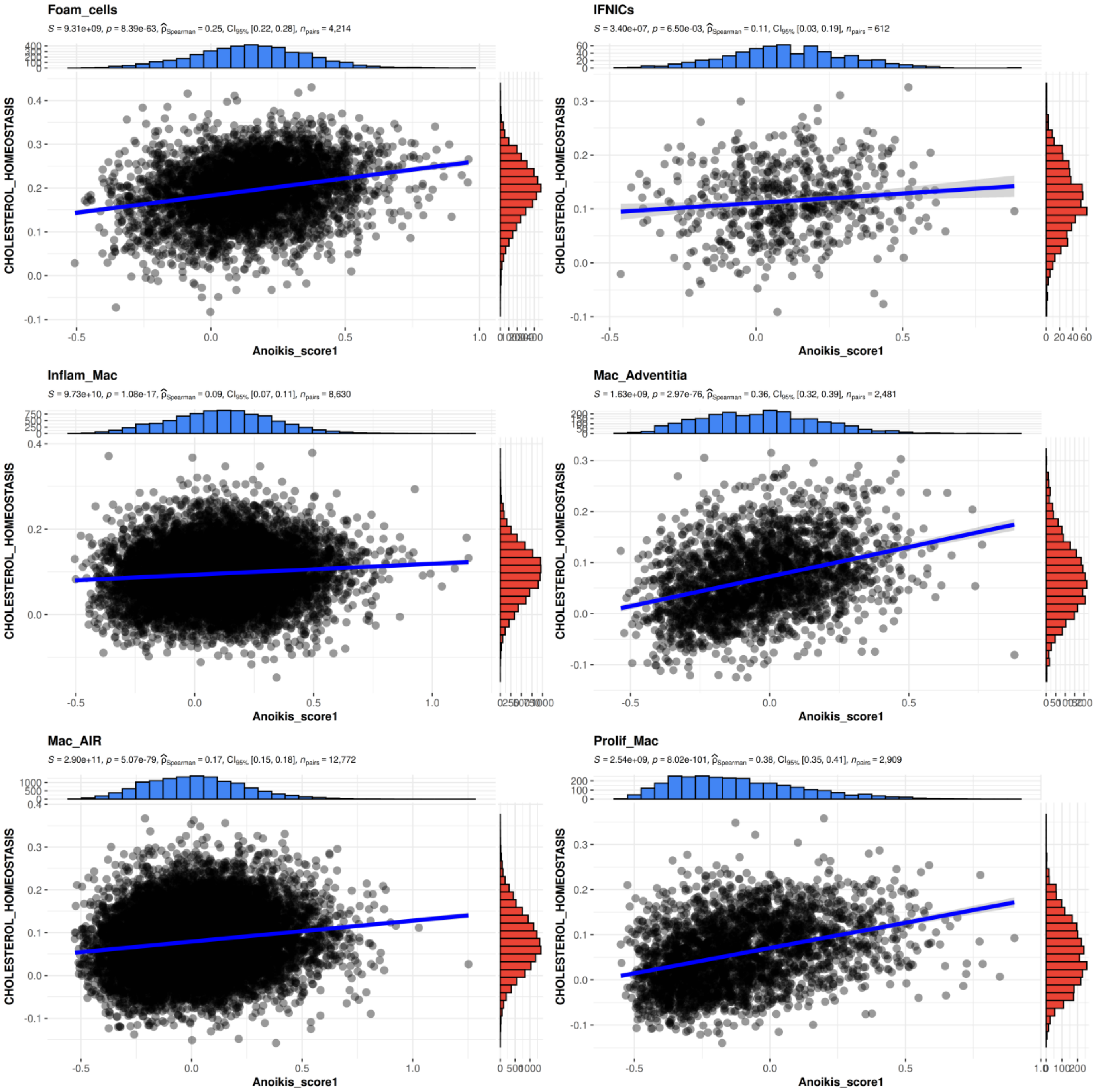

Figure S18. Associations between Anoikis-score1 and cholesterol-homeostasis gene-set scores across macrophage states. Cell-level scatterplots show the relationships between Anoikis-score1 and cholesterol-homeostasis gene-set scores in Foam_cells, IFNICs, Inflam_Mac, Mac_Adventitia, Mac_AIR, and Prolif_Mac. The cholesterol-homeostasis score was calculated using the HALLMARK_CHOLESTEROL_HOMEOSTASIS gene set. Each point represents an individual cell. Blue lines indicate fitted linear trends, with shaded areas representing 95% confidence intervals. Marginal histograms show the distributions of Anoikis-score1 and cholesterol-homeostasis scores within each macrophage state. Spearman's rank correlation coefficient (ρ), its 95% confidence interval, P value, and the number of cell pairs analyzed are reported above each panel. Correlations were calculated separately within each macrophage state.

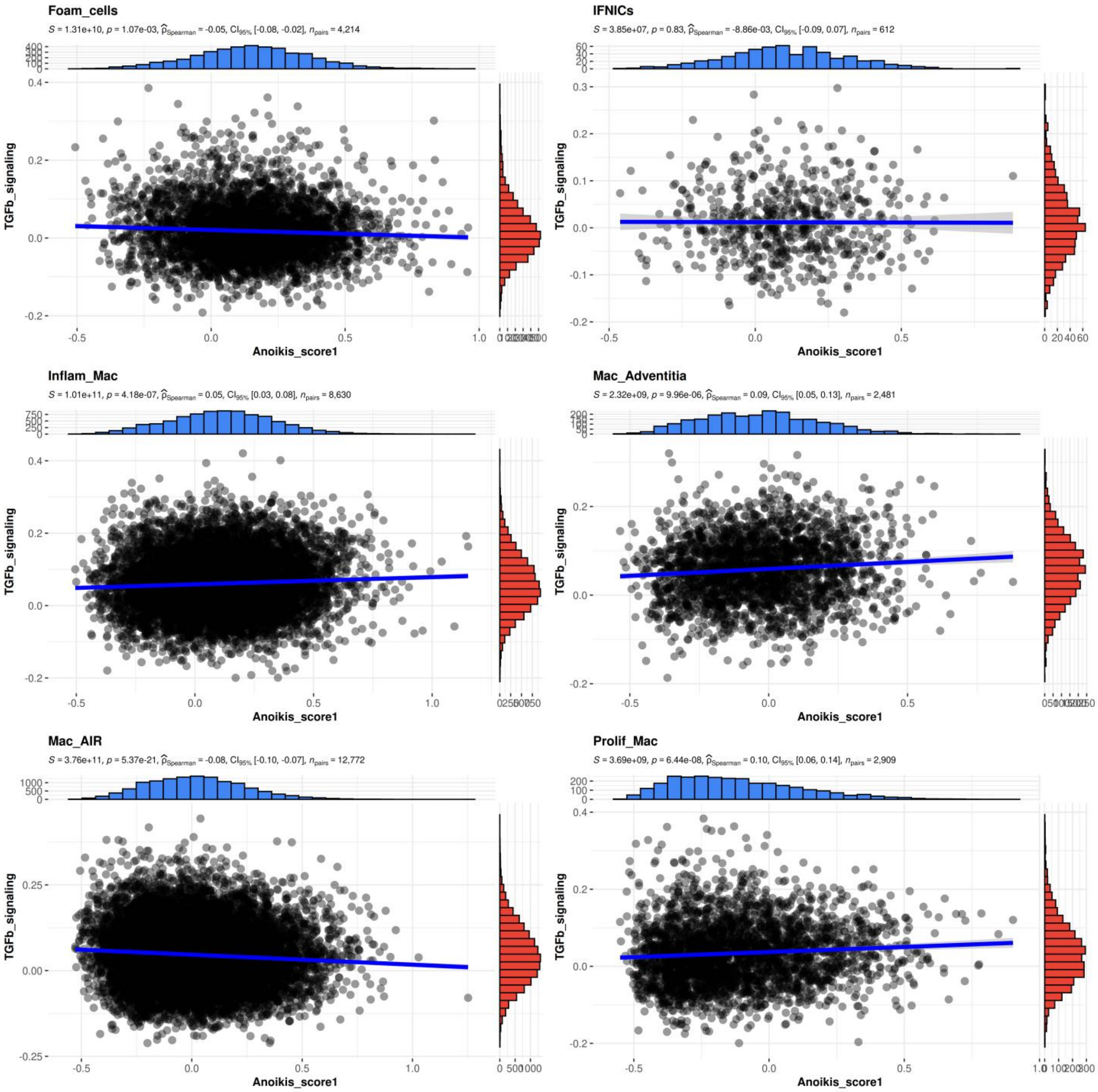

Figure S19. Associations between Anoikis-score1 and TGF-β-signaling gene-set scores across macrophage states. Cell-level scatterplots show the relationships between Anoikis-score1 and TGF-β-signaling gene-set scores in Foam_cells, IFNICs, Inflam_Mac, Mac_Adventitia, Mac_AIR, and Prolif_Mac. The TGF-β-signaling score was calculated using the HALLMARK_TGF_BETA_SIGNALING gene set. Each point represents an individual cell. Blue lines indicate fitted linear trends, with shaded areas representing 95% confidence intervals. Marginal histograms show the distributions of Anoikis-score1 and TGF-β-signaling scores within each macrophage state. Spearman's rank correlation coefficient (ρ), its 95% confidence interval, P value, and the number of cell pairs analyzed are reported above each panel. Correlations were calculated separately within each macrophage state.

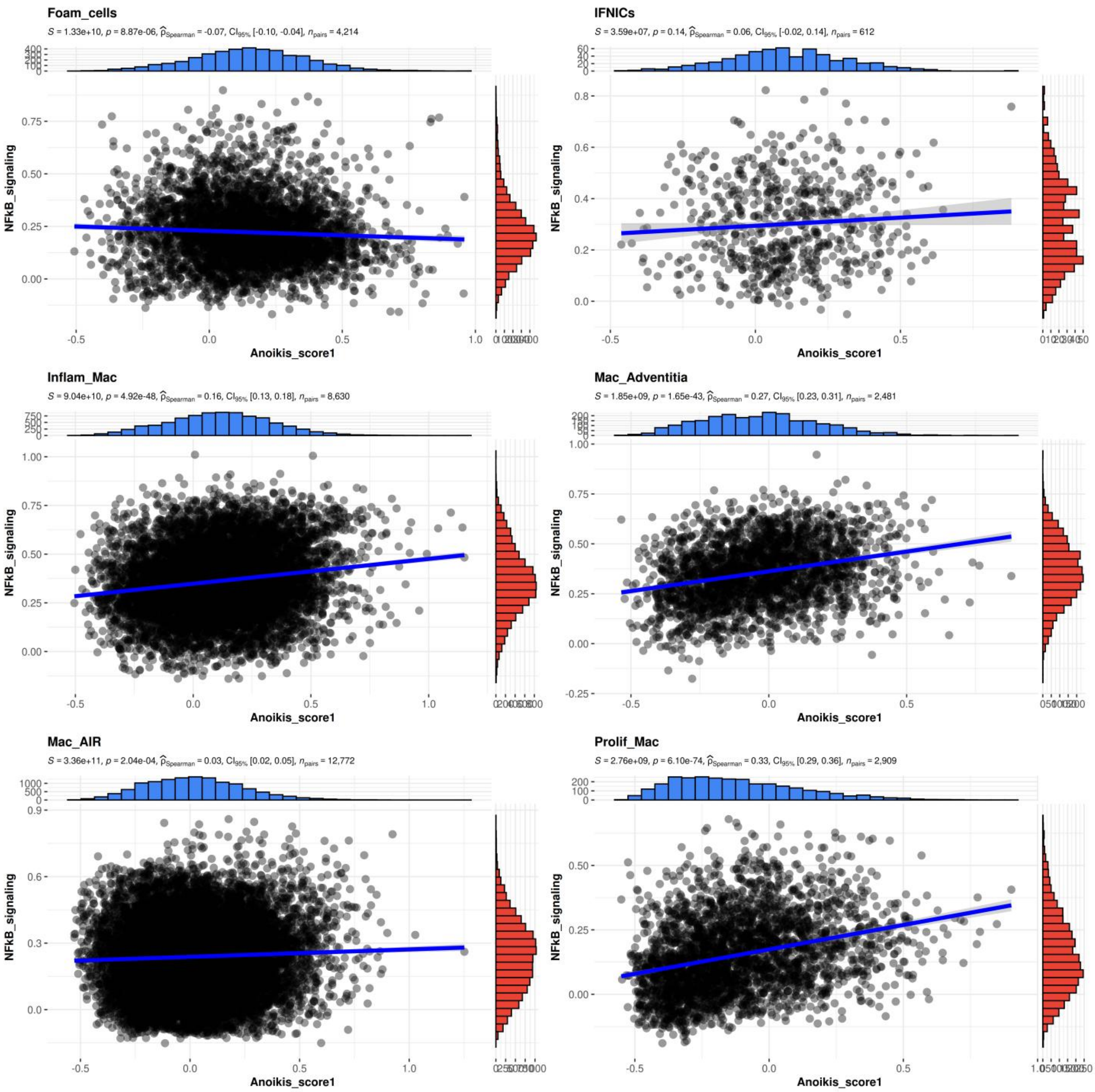

Figure S20. Associations between Anoikis-score1 and NF-κB-signaling gene-set scores across macrophage states. Cell-level scatterplots show the relationships between Anoikis-score1 and NF-κB-signaling gene-set scores in Foam_cells, IFNICs, Inflam_Mac, Mac_Adventitia, Mac_AIR, and Prolif_Mac. The NF-κB-signaling score was calculated using the HALLMARK_TNFA_SIGNALING_VIA_NFKB gene set. Each point represents an individual cell. Blue lines indicate fitted linear trends, with shaded areas representing 95% confidence intervals. Marginal histograms show the distributions of Anoikis-score1 and NF-κB-signaling scores within each macrophage state. Spearman's rank correlation coefficient (ρ), its 95% confidence interval, P value, and the number of cell pairs analyzed are reported above each panel. Correlations were calculated separately within each macrophage state.

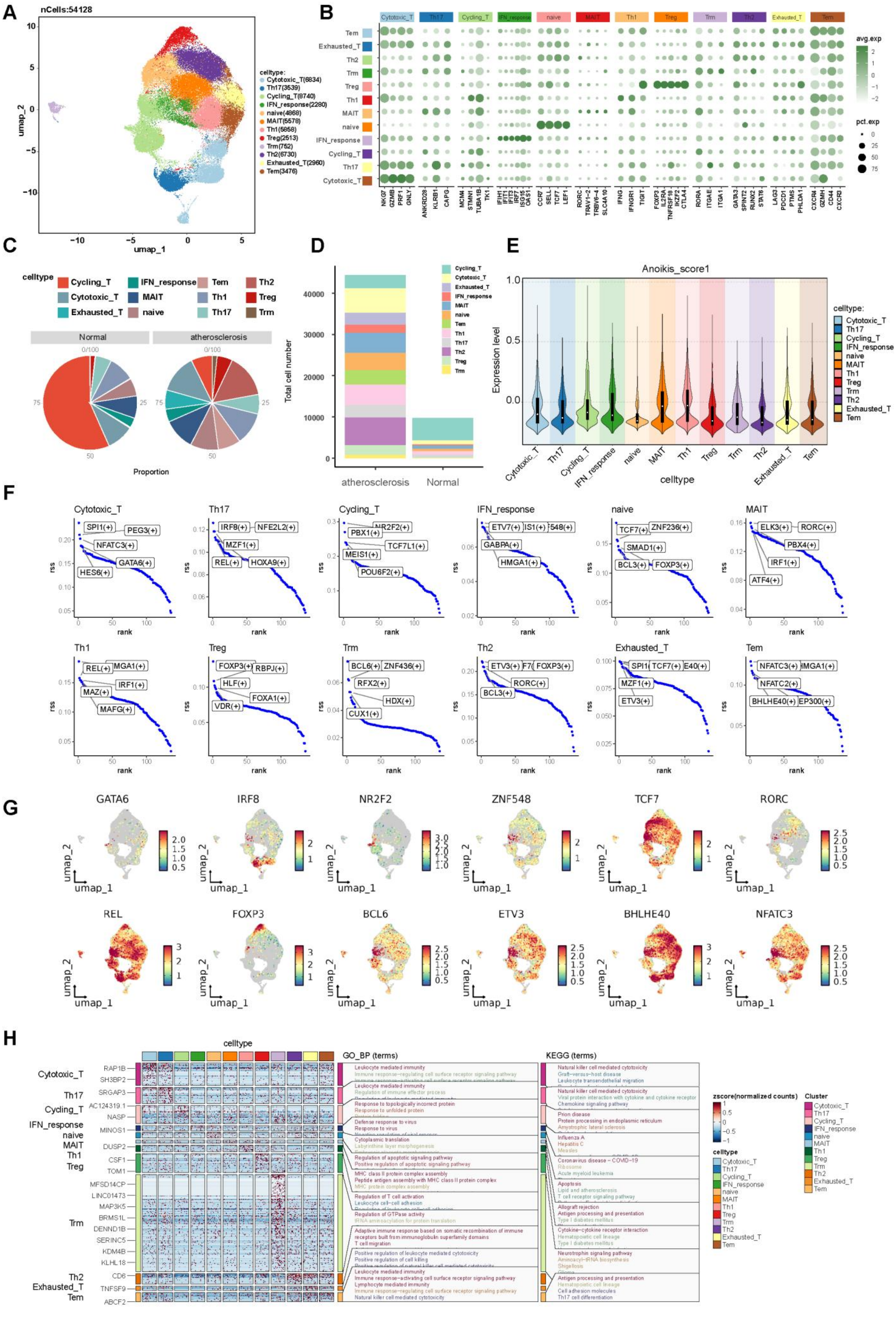

A
nCells:54128
B
C
D
E
Anoikis_score1
F
G
H

Figure S21. T-cell re-clustering defines 12 T-cell states with distinct markers, compositional shifts, anoikis activity, regulon programs, and functional enrichment.

(A) UMAP of 54,128 T cells colored by 12 states; the legend reports cell counts per state. **(B)** Dot plot of canonical marker genes across T-cell states. Dot color denotes average expression (avg.exp), and dot size indicates the fraction of expressing cells (pct.exp). **(C)** Pie charts showing relative proportions of the 12 T-cell states in CTR and AS groups. **(D)** Stacked bar plot showing absolute T-cell numbers per group partitioned by state. **(E)** Violin plots of Anoikis_score1 across T-cell states; boxes indicate median and interquartile range. **(F)** Regulon specificity score (RSS) curves for each T-cell state with top TF regulons labeled. **(G)** UMAP feature plots of representative TF/regulon activities: GATA6, IRF8, NFE2L2, ZNF548, TCF7, RORC, REL, FOXP3, BCL6, ETV3, BHLHE40, and NFATC3. **(H)** Heatmap of state-associated genes (z-score of normalized counts) across the 12 T-cell states with corresponding enriched GO Biological Process (GO_BP) and KEGG terms.

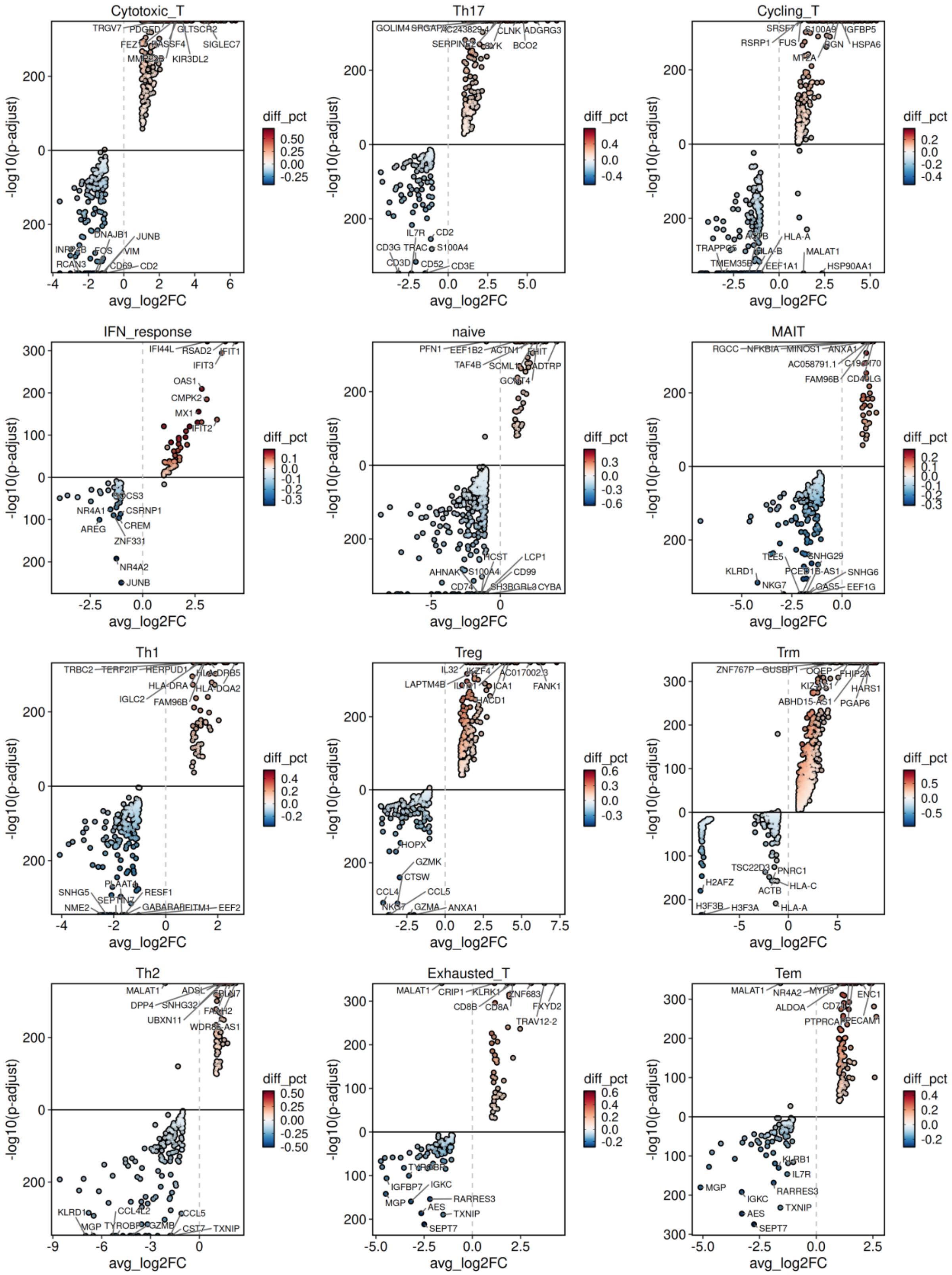

Figure S22. State-specific differential-expression profiles across T-cell subsets. Volcano plots show differential gene expression for Cytotoxic_T, Th17, Cycling_T, IFN_response, naive, MAIT, Th1, Treg, Trm, Th2, Exhausted_T, and Tem cells, with each subset compared against all other T-cell subsets. The x-axis represents the average log2 fold change (avg_log2FC). The signed y-axis represents the magnitude of the adjusted P value, with genes showing positive and negative differences in detection frequency plotted above and below zero, respectively. Dot color represents the difference in the proportion of cells expressing each gene between the indicated subset and the remaining T-cell subsets (diff_pct = pct.1 − pct.2). Genes with adjusted $P < 0.05$ and $|avg\_log2FC| \geq 1$ were considered differentially expressed. Up to five representative genes on each side of the plot are labeled after excluding ribosomal, mitochondrial, Ensembl identifier, and LINC-prefixed transcripts.

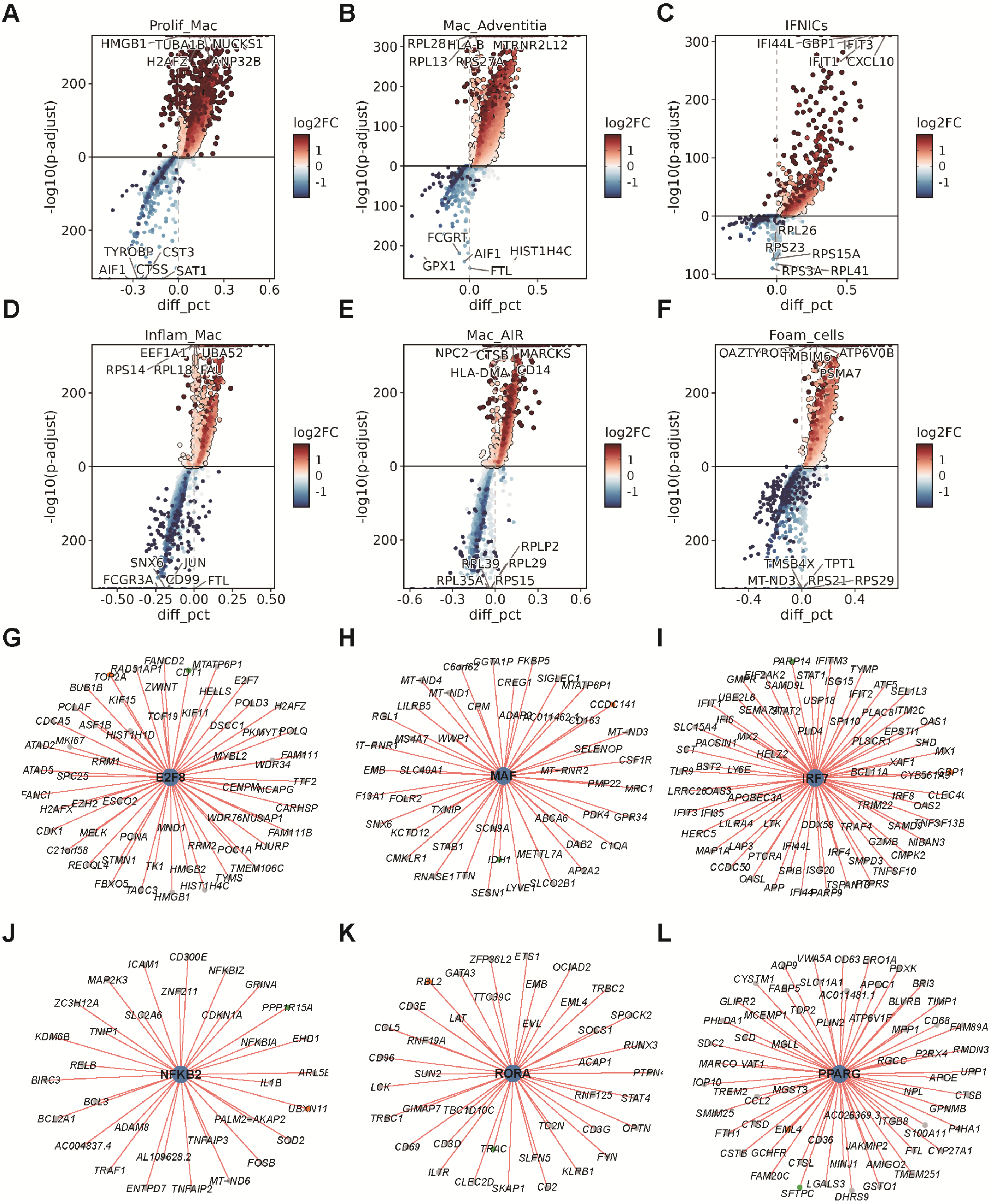

Figure S23. State-stratified differential expression and TF regulon networks of macrophage subsets.

(A–F) Volcano-style differential expression between AS and Normal/CTR within each macrophage state: Prolif_Mac (A), Mac_Adventitia (B), IFNICs (C), Inflam_Mac (D), Mac_AIR (E), and Foam_cells (F). The x-axis indicates diff_pct (difference in detection proportion between groups), and the y-axis indicates −log10(adjusted P value). Points are colored by log2FC, and representative top-ranked genes are labeled. **(G–L)** TF–target regulon network visualization for representative state-defining TFs: E2F8 (G), MAF (H), IRF7 (I), NFKB2 (J), RORA (K), and PPARG (L). Central nodes denote TFs, peripheral nodes denote inferred target genes, and edges represent predicted regulatory relationships.

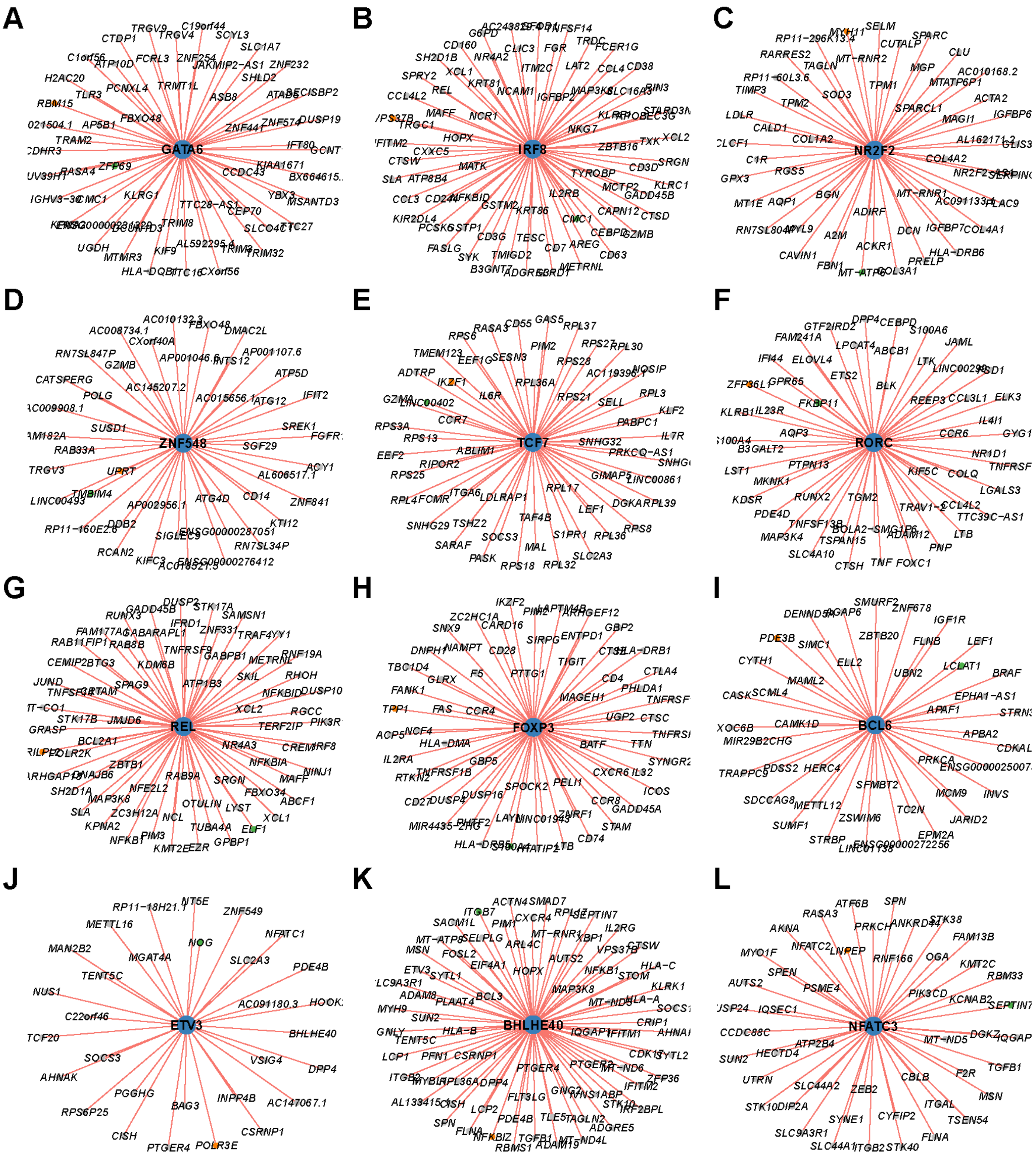

Figure S24. TF–target regulon networks for representative T-cell transcription factors.

(A–L) Regulon network visualization for GATA6 (A), IRF8 (B), NRF2/NFE2L2 (C), ZNF548 (D), TCF7 (E), RORC (F), REL (G), FOXP3 (H), BCL6 (I), ETV3 (J), BHLHE40 (K), and NFATC3 (L). The central blue node denotes the TF, surrounding nodes denote inferred target genes, and red edges represent predicted regulatory links. Target genes shown correspond to the top-ranked regulon members.

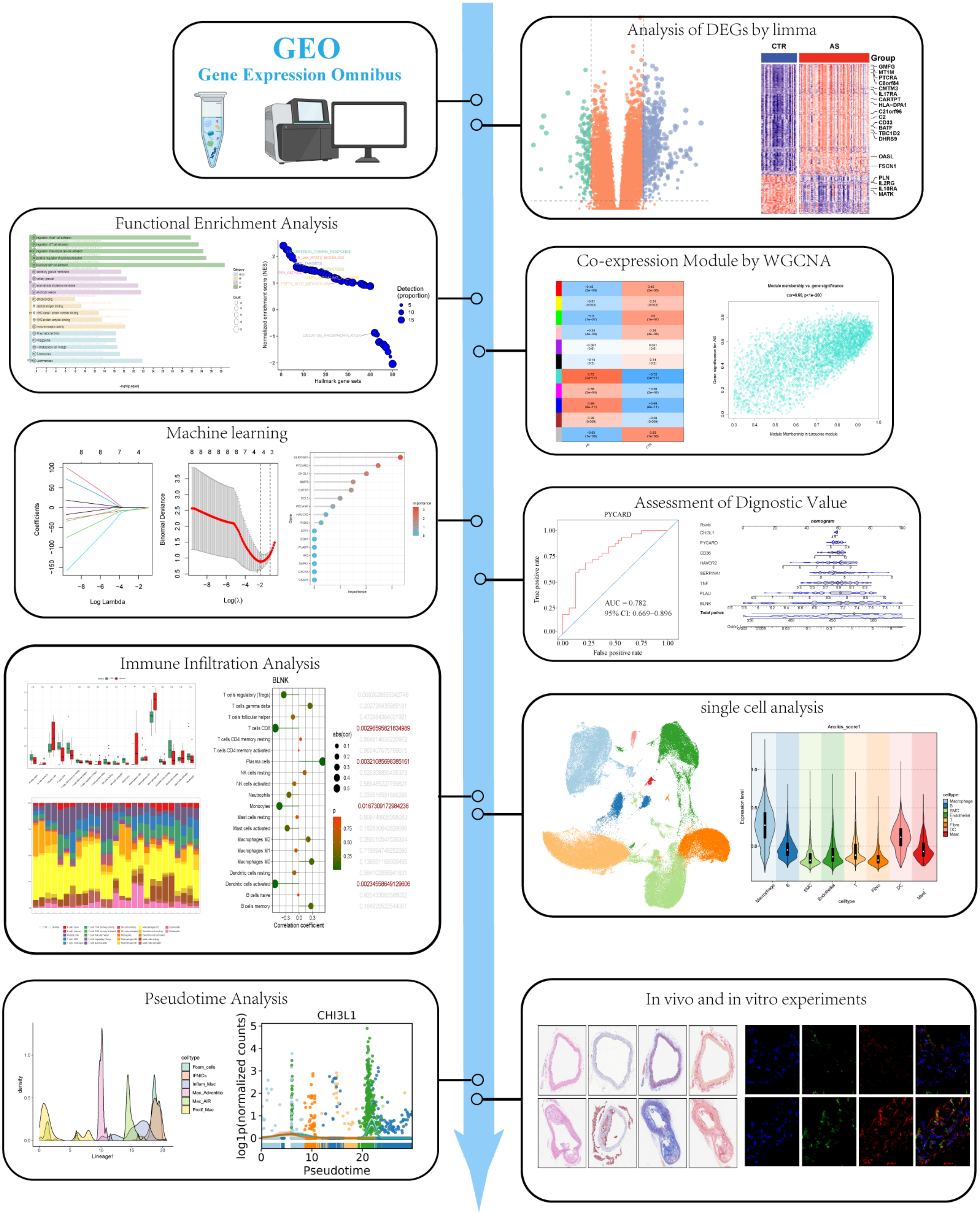

Graphical abstract. A Research Workflow Unveiling Macrophage-Driven Anoikis Hubs in Atherosclerosis

This integrative study establishes a comprehensive transcriptomic and experimental pipeline to decode anoikis-driven mechanisms in AS. First, global transcriptomic profiling and WGCNA identified a core immunometabolic module driving AS pathogenesis. By intersecting these signatures with ARGs, machine-learning algorithms (LASSO and RF) pinpointed robust diagnostic hub genes, which were consolidated into a highly accurate, clinically translatable nomogram. Next, immune deconvolution and sscRNA-seq mapped the lesional microenvironment, strictly localizing elevated anoikis activity to the

expanded macrophage compartment. Pseudotime trajectory inference further unveiled a dynamic state transition toward terminally differentiated foam cells, orchestrated by stage-specific transcriptional regulatory handoffs. Finally, in vitro (oxLDL stimulation) and in vivo ($ApoE^{-/-}$ models) experiments biologically validated the prioritized hubs (e.g., HAVCR2) as critical lipid-responsive drivers, mechanistically linking anoikis activation to macrophage-rich plaque progression.
